# Arylation of Olefins with N‑Unprotected Bromobisindole Ethanamines: Expanding the Scope of the Mizoroki–Heck Reaction

**DOI:** 10.1021/acs.joc.6c00302

**Published:** 2026-04-15

**Authors:** Alessandro Buono, Lorenzo Mariani, Martina Chiappini, Andrea Duranti, Diego Olivieri, Simone Lucarini

**Affiliations:** Department of Biomolecular Sciences, 19044 University of Urbino Carlo Bo, Campus Scientifico E. Mattei, via Ca’ le Suore 2, Urbino, PU 61029, Italy

## Abstract

A protecting-group-free Mizoroki–Heck arylation of olefins with N-unprotected bromobisindole ethanamines and, more generally, N-unprotected heterocyclic electrophiles is described. Efficient palladium-catalyzed coupling is achieved through appropriate base selection: CsF is optimal for substrates bearing intrinsic basic sites, whereas 1-decylamine is required. The method tolerates a wide range of styrenes, heteroaryl olefins, acrylates, and N-containing substrates, including unprotected tryptamines, anilines, and haloindole derivatives. Its practicality is also demonstrated by the synthesis of the natural product *trans*-resveratrol.

## Graphical abstract

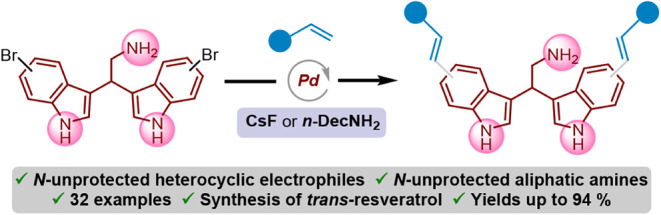

## Introduction

One of the most investigated cross-coupling processes^1^ is the Mizoroki–Heck (or just Heck) reaction, which is essentially the arylation (or vinylation) of an alkene with an organic halide^2^ in the presence of transition metal catalysis (usually palladium). Here, a coupling between two sp^2^ carbon centers takes place under basic conditions, which are necessary to neutralize the hydrogen halide formed during the reaction. This reaction is employed in many syntheses, including the production of commercial drugs and active ingredients.^3^ Nowadays, developing processes with a high functional group tolerance is one of the challenges of cross-coupling reactions, including the Heck reaction, as it would avoid synthetic steps for the insertion/removal of protecting groups. This is of particular interest for medicinal chemistry and the pharmaceutical industry, as, for example, nitrogen atoms are contained in a variety of drugs.^4^ However, just a few cases of the Heck reaction applied to unprotected amine-containing substrates have been reported.^5^ For example, recently, Young and coworkers reported the Heck monoarylation of free allylamines, although they utilized CO_2_, which served as an *in situ*-protecting group for the amine.^6^ More often, when NH_2_ free amino groups are present, especially on the electrophilic substrate, these act as directing groups^7^ or as precursors for the formation of arenediazonium salts (i.e., Heck–Matsuda coupling).^8^ The main problem of having an unprotected nitrogen is its capability to coordinate palladium, either by deactivating the catalyst or promoting undesired side reactions, resulting in a drastic drop in yields and selectivities. This is particularly true when compounds such as indoles or tryptamine derivatives are utilized without N-protecting groups, as it is widely known that both the indole NH group and the NH_2_ can coordinate palladium.^9^ On the other hand, these scaffolds are particularly useful in medicinal chemistry,^10^ and efforts have been made to overcome this problem. Although with unprotected haloindoles many examples can be found (Scheme 1a),^11,12^ only Sewald et al.^13^ and Goss et al.^12^ succeeded in the Mizoroki–Heck reaction of olefins with halotryptophans (Scheme 1b), even though the nucleophilicity of the amino group in tryptophan derivatives is attenuated since the molecule is in its zwitterionic form,^14^ and no example is reported with more challenging tryptamines. Among tryptamine derivatives, bisindole ethanamines constitute a prominent class of biologically active scaffolds, displaying antiviral,^15^ anticancer,^16^ antimicrobial,^17^ antifungal,^18^ antibiofilm, and antibiotic adjuvant properties.^19^ Over the years, our group has developed expertise in the synthesis of a variety of bisindole derivatives,^20^ prompted by their promising antileishmanial activity.^21^ In this context, the natural marine bisindole alkaloid 2,2-bis­(6-bromo-1*H*-indol-3-yl) ethanamine **1a** was active against *L. infantum* protozoa but also exhibited non-negligible toxicity, likely associated with the presence of bromine substituents.^22^ Recently, we have developed a rapid Suzuki–Miyaura coupling of **1a** with a range of aryl- and vinylboronic acids, enabling the synthesis of a small library of arylated bisindoles that showed promising antileishmanial activity and reduced human cytotoxicity.^23^ Encouraged by this result, we decided to investigate the Mizoroki–Heck coupling for the arylation of various olefins with NH- and NH_2_-unprotected bromobisindole ethanamines (Scheme 1c). In particular, we aimed to develop a practical protocol combining low catalyst loadings with tolerance toward unprotected ethylamine-containing haloindoles, since previous systems^11–13^ often require high catalyst loadings and do not address compatibility with aliphatic amines. In addition, besides these electrophilic substrates, other heterocyclic electrophiles bearing nitrogen atoms will be tested, hopefully developing a set of conditions that overcome some of the limitations of the reaction.

**Scheme 1:**
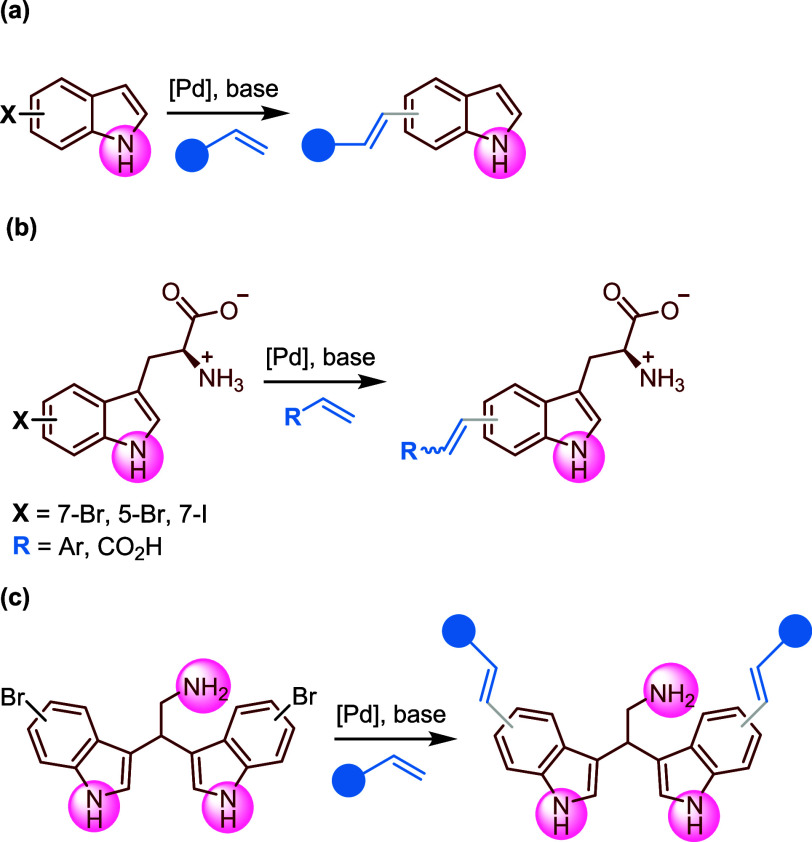
Mizoroki–Heck Coupling of Unprotected (a) Haloindoles, (b) Halotryptophans (Sewald 2019, Goss 2019), and (c) Bromobisindoles (this work)

## Results and Discussion

Our screening began with utilizing the conditions previously developed for our Suzuki–Miyaura coupling.^23^ However, when the aryl boronic acid was replaced with styrene **2a**, no product was observed. By raising both the temperature (up to 100 °C) and the reaction time, the desired product **3aa** was obtained in a decent amount (45%) together with the monofunctionalized compound **4aa** (Table 1, entry 1). When 20 mol% of tetrabutylammonium bromide (NBu_4_Br) was used, complete conversion was observed with higher yield and selectivity for **3aa** (entry 2). It is indeed known that ammonium salts generally exert a positive effect on Heck reactions, primarily by facilitating the formation of Pd(0) from the palladium-hydride species,^24^ also preventing the aggregation of Pd(0) particles, which would eventually lead to palladium black,^25^ and favoring the phase transfer of various species between the organic and aqueous media.^24^ Other bases have been then tested (entries 4–6) and, in particular, with CsF similar results to Cs_2_CO_3_ have been obtained (entry 6), possibly indicating a positive role of the cesium cation in the reaction.^26^ Moreover, the reaction also smoothly proceeds in the absence of an external base (entry 7), likely because the amino group of bisindole **1a** acts as a base itself. Despite various palladium catalysts having been tried, the desired product **3aa** is essentially achieved only with complexes bearing bidentate phosphine ligands (see Supporting Information), probably because these ancillary ligands force the olefin and the encumbered substrate **1a** into a favorable *cis*-position, which is essential for the reaction to proceed. Notably, with Pd­(BINAP)­Cl_2_ results similar to those obtained with Pd­(dppf)­Cl_2_ are reachable (entry 8). However, a difference between the two catalysts became evident when the reaction time was reduced, as the same conversion and **3aa** yield were obtained with Pd­(BINAP)­Cl_2_ in 4 h (entry 9), indicating a higher TOF^27^ for this catalyst. Finally, using Pd­(BINAP)­Cl_2_ and CsF as the base, full **1a** conversion and an 81% isolated yield of **3aa** were obtained in just 2 h (entry 10). This suggests that fluoride, in addition to acting as a weak base, may exert additional effects, such as stabilizing metal intermediates and promoting both hydrogen removal during the catalytic cycle^28^ and Pd(0) formation from the Pd­(II) precursor.^29^ Interestingly, although with other ammonium salts no beneficial effects have been observed (see Supporting Information), almost identical results were obtained by replacing NBu_4_Br with NBu_4_F (compare entries 10 and 11). Other variations, such as changing the olefin amount, the solvent system, the temperature, the tetrabutylammonium salt amount, the fluoride source, or the amount of CsF, did not lead to any improvement (see Supporting Information). Based on these results and literature data,^2^ a plausible catalytic cycle is reported in the Supporting Information. Testing other olefins, we observed that generally better results (i.e., higher yields with lower reaction times) were achieved with NBu_4_F with respect to NBu_4_Br, and therefore we decided to investigate the scope of the olefin employing the conditions of Table 1, entry 11.

**Table 1:**
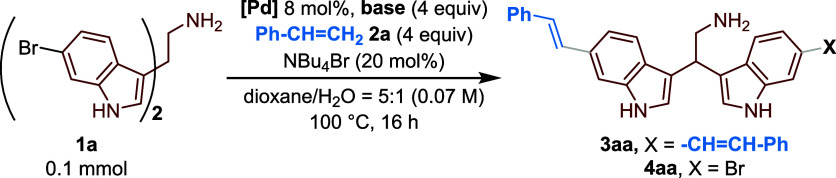
Reaction Optimizations

Entry	[Pd]	Base	Yield **3aa** [%]^[^ ^a^ ^]^	Yield **4aa** [%]^[^ ^a^ ^]^
1^[^ ^b^ ^,^ ^c^ ^,^ ^d^ ^]^	Pd(dppf)Cl_2_·DCM	Cs_2_CO_3_	45	14
2^[^ ^b^ ^]^	Pd(dppf)Cl_2_·DCM	Cs_2_CO_3_	60	10
3	Pd(dppf)Cl_2_·DCM	Cs_2_CO_3_	70 (67^[^ ^e^ ^]^)	<2
4	Pd(dppf)Cl_2_·DCM	K_2_CO_3_	63	3
5	Pd(dppf)Cl_2_·DCM	KOAc	47	7
6	Pd(dppf)Cl_2_·DCM	CsF	70 (65^[^ ^e^ ^]^)	<2
7^[^ ^f^ ^]^	Pd(dppf)Cl_2_·DCM	–	20	21
8	Pd(BINAP)Cl_2_	Cs_2_CO_3_	69 (66^[^ ^e^ ^]^)	<2
9^[^ ^g^ ^]^	Pd(BINAP)Cl_2_	Cs_2_CO_3_	64	3
10^[^ ^h^ ^]^	Pd(BINAP)Cl_2_	CsF	81 (80^[^ ^e^ ^]^)	3
11^[^ ^h^ ^,^ ^i^ ^]^	Pd(BINAP)Cl_2_	CsF	81 (81^[^ ^e^ ^]^)	3

a Determined by ^1^H NMR analysis. Unless otherwise noted, **1a** was fully converted.

b dioxane/H_2_O = 4:1 was utilized as the reaction mixture with a **1a** concentration of 0.05 M.

c No NBu_4_Br was utilized.

d **1a** conversion = 92%.

e Isolated yields.

f 1a conversion = 50%.

g Reaction time = 4 h.

h Reaction time = 2 h.

i 20 mol% of NBu_4_F was utilized instead of NBu_4_Br.

With the optimized conditions in hand, the position of the bromine atom on the bisindole scaffold was varied. In particular, with 5-bromobisindole **1b** and 7-bromobisindole **1c**, still good yields were achieved (86% and 75%, respectively), while employing 4-bromobisindole **1d**, only 20% conversion was observed with no product formation even after 18 h (Scheme 2), probably due to the presence of the ethylenimine group that sterically prevents the coupling.^30^ Then, the generality of the reaction was investigated, starting by coupling different olefins with 6-bromobisindole **1a** (Table 2). Using *para*-, *meta*-, and *ortho*-substituted styrenes, moderate to high yields were obtained despite the presence of EDGs and EWGs, and the coupling with 2-vinylnaphthalene also proceeded efficiently (**3am**, 53%).

**Scheme 2:**
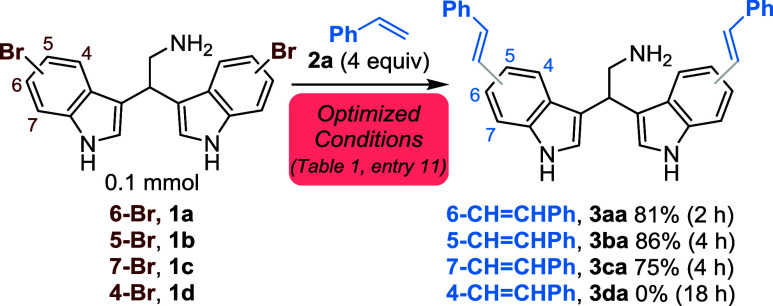
Mizoroki–Heck Coupling of Unprotected Bromobisindoles **1** with Styrene **2a**

**Table 2:**
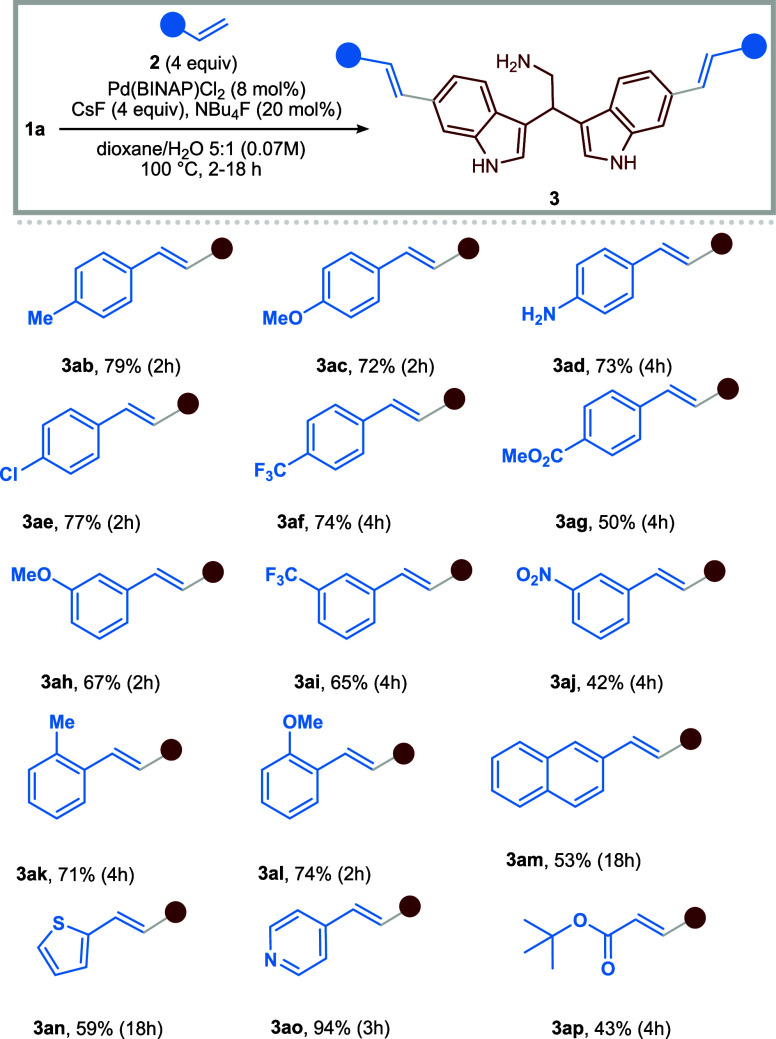
Scope of the Pd-Catalyzed Mizoroki–Heck Coupling of Unprotected Bisindole **1a** ^a^

a Isolated yields are reported.

From the results reported above, no clear or predictable reactivity trend emerges as a function of the substituents on the styrenes. Rather, the methodology appears broadly tolerant toward a wide range of steric and electronic factors, and in most cases, satisfactory yields are obtained regardless of the olefin employed. However, overall, the trend suggests that in the presence of strongly electron-withdrawing substituents—such as nitro or benzoate groups—the reaction tends to proceed less efficiently. Gratifyingly, heteroaryl olefins, namely 2-vinylthiophene and 4-vinylpyridine, afforded the desired product in good (**3an**, 59%) and excellent (**3ao**, 94%) yields, respectively.

Unfortunately, with 4-vinylphenyl acetate, product degradation was observed at 100 °C, while utilizing 1,2-disubstituted allyl alcohols, such as crotyl alcohol, or 1,1-disubstituted styrenes, such as α-methylstyrene, no reaction was observed. Finally, although the utilization of the α,β-unsaturated ketone but-3-en-2-one gave no product, to our great satisfaction, we were able to extend the methodology to the *tert*-butyl acrylate, providing product **3ap** in 43% yield. To further broaden the scope of the reaction, besides bisindoles **1a**–**1d** our developed Mizoroki–Heck coupling was also tested with other electrophiles bearing various heteroatoms. Surprisingly, when 6-bromoindole **1e** and styrene **2a** were used under optimized conditions, no product was observed. This unexpected outcome prompted us to investigate the reason behind it, since **1a** contains a 6-bromoindole motif, and thus, a similar reactivity would have been expected. We found that the choice of base was crucial, as the desired product was obtained only using organic amines (see Supporting Information), and the best result was achieved utilizing 1-decylamine (*n*-DecNH_2_). However, testing this base with previous substrates led to comparable or worse results (see Supporting Information). Therefore, to better understand this behavior, various electrophiles were coupled with styrene **2a** either with CsF or with *n*-DecNH_2_, and both results are reported in Table 3. The scope was first extended to nitrogen-containing heteroaryl bromides. With 3-bromopyridine, CsF proved highly effective, affording **5fa** in 91% yield within 2 h, whereas decylamine resulted in only 14% yield. A similar trend was observed for quinoline derivatives, which provided **5ga** and **5ha** in 48% and 71% yield with CsF, respectively, but significantly lower yields with decylamine. In contrast, less strongly coordinating and basic substrates showed the opposite behavior: unprotected anilines and 4-bromobenzamide gave higher yields with decylamine (93% and 55%, respectively), while CsF was ineffective or poorly productive. A particularly noteworthy result was obtained with completely NH- and NH_2_-unprotected tryptamine. For the first time, to the best of our knowledge, 6-bromotryptamine was gratifyingly converted into desired product **5ka** in 55% yield with CsF, whereas a substantially similar yield of 60% was obtained under decylamine conditions, thus demonstrating that the methodology can be successfully applied even to highly sensitive, unprotected tryptamine-based scaffolds. The secondary amine-containing bisindole **5la** was reactive only in the presence of decylamine, while 2-bromothiophene gave a moderate yield only with CsF. Using 2-(3-bromophenyl)­ethan-1-amine (**1n**), the corresponding Heck product **5na** is obtained in high yields with both CsF and *n*-DecNH_2_, with the former providing better results (i.e., 94% isolated yield). The study was then broadened to an indole-based electrophile bearing various (pseudo)­halogens. While no reaction was observed using 6-chloroindole with either base, the use of 6-iodoindole and 6-indolyl triflate (not previously reported in this type of transformation) enabled successful coupling in the presence of decylamine, affording the corresponding product **5ea** in 70% and 20% yield, respectively. Notably, the entire substrate scope could be addressed using a relatively low catalyst loading (4 mol% Pd­(BINAP)­Cl_2_ for each reaction site), underscoring the intrinsic efficiency of the catalytic system and the robustness of the methodology. Based on the results described above, decylamine appears to be required when the substrate lacks an intrinsic Lewis basic site.^31^ To prove our hypothesis, the basic NH_2_ functionality of **1a** was acetylated, achieving compound **1o**, which is coupled with **2a** both in the presence of CsF and decylamine. As expected, the desired product is obtained in significantly higher yields only with the latter base (Table 3C). Eventually, the synthesis of *trans*-resveratrol (1 mmol scale), which possesses various well-known biologically relevant activities,^32^ was performed using our methodology. In particular, by coupling 1-bromo-3,5-dimethoxybenzene **1p,** and 4-vinylanisole **2c**, utilizing decylamine as the base, the *trans*-trimethoxyresveratrol **5pc** is obtained with 70% isolated yield, which furnished the desired natural product after treatment with BBr_3_.^33^ (Table 3D).

**Table 3:**
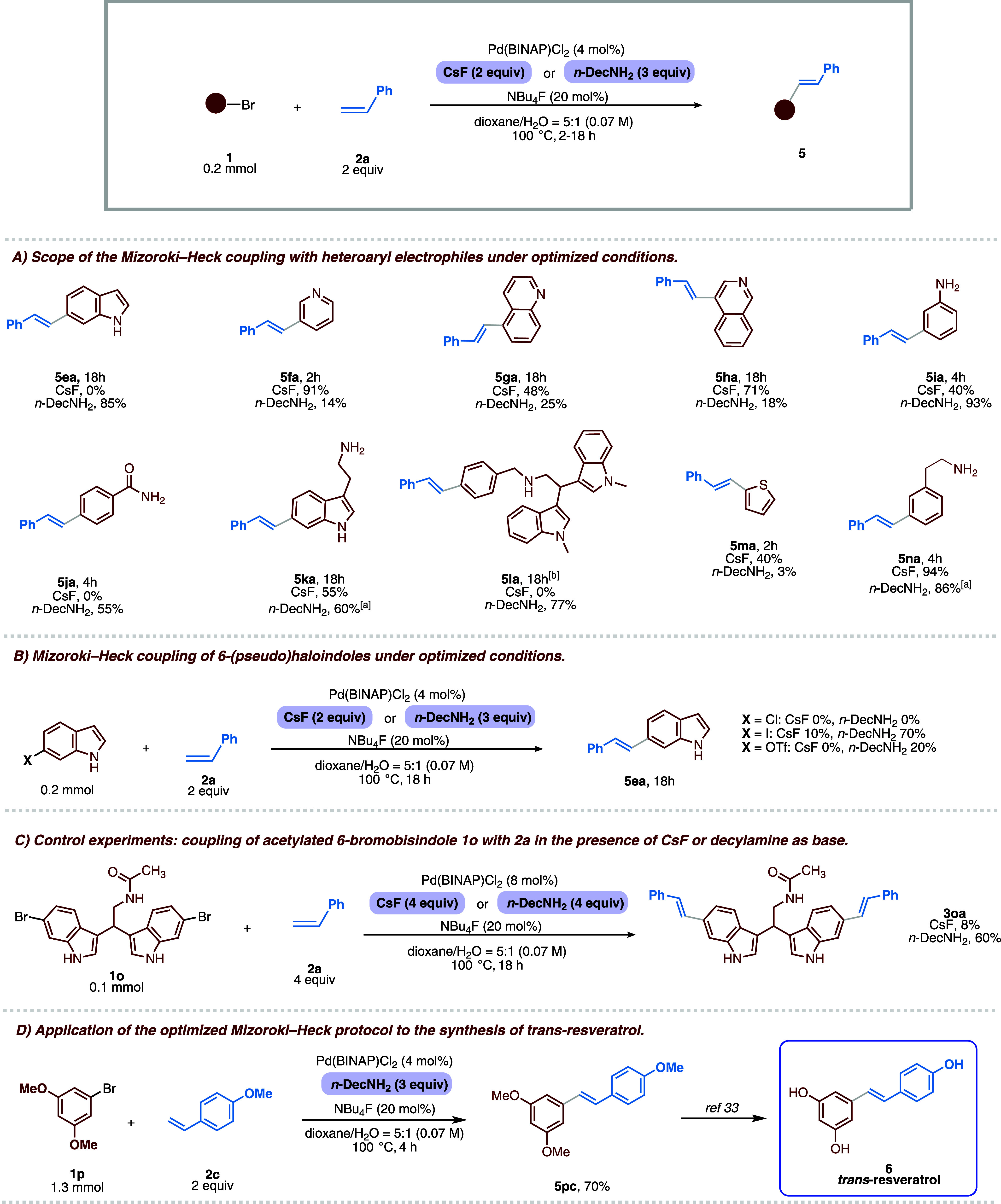
Electrophile Scope and Limitations of the Developed Mizoroki–Heck Reaction

a 2 equiv (0.4 mmol) of *n*-DecNH_2_ were utilized.

b Reaction was performed on a 0.1 mmol scale.

In conclusion, a general and efficient Mizoroki–Heck coupling of olefins with unprotected nitrogen-containing electrophiles has been developed, with particular emphasis on NH- and NH_2_-unprotected bromobisindole ethanamines. The study reveals a clear substrate-dependent base effect, whereby CsF is optimal for electrophiles containing basic nitrogen atoms, while 1-decylamine is essential for substrates lacking intrinsic basicity. The protocol exhibits broad functional-group tolerance, low catalyst loading with respect to the usually reported protocols, and applicability to structurally complex indole-based scaffolds. Given the prevalence of nitrogen atoms in pharmaceutically relevant molecules, these findings significantly expand the synthetic utility of Mizoroki–Heck reactions under protecting-group-free conditions.

## Supplementary Material



## Data Availability

The data underlying this study are available in the published article and its Supporting Information.

## Notes

Experimental Section; compound characterization; and copies of ^1^H, ^13^C, ^19^F NMR spectra (PDF)
