# Supplementary material for: Arylation of Olefins with N‑Unprotected Bromobisindole Ethanamines: Expanding the Scope of the Mizoroki–Heck Reaction
Source: J Org Chem. 2026 Apr 15;91(16):5591–7. doi: 10.1021/acs.joc.6c00302 (PMC13122646; doi:10.1021/acs.joc.6c00302)
Supplement: Supplementary file 1 [file jo6c00302_si_001.pdf]

# Arylation of Olefins with *N*-Unprotected Bromobisindole Ethanamines: Expanding the Scope of the Mizoroki–Heck Reaction

Alessandro Buono,[a] Lorenzo Mariani,[a] Martina Chiappini,[a] Andrea Duranti,[a] Diego Olivieri,[a] Simone Lucarini*[a]

[a] A. Buono, L. Mariani, M. Chiappini, Dr. A. Duranti, Dr. D. Olivieri, Dr. S. Lucarini
Department of Biomolecular Sciences, Section of Chemistry and Pharmaceutical Technologies
University of Urbino Carlo Bo
Campus Scientifico E. Mattei, via Ca' le Suore 2, 61029 Urbino, PU, Italy
E-mail: simone.lucarini@uniurb.it

# Supporting Information

## Contents:

1. Material and methods S2
2. Charts of starting materials S4
3. Complete Optimization Reactions for the Mizoroki-Heck Coupling of Unprotected Bromobisindole S5
4. Proposed Catalytic Cycle S9
5. Effect of organic bases on the developed Mizoroki-Heck reaction S10
6. Syntheses of the Starting Materials **1l** and **1o** S12
7. Synthesis of *trans*-resveratrol S14
8. Experimental data for compounds **3** and **5** S16
9. NMR Spectra S28
10. References S61

# 1. Material and methods

All reactions were prepared under nitrogen atmosphere with dry solvents under anhydrous conditions, by using Schlenk technique. Reactions were monitored by $^{1}H$ NMR taking a direct sample of the crude mixture and using 1,3,5-trimethoxybenzene as standard. $^{1}H$ NMR and $^{13}C$ NMR were recorded on a Bruker Avance 400 spectrometer ($^{1}H$: 400 MHz, $^{13}C$: 101 MHz, $^{19}F$: 376 MHz) or on a Bruker Avance Neo 600 spectrometer ($^{1}H$: 600 MHz, $^{13}C$: 151 MHz, $^{19}F$: 565 MHz) using DMSO-$d_6$, $CDCl_3$ or $CD_3OD$ as solvent. Chemical shifts are reported in the $\delta$ scale relative to residual $CHCl_3$ (s, 7.26 ppm), DMSO (p, 2.50 ppm), $CD_3OD$ (p, 3.31 ppm) for $^{1}H$ NMR and to the central line of $CDCl_3$ (77.16 ppm), DMSO-$d_6$ (39.52 ppm) $CD_3OD$ (49.00 ppm) for $^{13}C$ NMR. $^{13}C$ NMR and $^{19}F$ NMR were recorded with $^{1}H$ broadband decoupling. The following abbreviations were used to explain the multiplicities: br = broad, s = singlet, d = doublet, t = triplet, q = quartet, dd = doublet of doublets, ddd = doublet of doublets of doublets, m = multiplet. Coupling constants (*J*) are reported in Hertz (Hz). HRMS spectra were performed by slow direct infusion (5 μL/min) of ≈ 0.1 μg/mL solution (methanol), using Orbitrap Exploris 240 mass spectrometer. Only molecular ions $[M + H]^+$ are given. For compound **3ca** the molecular ion was detected as $[M + K]^+$, whereas for compound **3oa** the molecular ion was observed as $[M + Na]^+$. 1,4-dioxane was dried over molecular sieves (Alfa Aesar, 4 Å, 1–2 mm, beads). Both 1,4-dioxane and water were degassed ($N_2$ bubbling for 30 min). Pure compounds **3** and **5** were isolated through flash column chromatography on silica gel 60 (40-60 μm, 230-400 mesh). Olefins **2** were purchased from Merck Sigma-Aldrich, BLD Pharma or TCI. Liquid purchased olefins were filtered off a plug of neutral $Al_2O_3$ and used without further purification. Pd(Xantphos)$Cl_2$[1], Pd(DPEPhos)$Cl_2$[1], Pd(dppe)$Cl_2$[2], Pd(dppp)$Cl_2$[2] and Pd(BINAP)$Cl_2$[3] have been synthesized as already described in the literature. **SM1** has been synthesized as already described in the literature.[4] Bromobisindole derivatives **1a**,[5] **1b**,[5] **1c**[6] and **1d**[6] have been synthesized as already described in the literature. 6-bromotryptamine **1k** was synthesized as already described in the literature.[7] The new bromobisindole **1l**[8] and bisindole **1o**[9] have been realized adapting a literature procedure, as reported in this Supporting Information. All other chemicals were purchased from Merck Sigma-Aldrich and used without further purification. All solid reagents were weighed in an analytical balance without excluding moisture and air.

**General procedure 1 (GP1). Typical Procedure for the Mizoroki–Heck coupling of unprotected bromobisindole.**

Compound **1a-d** (0.1 mmol, 43.3 mg), Pd(BINAP)$Cl_2$ (8 mol%, 6.4 mg), CsF (0.4 mmol, 60.8 mg), $NBu_4F{\cdot}3H_2O$ (20 mol%, 6.3 mg) and the selected olefin **2** (0.4 mmol) were added in a dried Schlenk tube, equipped with a magnetic stirring bar. Degassed dioxane (1.20 mL) and degassed water (240 μL) were added, and the reaction was vigorously stirred at 100 °C in an oil bath under balloon pressure of $N_2$, monitoring the progress of the reaction by TLC. The reaction mixture was analyzed by $^1$H NMR to determine the conversion of the bisindole. The crude was filtered on silica plug (dichloromethane/methanol/ammonia, 80/20/1) and the products were eventually obtained after column chromatography on silica gel.

**General procedure 2. (GP2). Typical procedure for the Mizoroki–Heck coupling of heteroaryl electrophiles 1e-n.**

Compound **1e-n** (0.2 mmol), Pd(BINAP)$Cl_2$ (4 mol%, 6.4 mg), CsF (0.4 mmol, 60.8 mg) or 1-decylamine (0.6 mmol, 60 μL), $NBu_4F{\cdot}3H_2O$ (20 mol%, 12.6 mg) and the olefin **2a** (0.4 mmol, 46 μL) were added in a dried Schlenk tube, equipped with a magnetic stirring bar. Degassed dioxane (2.40 mL) and degassed water (480 μL) were added, and the reaction was vigorously stirred at 100 °C in an oil bath under balloon pressure of $N_2$, monitoring the progress of the reaction by TLC. The reaction mixture was analyzed by $^1$H NMR to determine the conversion of the starting material. The crude was filtered on silica plug (EtOAc) and the products were eventually obtained after column chromatography on silica gel.

For the synthesis of compounds **5ka** and **5na**, 0.4 mmol of 1-decylamine was used instead of 0.6 mmol.

For the synthesis of compound **5la**, the reaction was performed on a 0.1 mmol scale using **2a** (0.2 mmol), Pd(BINAP)$Cl_2$ (4 mol%), $NBu_4F{\cdot}3H_2O$ (20 mol%), CsF (0.2 mmol) or 1-decylamine (0.3 mmol), dioxane (1.20 mL) and water (240 μL).

**Synthesis of the best catalyst Pd(BINAP)$Cl_2$.**

The best catalyst Pd(BINAP)$Cl_2$ was synthesized following a literature procedure.[3] To a round-bottom flask was added $PdCl_2(MeCN)_2$ (124 mg, 0.48 mmol) and *rac*-BINAP (300 mg, 0.48 mmol), followed by addition of DCM (15 mL). The resulting solution was allowed to stir at room temperature for 24 h, then concentrated under vacuum to give Pd(BINAP)$Cl_2$ as a yellow solid; yield: 100% (384 mg). $^1$H NMR (400 MHz, DMSO-$d_6$): $\delta$ = 7.78-7.66 (m, 8H), 7.66-7.44 (m, 12H), 7.24-7.12 (m, 4H), 6.95 (dd, $J_1 \approx J_2$ = 7.5 Hz, 2H), 6.81-6.71 (m, 4H), 6.65 (d, $J$ = 8.5 Hz, 2H) ppm.

## 2. Charts of starting materials

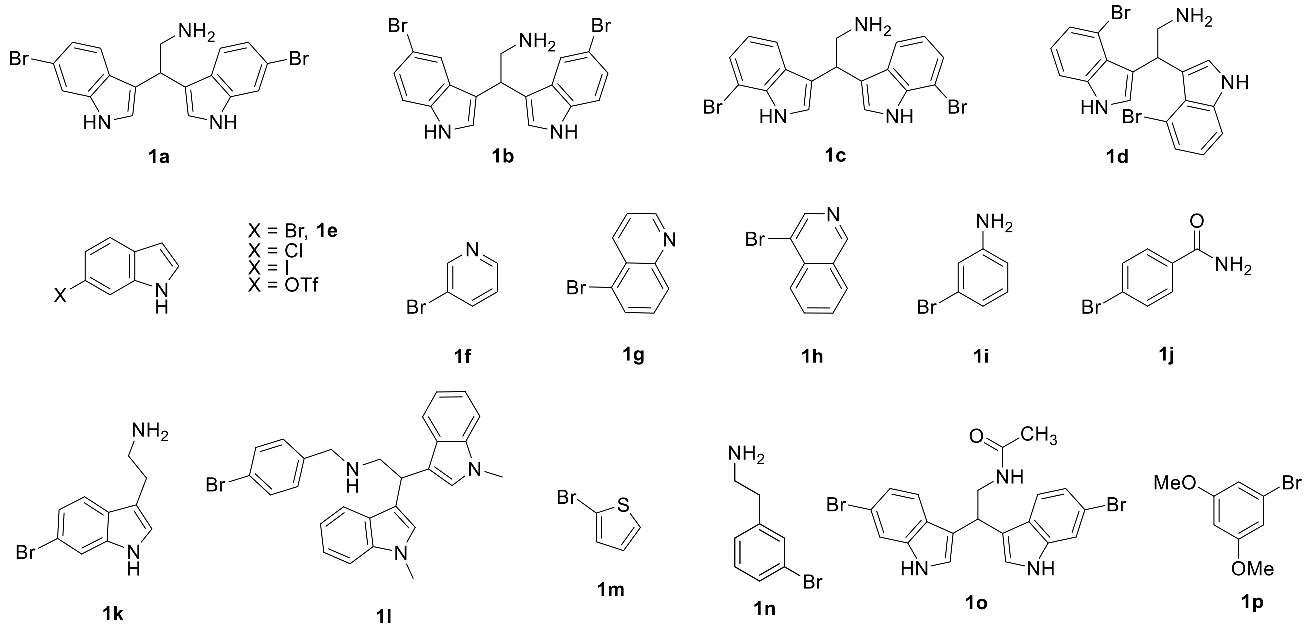


**Figure S1 –** Complete list of the utilized Br-(bis)indoles and heteroaryl electrophiles **1**

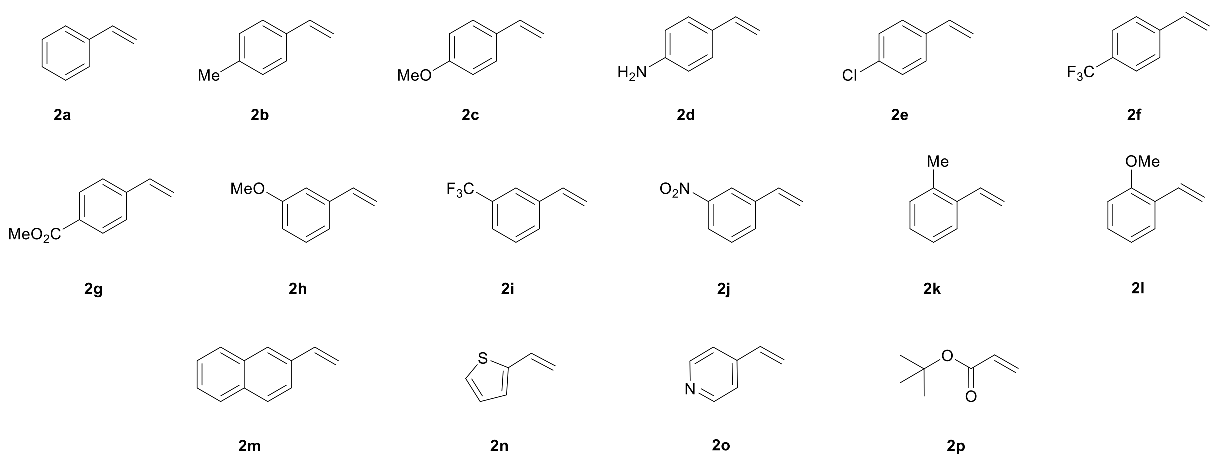


**Figure S2 –** Complete list of the utilized olefins **2**

# 3. Complete Optimization Reactions for the Mizoroki-Heck Coupling of Unprotected Bromobisindole

**Table S1 –** Screening of the solvent, the concentration and the ratio organic solvent : water

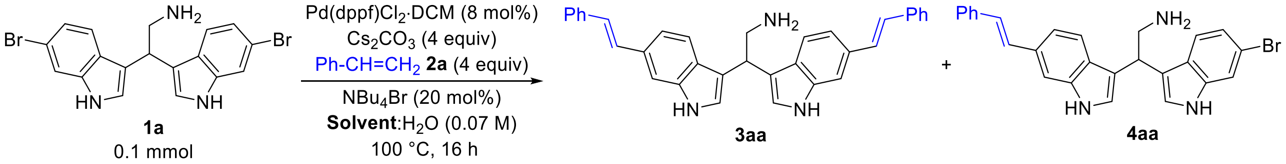


| Entry[a] | Solvent[b] | Solvent : $H_2O$ (v/v) | Conversion **1a** [%][c] | Yield **3aa** [%][c] | Yield **4aa** [%][c] |
|---|---|---|---|---|---|
| 1[d,e] | 1,4-dioxane | 4 : 1 | 92 | 45 | 14 |
| 2[e] | 1,4-dioxane | 4 : 1 | 100 | 60 | 10 |
| 3[e] | 1,4-dioxane | 5 : 1 | 100 | 61 | < 2 |
| 4 | 1,4-dioxane | 5 : 1 | 100 | 70 (67[f]) | < 2 |
| 5 | DMSO | 5 : 1 | 60 | 0 | 15 |
| 6 | DMF | 5 : 1 | 92 | 15 | 25 |
| 7 | 1,4-dioxane | 6 : 1 | 84 | 40 | 18 |
| 8 | 1,4-dioxane | 7 : 1 | 90 | 36 | 15 |
| 9[g] | 1,4-dioxane | 5 : 1 | 100 | 60 | 6 |
| 10[h] | 1,4-dioxane | 5 : 1 | 40 | < 2 | 10 |
| 11[i] | 1,4-dioxane | 5 : 1 | 50 | < 2 | 10 |
| 12[j] | EtOH | 5 : 1 | 74 | 43 | 9 |

[a] Reaction performed under $N_2$ inert atmosphere using bisindole **1a** (0.1 mmol), styrene **2a** (0.4 mmol), Pd(dppf)$Cl_2$·DCM (8 mol% of catalyst loading), $Cs_2CO_3$ (0.4 mmol) and NBu4Br (0.02 mmol), in the indicated reaction medium at 100°C for 16 h. [b] Solvents dried over molecular sieves. [c] Determined by $^1H$ NMR analysis of the reaction crude using 1,3,5-trimethoxybenzene as standard. [d] No $NBu_4Br$ was utilized. [e] **1a** concentration = 0.05 M [f] Isolated yield by means of chromatographic column. [g] **1a** concentration = 0.1 M. [h] DMF was utilized in place of $H_2O$. [i] DMSO was utilized in place of $H_2O$.[j] Reaction performed under $N_2$ inert atmosphere using bisindole **1a** (0.1 mmol), styrene **2a** (0.4 mmol), Pd(BINAP)$Cl_2$ (8 mol% of catalyst loading), CsF (0.4 mmol) and $NBu_4F$ (0.02 mmol), in the indicated reaction medium at 100°C for 16 h.

**Table S2 –** Screening of bases and additives

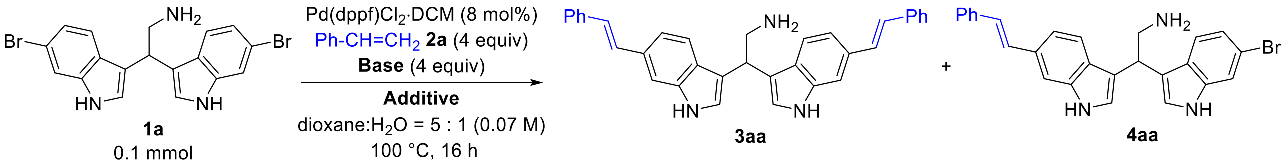


| Entry[a] | Base | Additive | Conversion **1a** [%][b] | Yield **3aa** [%][b] | Yield **4aa** [%][b] |
|---|---|---|---|---|---|
| 1 | $K_2CO_3$ | $NBu_4Br$ 20 mol% | 100 | 63 | 3 |
| 2 | KOAc | $NBu_4Br$ 20 mol% | 100 | 47 | 7 |
| 3 | CsF | $NBu_4Br$ 20 mol% | 100 | 70 (65[c]) | < 2 |
| 4 | $Cs_2CO_3$[d] | $NBu_4Br$ 20 mol% | 100 | 60 | 6 |
| 5[e] | $Cs_2CO_3$ | - | 87 | 37 | 32 |
| 6 | - | $NBu_4Br$ 20 mol% | 50 | 20 | 21 |
| 7 | $Cs_2CO_3$ | *p*-benzoquinone[f,g] 5 mol% | 83 | 40 | 24 |
| 8 | $Cs_2CO_3$ | KI[f,g] 1 equiv | 60 | 17 | 23 |
| 9 | $Cs_2CO_3$ | KCl[f] 1 equiv | 100 | 61 | 5 |
| 10 | $Cs_2CO_3$ | $NBu_4HSO_4$ 20 mol% | 95 | 42 | 20 |
| 11 | $Cs_2CO_3$ | $NBu_4I$ 20 mol% | 85 | 23 | 33 |
| 12 | $Cs_2CO_3$ | $NBu_4Cl$ 20 mol% | 100 | 51 | 8 |
| 13 | $Cs_2CO_3$ | $NBu_4Br$ 40 mol% | 100 | 60 | 4 |
| 14 | $Cs_2CO_3$ | $NBu_4Br$ 10 mol% | 98 | 50 | 5 |

[a] Reaction performed under $N_2$ inert atmosphere using bisindole **1a** (0.1 mmol), styrene **2a** (0.4 mmol), Pd(dppf)$Cl_2$·DCM (8 mol% of catalyst loading), using the indicated base (0.4 mmol) and additive in dioxane/$H_2O$ = 5 : 1 (0.07 M) at 100°C for 16 h. [b] Determined by $^1H$ NMR analysis of the reaction crude using 1,3,5-trimethoxybenzene as standard. [c[ Isolated yield by means of chromatographic column. [d] 2 equiv of base were utilized. [e] Reaction performed using 2.2 equiv of **2a** and in dioxane/$H_2O$ = 4 : 1 (0.05 M) as the reaction medium. [f] 20 mol% of $NBu_4Br$ were also added. [g] **1a** concentration = 0.05 M.

**Table S3 –** Screening of the catalyst

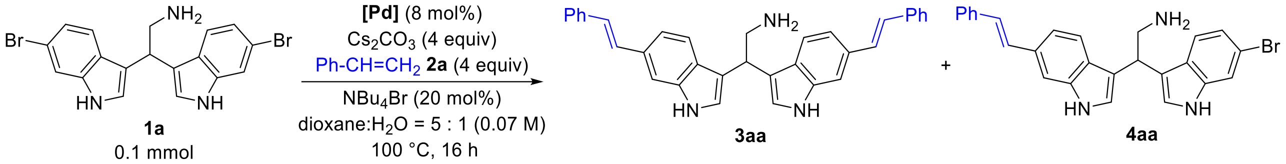


| Entry[a] | [Pd] source | Added Ligand | Conversion **1a** [%][b] | Yield **3aa** [%][b] | Yield **4aa** [%][b] |
|---|---|---|---|---|---|
| 1 | - | - | 30 | 0 | 0 |
| 2[c] | Pd(dppf)$Cl_2$·DCM | - | 87 | 39 | 20 |
| 3[d] | Pd(dppf)$Cl_2$·DCM | - | 100 | 51 | 3 |
| 4[e] | Pd(dppf)$Cl_2$·DCM | - | 35 | < 2 | 8 |
| 5[f] | Pd(dppf)$Cl_2$·DCM | - | 75 | 30 | 25 |
| 6 | $Pd_2dba_3$ | dppf 16 mol% | 77 | 16 | 11 |
| 7 | $Pd_2dba_3$[c] | dppf 16 mol% | 75 | 18 | 10 |
| 8[g] | $Pd(OAc)_2$ | dppf 16 mol% | 42 | 13 | 25 |
| 9[g] | $Pd_2dba_3$[c] | XPhos 30 mol% | 43 | < 2 | 30 |
| 10 | $Pd_2dba_3$[c] | SPhos 10 mol% | 51 | 0 | 5 |
| 11 | $PdCl_2[P(o\text{-Tol})_3]_2$ | - | 43 | 0 | 15 |
| 12 | BrettPhos Pd G3 | - | 75 | 8 | n.d. |
| 13 | RuPhos Pd G2 | - | 68 | 4 | n.d. |
| 14 | PEPPSI™-SIPr catalyst | - | 42 | < 2 | 20 |
| 15 | Pd(Xantphos)$Cl_2$ | - | 92 | 31 | 16 |
| 16 | Pd(DPEPhos)$Cl_2$ | - | 100 | 55 | < 2 |
| 17 | Pd(dppe)$Cl_2$ | - | 89 | 26 | 20 |
| 18 | Pd(dppp)$Cl_2$ | - | 99 | 24 | 25 |
| 19 | Pd(BINAP)$Cl_2$ | - | 100 | 69 (66[h]) | < 2 |
| 20[i] | Pd(BINAP)$Cl_2$ | - | 100 | 64 | 3 |

[a] Reaction performed under $N_2$ inert atmosphere using bisindole **1a** (0.1 mmol), styrene **2a** (0.4 mmol), the indicated Pd source (8 mol% of catalyst loading) and the indicated ligand in the given amount (when externally added), $Cs_2CO_3$ (0.4 mmol) and NBu4Br (0.02 mmol), in dioxane/$H_2O$ = 5 : 1 (0.07 M) as the reaction medium at 100°C for 16 h. [b] Determined by $^1$H NMR analysis of the reaction crude using 1,3,5-trimethoxybenzene as standard. [c] catalyst loading = 4 mol%. [d] catalyst loading = 16 mol%. [e] Temperature = 80 °C. [f] = Reaction time = 6 h. [g] 2.2 equiv of olefin **2a** were utilized. [h] Isolated yield by means of chromatographic column. [i] Reaction time = 4 h. n.d. = not determinable.

**Table S4 –** Screening of bases and additives containing the fluorine anion with the best catalyst Pd(BINAP)$Cl_2$

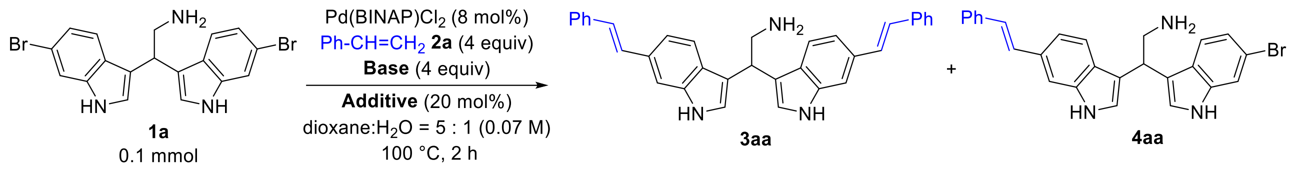


| Entry[a] | Base | Additive | Conversion **1a** [%][b] | Yield **3aa** [%][b] | Yield **4aa** [%][b] |
|---|---|---|---|---|---|
| 1 | CsF | $NBu_4Br$ | 100 | 81 (80[c]) | 3 |
| 2 | NaF | $NBu_4Br$ | 30 | < 5 | 9 |
| 3 | CsCl | $NBu_4Br$ | 60 | < 5 | 18 |
| 4 | CsF[d] | $NBu_4Br$ | 89 | 29 | 27 |
| 5 | CsF | $NBu_4F$ | 100 | 81 (81[c]) | 3 |
| 6 | $NBu_4F$ | - | 100 | 61 | 12 |

[a] Reaction performed under $N_2$ inert atmosphere using bisindole **1a** (0.1 mmol), styrene **2a** (0.4 mmol), Pd(BINAP)$Cl_2$ (8 mol% of catalyst loading), using the indicated base (0.4 mmol) and additive (0.02 mol) in dioxane/$H_2O$ = 5 : 1 (0.07 M) at 100°C for 2 h, [b] Determined by $^1$H NMR analysis of the reaction crude using 1,3,5-trimethoxybenzene as standard. [c] Isolated yield by means of chromatographic column. [d] 2.5 equiv of base were utilized

# 4. Proposed Catalytic Cycle

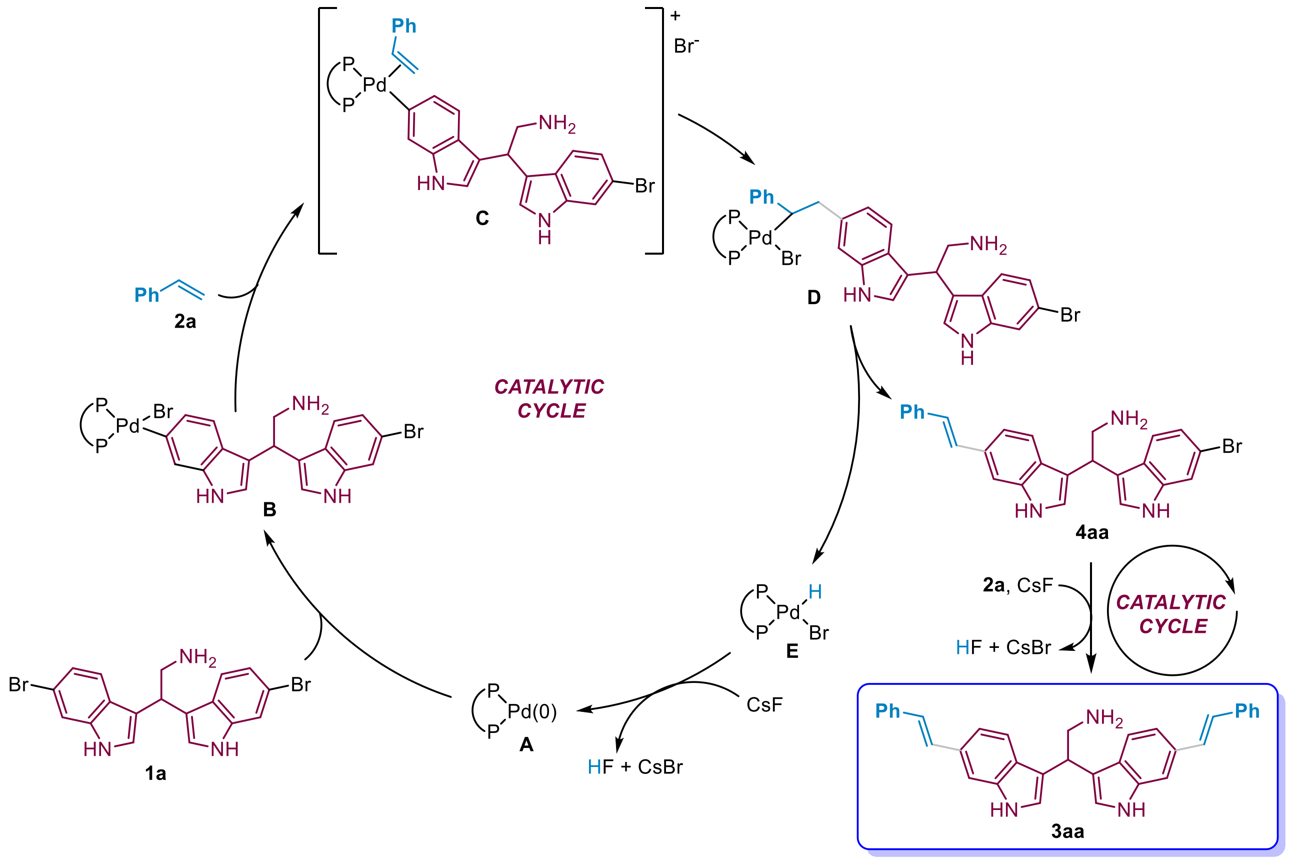


**Scheme S1 –** Proposed catalytic cycle

## 5. Effect of organic bases on the developed Mizoroki-Heck reaction

**Table S5 –** Optimization of the reaction of 6-bromoindole **1e** and styrene **2a**

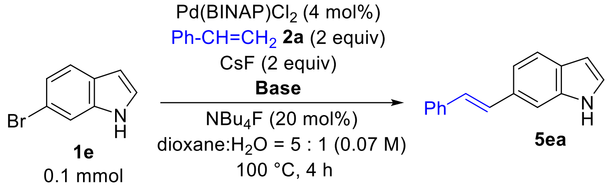


| Entry[a] | Organic Base | Organic base amount (equiv) | Conversion **1e** [%][b] | Yield **5ea** [%][b] |
|---|---|---|---|---|
| 1 | - | - | 0 | 0 |
| 2 | TEA | 1 | 42 | 30 |
| 3 | Aniline | 1 | 47 | 21 |
| 4 | Benzylamine | 1 | 52 | 36 |
| 5[c] | TEA | 1 | 54 | 41 |
| 6[c] | Aniline | 1 | 59 | 46 |
| 7[c] | Benzylamine | 1 | 85 | 50 |
| 8[c] | Pyridine | 1 | 15 | 15 |
| 9[c] | 1-decylamine | 1 | 78 | 57 |
| 10[c,d] | Benzylamine | 1 | 45 | 35 |
| 11[c,d] | 1-decylamine | 1 | 47 | 38 |
| 12[c,d] | TEA | 3 | 75 | 27 |
| 13[c,d] | 1-decylamine | 3 | 100 | 85 |
| 14[c,d] | *n*-butylamine | 3 | 26 | 20 |
| 15[c,d] | Ethane 1,2-diamine | 3 | 45 | 15 |
| 16[c,d] | *i*-butylamine | 3 | 46 | 20 |
| 17[c,d] | cyclohexylamine | 3 | 68 | 40 |
| 18[c,d] | diethylamine | 3 | 23 | 19 |
| 19[c] | 1-decylamine | 0.5 | 68 | 56 |

[a] Reaction performed under $N_2$ inert atmosphere using indole **1e** (0.1 mmol), styrene **2a** (0.2 mmol), Pd(BINAP)$Cl_2$ (4 mol% of catalyst loading), CsF (0.2 mmol), the indicated organic base and $NBu_4F$ (0.02 mmol), in dioxane/$H_2O$ = 5:1 (0.07 M) as the reaction medium at 100°C for 4 h. [b] Determined by $^1H$ NMR analysis of the reaction crude using 1,3,5-trimethoxybenzene as standard. [c] Reaction time = 18 h. [d] No CsF was utilized

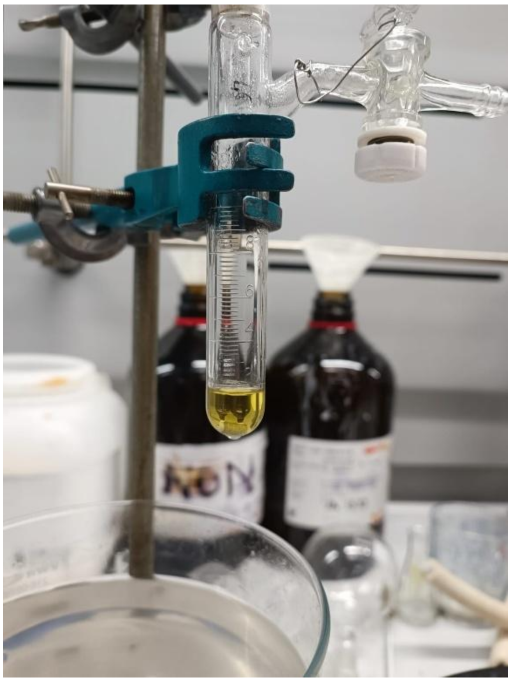

**Figure S3 –** Reaction mixture appearance at the end of the coupling when 1-decylamine was used as the base (Table S5**,** entry 13)

**Table S6 –** Comparison of CsF and 1-decylamine as base with bromobisindole **1a** and various olefins

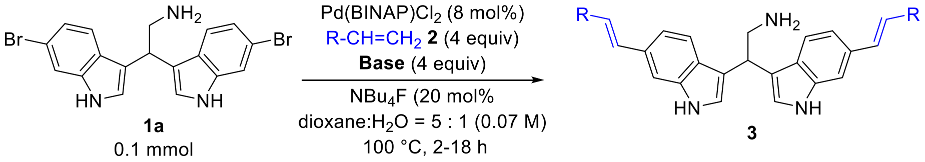


| Entry[a] | **2** | Reaction Time [h] | Yield **3** with CsF [%][b] | Yield **3** with *n*-DecNH$_2$ [%][b] |
|---|---|---|---|---|
| 1 | 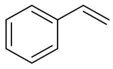 **2a** | 2 | 81 | 85 |
| 2 | 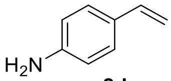 **2d** | 4 | 73 | 74 |
| 3 | 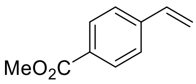 **2g** | 4 | 50 | 56 |
| 4 | 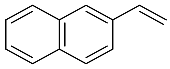 **2m** | 18 | 53 | 36 |
| 5 | 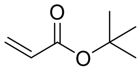 **2p** | 4 | 43 | 0 |

[a] Reaction performed under $N_2$ inert atmosphere using bromobisindole **1a** (0.1 mmol), olefin **2** (0.4 mmol), 8 mol% of Pd(BINAP)$Cl_2$, with the indicated base (0.4 mmol), $NBu_4F$ (20 mol %) in dioxane/$H_2O$ = 5:1 (0.07 M) at 100 °C for the indicated time [b] Isolated yield by means of chromatographic column.

## 6. Syntheses of the Starting Materials 1l and 1o

The synthesis of the new bromobisindole **1l** has been realized following a literature procedure.[8]

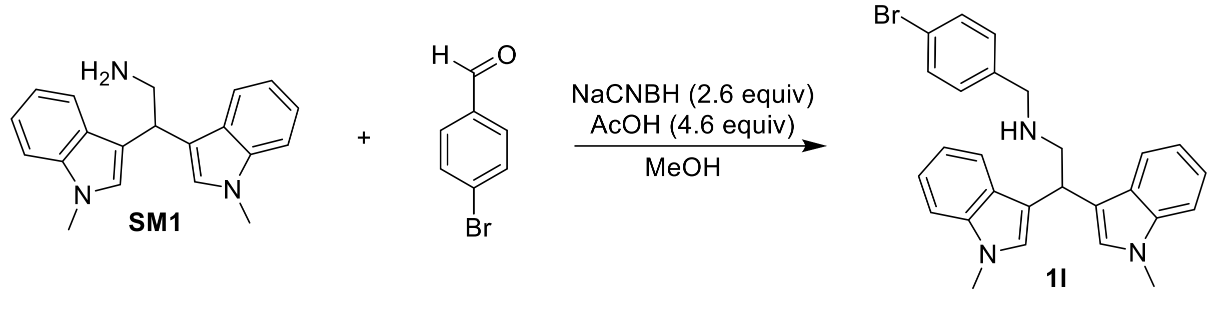


To a stirred solution of bisindole **SM1** (200 mg, 1 equiv) in methanol (13.2 mL, 0.05 M) was added glacial acetic acid (174 µL, 4.6 eq) followed by sodium cyanoborohydride (108 mg, 2.6 eq) under argon atmosphere at 0°C. 4-bromobenzaldehyde (146.5 mg, 1.1 equiv) in methanol (9.9 mL, 0.08 M) was then added. The resulting mixture was stirred at room temperature for 16 h. A 2 M solution of $Na_2CO_3$ (15 mL) was added to adjust the pH to 8–9, and the solvent was removed *in vacuo*. The residue was partitioned between DCM and water, and the organic layer was washed with water (15 mL) and brine (15 mL), dried with anhydrous $Na_2SO_4$, filtered, and concentrated *in vacuo*. The residue was purified with flash chromatography on silica gel (dichloromethane/ammonia, 99/1 to dichloromethane/methanol/ammonia, 99/1/1), obtaining **1l** as a white solid; yield: 41% (129 mg). $R_f$ = 0,27 (silica gel; dichloromethane/methanol/ammonia 95/5/1). $^1$H NMR (400 MHz, $CDCl_3$): $\delta$ = 7.59 (d, $J$ = 8.0 Hz, 2H), 7.38 (d, $J$ = 8.5 Hz, 2H), 7.28-7.26 (m, 2H), 7.20 (ddd, $J_1$ = 1.0 Hz, $J_2$ = 7.0 Hz, $J_3$ = 8.0 Hz, 2H), 7.10 (d, $J$ = 8.5 Hz, 2H), 7.04 (ddd, $J_1$ = 1.0 Hz, $J_2$ = 7.0 Hz, $J_3$ = 8.0 Hz, 2H), 6.85 (d, $J$ = 1.0 Hz, 2H), 4.83 (t, $J$ = 7.5 Hz, 1H), 3.77 (s, 2H), 3.70 (s, 6H), 3.35 (d, $J$ = 7.5 Hz, 2H) ppm. $^{13}$C{$^1$H} NMR (151 MHz, $CDCl_3$): $\delta$ = 139.2, 137.4, 131.5, 130.1, 127.4, 127.0, 121.7, 120.8, 119.7, 118.9, 116.4, 109.4, 53.6, 53.1, 34.4, 32.8 ppm. HRMS (m/z): $[M+H]^+$ calcd for $C_{27}H_{27}BrN_3$ 472.1383; found 472.1380.

The synthesis of bisindole **1o** was carried out by adapting a procedure from the literature.[9]

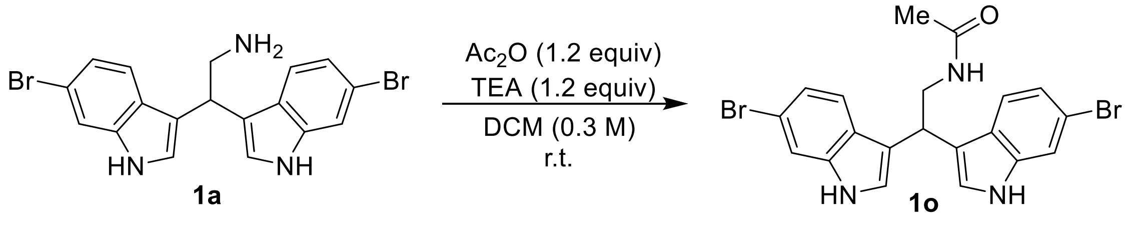


To a stirred suspension of **1a** (0.28 mmol, 120 mg, 1.0 equiv) in DCM (950 µL mL, 0.30 M) at room temperature were added triethylamine (46 µL, 1.2 equiv) and acetic anhydride (32

μL, 1.2 equiv). The reaction mixture was stirred at room temperature for 16 h. The reaction was then quenched by the addition of saturated aqueous $NaHCO_3$ (3 mL), and the layers were separated. The aqueous phase was extracted with DCM (3 × 5 mL). The combined organic layers were washed with brine (10 mL), dried over anhydrous $Na_2SO_4$, filtered, and concentrated under reduced pressure to afford a crude oil. The crude product was purified by flash column chromatography on silica gel using EtOAc as the eluent afforded compound **1o** as a white solid in 90% yield (120 mg). $^1$H NMR (400 MHz, $CD_3OD$): $\delta$ = 10.48 (br s, 2H), 8.06 (br t, $J$ = 5.5 Hz, 1H), 7.49 (d, $J$ = 2.0 Hz, 2H), 7.41 (d, $J$ = 8.5 Hz, 2H), 7.07 (d, $J$ = 0.5 Hz, 2H), 7.03 (dd, $J_1$ = 2.0 Hz, $J_2$ = 8.5 Hz, 2H), 4.71 (dd, $J_1 \approx J_2$ = 7.5 Hz, 1H), 3.85-391 (m, 2 H), 1.85 (s, 3 H) ppm. Spectroscopic data are in accordance with the literature.[5]

## 7. Synthesis of *trans*-resveratrol

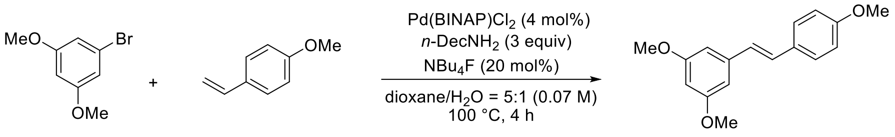


**1p** 1.3 mmol

**2c** 2 equiv

**5pc**, y = 70%

Compound **1p** (1.3 mmol, 280 mg), Pd(BINAP)$Cl_2$ (4 mol%, 41.3 mg), 1-decylamine (3.87 mmol, 774 µL), $NBu_4F \cdot 3H_2O$ (20 mol%, 83.2 mg) and the olefin **2c** (2.58 mmol, 343 µL) were added in a dried 100 mL Schlenk tube, equipped with a magnetic stirring bar. Degassed dioxane (15.5 mL) and degassed water (3.1 mL) were added, and the reaction was vigorously stirred at 100 °C in an oil bath under $N_2$ for 4 h. The nitrogen was removed, the crude was filtered on silica plug (EtOAc) and the product **5pc** has been purified by flash column chromatography on silica gel (cyclohexane/EtOAc, 98/2 to cyclohexane/EtOAc, 95/5), obtaining a colorless oil; yield: 70% (0.91 mmol, 247 mg). $^1$H NMR (600 MHz, $CDCl_3$): $\delta$ = 7.45 (d, $J$ = 8.5 Hz, 2H), 7.04 (d, $J$ = 16.0 Hz, 1H), 6.92-6.88 (m, 3H), 6.65 (d, $J$ = 2.5 Hz, 2H), 6.38 (dd, $J_1 \approx J_2$ = 2.5 Hz, 1H), 3.83 (s, 9H) ppm. $^{13}C\{^1H\}$ NMR (151 MHz, $CDCl_3$): $\delta$ = 161.1, 159.6, 139.9, 130.1, 128.9, 128.0, 126.8, 114.3, 104.5, 99.8, 55.52, 55.48 ppm. Spectroscopic data are in accordance with the literature.[10]

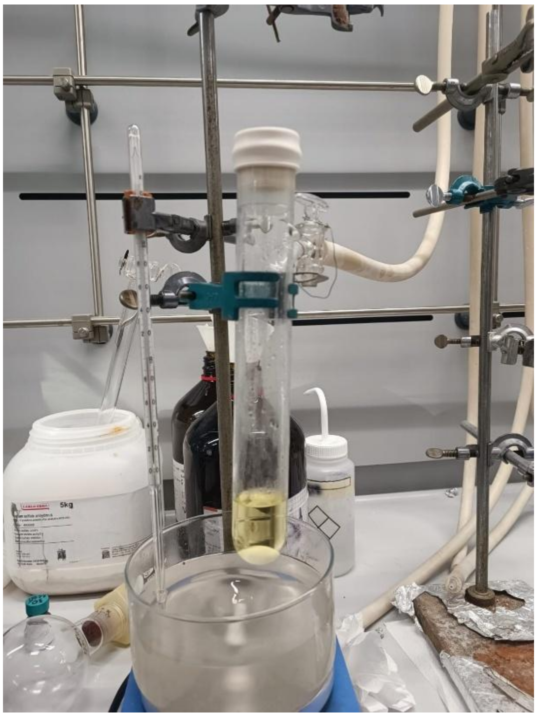

**Figure S4 –** mmol scale synthesis of **5pc** (end of the reaction)

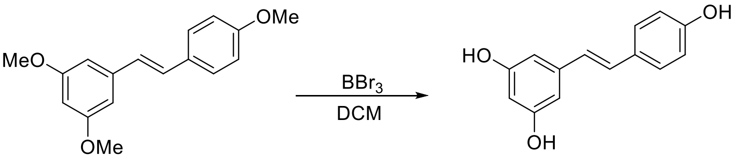


**5pc**

**6**
***trans*-resveratrol**

*Trans*-resveratrol **6** has been realized following a literature procedure.[11] Compound **5pc** (0.91 mmol, 247 mg, 1.0 equiv) was dissolved in DCM (41 mL) and a solution of $BBr_3$ (1.0 M in DCM, 8.19 mmol, 8.19 mL, 9.0 equiv) was added at –78 °C. The mixture was stirred for 1 h at –78 °C. Then the reaction was allowed to warm to room temperature, $H_2O$ (50 mL) was added and the mixture was poured into $H_2O$ (50 mL). Extraction with EtOAc (3 × 50 mL), washing of the combined organic layers with $H_2O$ (50 mL), brine (50 mL), drying over $NaSO_4$ and concentration under reduced pressure gave the crude product, which was purified by flash column chromatography using cyclohexane/EtOAc (90/10) as the eluent. *Trans*-resveratrol **6** was obtained as a white solid; yield: 90 % (0.82 mmol, 186 mg). $^1H$ NMR (600 MHz, DMSO-$d_6$): $\delta$ = 9.52 (s, 1H), 9.16 (s, 2H), 7.38 (d, $J$ = 8.5 Hz, 2H), 6.92 (d, $J$ = 16.5 Hz, 1H), 6.80 (d, $J$ = 16.5 Hz, 1H), 6.75 (d, $J$ = 8.5 Hz, 2H), 6.37 (d, $J$ = 2.0 Hz, 2H), 6.11 (dd, $J_1 \approx J_2$ = 2.0 Hz, 1H) ppm. Spectroscopic data are in accordance with the literature.[11]

## 8. Experimental data for compounds 3 and 5

**2,2-bis(6-((*E*)-styryl)-1*H*-indol-3-yl)ethan-1-amine (3aa)**

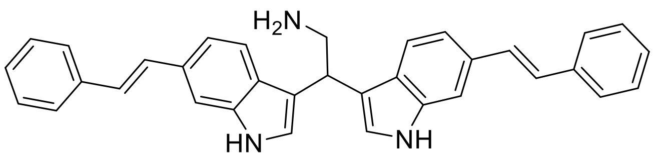


Following the **GP1** (reaction time = 2 h), compound **3aa** has been purified by flash column chromatography on silica gel (dichloromethane/methanol/ammonia, 99/1/1), obtaining a white solid; yield: 81% (39 mg). $R_f$ = 0.38 (silica gel; dichloromethane/methanol/ammonia, 90/10/1). $^1$H NMR (600 MHz, DMSO-$d_6$): $\delta$ = 10.91 (br d, $J$ = 2.5 Hz, 2H), 7.59-7.56 (m, 4H), 7.50-7.47 (m, 4H), 7.36-7.32 (m, 4H), 7.31 (d, $J$ = 16.5 Hz, 2H), 7.26-7.20 (m, 6H), 7.12 (d, $J$ = 16.5 Hz, 2H), 4.39 (t, $J$ = 7.0 Hz, 1H), 3.26 (d, $J$ = 7.0 Hz, 2H) ppm. $^{13}$C{$^1$H} NMR (151 MHz, DMSO-$d_6$): $\delta$ = 137.6, 136.8, 130.1, 130.0, 128.6, 126.9, 126.9, 126.1, 125.4, 123.5, 119.1, 117.4, 116.7, 110.2, 46.8, 37.9 ppm. HRMS (m/z): [M+H]$^+$ calcd for $C_{34}H_{30}N_3$ 480.2434; found 480.2452.

**2,2-bis(5-((*E*)-styryl)-1*H*-indol-3-yl)ethan-1-amine (3ba)**

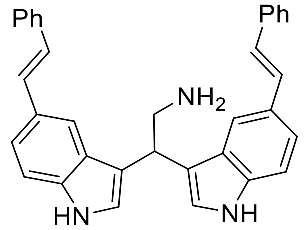


Following the **GP1** (reaction time = 4 h), compound **3ba** has been purified by flash column chromatography on silica gel (dichloromethane/methanol/ammonia, 99/1/1 to dichloromethane/methanol/ammonia, 98/2/1), obtaining a white solid; yield: 86% (41 mg). $R_f$ = 0.20 (silica gel; dichloromethane/methanol/ammonia, 90/10/1). $^1$H NMR (400 MHz, DMSO-$d_6$): $\delta$ = 10.94 (br d, $J$ = 2.5 Hz, 2H), 7.81 (s, 2H), 7.58-7.54 (m, 4H), 7.38 (dd, $J_1$ = 1.5 Hz, $J_2$ = 8.5 Hz, 2H), 7.34-7.30 (m, 6H), 7.28 (dd, $J_1$ = 2.5 Hz, $J_2$ = 8.5 Hz, 4H), 7.21 (d, $J$ = 7.0 Hz, 2H), 7.08 (d, $J$ = 16.5 Hz, 2H), 4.55 (t, $J$ = 7.0 Hz, 1H), 3.39 (d, $J$ = 7.0 Hz, 2H) ppm. $^{13}$C{$^1$H} NMR (101 MHz, DMSO): $\delta$ = 137.7, 136.4, 130.3, 128.6, 127.4, 127.1, 126.8, 126.0, 124.7, 123.2, 119.6, 118.0, 117.1, 111.8, 46.0, 36.6 ppm. HRMS (m/z): [M+H]$^+$ calcd for $C_{34}H_{30}N_3$ 480.2434; found 480.2435.

**2,2-bis(7-((*E*)-styryl)-1*H*-indol-3-yl)ethan-1-amine (3ca)**

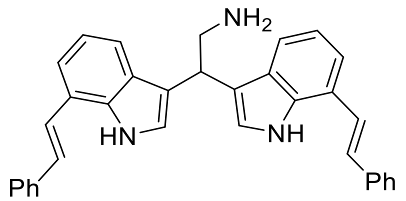

Following the **GP1** (reaction time = 4 h), compound **3ca** has been purified by flash column chromatography on silica gel (dichloromethane/methanol/ammonia, 99/1/1), obtaining a white solid; yield: 75% (36 mg). $R_f$ = 0.34 (silica gel; dichloromethane/methanol/ammonia, 90/10/1). $^1$H NMR (400 MHz, DMSO-$d_6$): $\delta$ = 11.16 (br d, $J$ = 2.5 Hz, 2H), 7.75 (d, $J$ = 16.5 Hz, 2H), 7.70-7.64 (m, 4H), 7.46 (d, $J$ = 8.0 Hz, 2H), 7.44-7.37 (m, 6H), 7.33 (d, $J$ = 2.5 Hz, 2H), 7.31-7.25 (m, 4H), 6.94 (dd, $J_1 \approx J_2$ = 7.5 Hz, 2H), 4.44 (t, $J$ = 7.0 Hz, 1H), 3.30 (d, $J$ = 7.0 Hz, 1H) ppm. $^{13}$C{$^1$H} NMR (101 MHz, DMSO): $\delta$ = 137.6, 134.7, 128.6, 127.8, 127.7, 127.3, 126.4, 124.3, 122.6, 120.8, 118.7, 118.6, 117.4, 117.2, 46.8, 37.9 ppm. HRMS (m/z): [M+K]$^+$ calcd for $C_{34}H_{29}N_3K$ 518.1993; found 518.2001 [M+K]$^+$.

**2,2-bis(6-((*E*)-4-methylstyryl)-1*H*-indol-3-yl)ethan-1-amine (3ab)**

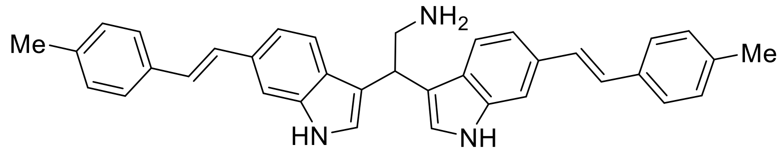


Following the **GP1** (reaction time = 2 h), compound **3ab** has been purified by flash column chromatography on silica gel (dichloromethane/methanol/ammonia, 99/1/1 to dichloromethane/methanol/ammonia, 96/4/1), obtaining a white solid; yield: 79% (40 mg). $R_f$ = 0.30 (silica gel; dichloromethane/methanol/ammonia, 90/10/1). $^1$H NMR (400 MHz, DMSO-$d_6$): $\delta$ = 10.89 (br d, $J$ = 2.5 Hz, 2H), 7.50-7.44 (m, 8H), 7.26-7.19 (m, 6H), 7.17-7.13 (m, 4H), 7.07 (d, $J$ = 16.5 Hz, 2H), 4.39 (t, $J$ = 7.0 Hz, 1H), 3.26 (d, $J$ = 7.0 Hz, 2 H), 2.30 (s, 6H) ppm. $^{13}$C{$^1$H} NMR (101 MHz, DMSO-$d_6$): $\delta$ = 136.8, 136.2, 134.8, 130.2, 129.2, 129.1, 126.7, 126.0, 125.4, 123.4, 119.1, 117.3, 116.7, 110.0, 46.7, 37.8, 20.8 ppm. HRMS (m/z): [M+H]$^+$ calcd for $C_{36}H_{34}N_3$ 508.2747; found 508.2763.

**2,2-bis(6-((*E*)-4-methoxystyryl)-1*H*-indol-3-yl)ethan-1-amine (3ac)**

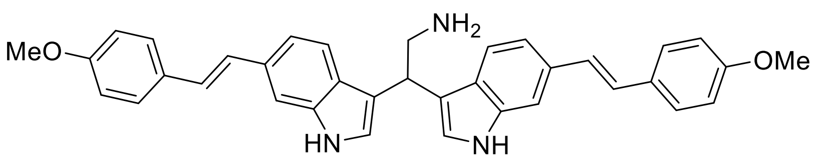


Following the **GP1** (reaction time = 2 h), compound **3ac** has been purified by flash column chromatography on silica gel (dichloromethane/methanol/ammonia, 99/1/1), obtaining a white solid; yield: 72% (39 mg). $R_f$ = 0.36 (silica gel; dichloromethane/methanol/ammonia, 90/10/1). $^1$H NMR (400 MHz, DMSO-$d_6$): $\delta$ = 10.87 (br d, $J$ = 2.5 Hz, 2H), 7.53-7.49 (m, 4H), 7.47 (d, $J$ = 8.5 Hz, 2H), 7.44 (s, 2H), 7.23 (d, $J$ = 2.5 Hz, 2H), 7.20 (dd, $J_1$ = 1.5 Hz, $J_2$ = 8.5 Hz, 2H), 7.14 (d, $J$ = 16.5 Hz, 2H), 7.06 (d, $J$ = 16.5 Hz, 2H), 6.95-6.89 (m, 4H), 4.39 (t, $J$ = 7.0 Hz, 1H), 3.76 (s, 6H), 3.27 (d, $J$ = 7.0 Hz, 2H) ppm. $^{13}$C{$^1$H} NMR (101 MHz, DMSO-$d_6$): $\delta$ = 158.5, 136.9, 130.4, 130.3, 127.8, 127.3, 126.5, 125.2, 123.2, 119.1, 117.2, 116.6, 114.1, 109.8, 55.1, 46.7, 37.7 ppm. HRMS (m/z): [M+H]$^+$ calcd for $C_{36}H_{34}N_3O_2$ 540.2646; found 540.2661.

**4,4'-((1*E*,1'*E*)-((2-aminoethane-1,1-diyl)bis(1*H*-indole-3,6-diyl))bis(ethene-2,1-diyl))dianiline (3ad)**

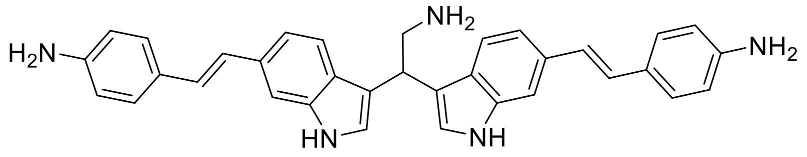

Following the **GP1** (reaction time = 4 h), compound **3ad** has been purified by flash column chromatography on silica gel (dichloromethane/methanol/ammonia, 99/1/1), obtaining a light-yellow solid; yield: 73% (37 mg). $R_f$ = 0.24 (silica gel; dichloromethane/methanol/ammonia, 90/10/1). $^1$H NMR (600 MHz, DMSO-$d_6$): $\delta$ = 10.78 (br d, $J$ = 2.5 Hz, 2H), 7.44 (d, $J$ = 8.5 Hz, 2H), 7.37 (d, $J$ = 1.5 Hz, 2H), 7.26-7.23 (m, 4H), 7.19 (d, $J$ = 2.5 Hz, 2H), 7.14 (dd, $J_1$ = 1.5 Hz, $J_2$ = 8.5 Hz, 2H), 6.94-6.92 (m, 4H), 6.56-6.53 (m, 4H), 5.19 (br s, 4H), 4.37 (t, $J$ = 7.0 Hz, 1H), 3.26 (d, $J$ = 7.0 Hz, 2H) ppm. $^{13}$C{$^1$H} NMR (151 MHz, DMSO-$d_6$): $\delta$ = 148.1, 137.0, 131.0, 127.2, 126.3, 126.1, 125.4, 124.6, 122.8, 119.0, 117.1, 116.4, 113.9, 109.1, 46.6, 37.7 ppm. HRMS (m/z): [M+H]$^+$ calcd for $C_{34}H_{32}N_5$ 510.2653; found 510.2656.

**2,2-bis(6-((*E*)-4-chlorostyryl)-1*H*-indol-3-yl)ethan-1-amine (3ae)**

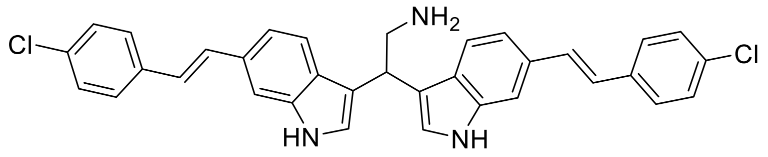

Following the **GP1** (reaction time = 2 h), compound **3ae** has been purified by flash column chromatography on silica gel (dichloromethane/methanol/ammonia, 99/1/1), obtaining a yellow solid; yield: 77% (42 mg). $^1$H NMR (600 MHz, DMSO-$d_6$): $\delta$ = 10.95 (br d, $J$ = 2.5 Hz, 2H), 7.60 (d, $J$ = 8.5 Hz, 2H), 7.51-7.48 (m, 4H), 7.41-7.38 (m, 4H), 7.33 (d, $J$ = 16.0 Hz, 2H), 7.28 (d, $J$ = 2.5 Hz, 2H), 7.24 (dd, $J_1$ = 1.5 Hz, $J_2$ = 8.5 Hz, 2H), 7.11 (d, $J$ = 16.0 Hz, 2H), 4.45 (t, $J$ = 7.0 Hz, 1H), 3.32 (d, $J$ = 7.0 Hz, 2H) ppm. $^{13}$C{$^1$H} NMR (151 MHz, DMSO-$d_6$): $\delta$ = 136.8, 136.6, 131.1, 131.0, 129.8, 128.5, 127.7, 126.9, 124.1, 123.7, 119.1, 116.9, 116.8, 110.4, 46.2, 36.9 ppm. Spectroscopic data are in accordance with the literature.[6]

**2,2-bis(6-((*E*)-4-(trifluoromethyl)styryl)-1*H*-indol-3-yl)ethan-1-amine (3af)**

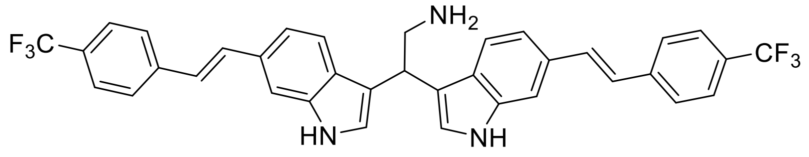

Following the **GP1** (reaction time = 4 h), compound **3af** has been purified by flash column chromatography on silica gel (dichloromethane/methanol/ammonia, 99/1/1 to dichloromethane/methanol/ammonia, 97/3/1), obtaining a white solid; yield: 74% (46 mg). $R_f$ = 0.22 (silica gel; dichloromethane/methanol/ammonia, 90/10/1). $^1$H NMR (400 MHz, DMSO-$d_6$): $\delta$ = 10.99 (br d, $J$ = 2.5 Hz, 2H), 7.81-7.77 (m, 4H), 7.71-7.66 (m, 4H), 7.54 (br s, 2H), 7.52 (d, $J$ = 8.5 Hz, 2H), 7.49 (d, $J$ = 16.5 Hz, 2H), 7.31-7.27 (m, 4H), 7.22 (d, $J$ =

16.5 Hz, 2H), 4.41 (t, *J* = 7.0 Hz, 1H), 3.28 (d, *J* = 7.0 Hz, 2H) ppm. $^{19}$F NMR (376 MHz, DMSO-$d_6$): $\delta$ = -60.69 ppm. $^{13}$C{$^{1}$H} NMR (101 MHz, DMSO): $\delta$ = 141.9 (q, *J* = 1.0 Hz), 136.7, 133.1, 129.5, 127.3, 126.8 (q, *J* = 31.5 Hz), 126.5, 125.5 (q, *J* = 3.5 Hz), 124.5 (q, *J* = 271.5 Hz), 124.0, 123.8, 119.2, 117.4, 116.9, 110.9, 46.7, 37.7 ppm. HRMS (m/z): [M+H]$^+$ calcd for $C_{36}H_{28}F_6N_3$ 616.2182; found 616.2185.

**dimethyl 4,4'-((1*E*,1'*E*)-((2-aminoethane-1,1-diyl)bis(1*H*-indole-3,6-diyl))bis(ethene-2,1-diyl))dibenzoate (3ag)**

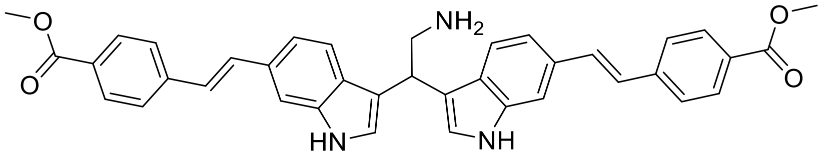


Following the **GP1** (reaction time = 4 h), dichloromethane was added to the reaction mixture, inducing the formation of a crystalline solid, which was collected by filtration, washed with ethanol and dichloromethane, and finally dried to afford compound **3ag**, obtaining a yellow solid; yield: 50% (30 mg). $R_f$ = 0.27 (silica gel; dichloromethane/methanol/ammonia, 90/10/1). $^{1}$H NMR (600 MHz, DMSO-$d_6$): $\delta$ = 11.09 (br d, *J* = 2.5 Hz, 2H), 7.94-7.92 (m, 4H), 7,73-7.70 (m, 4H), 7.56 (s, 2H), 7.53 (d, *J* = 8.5 Hz, 2H), 7.50 (d, *J* = 16.5 Hz, 2H), 7.38 (d, *J* = 2.5 Hz, 2H), 7.31 (dd, $J_1$ = 1.5 Hz, $J_2$ = 8.5 Hz, 2H), 7.22 (d, *J* = 16.5 Hz, 2H), 4.60 (t, *J* = 7.5 Hz, 1H), 3.85 (s, 6H), 3.45 (d, *J* = 7.5 Hz, 2H) ppm. $^{13}$C{$^{1}$H} NMR (151 MHz, DMSO-$d_6$): $\delta$ = 166.5, 143.0, 137.3, 133.6, 130.3, 130.1, 128.0, 127.4, 126.7, 124.9, 124.8, 119.5, 117.6, 116.3, 111.5, 52.5, 45.0, 35.3 ppm. HRMS (m/z): [M+H]$^+$ calcd for $C_{38}H_{34}N_3O_4$ 596.2541; found 596.2540.

**2,2-bis(6-((E)-3-methoxystyryl)-1H-indol-3-yl)ethan-1-amine (3ah)**

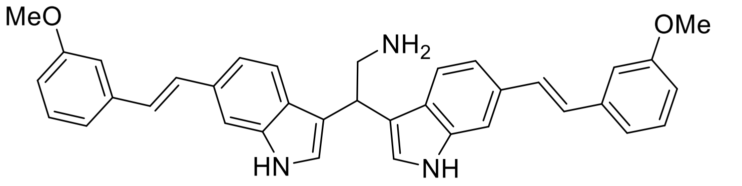


Following the **GP1** (reaction time = 2 h), compound **3ah** has been purified by flash column chromatography on silica gel (dichloromethane/methanol/ammonia, 99/1/1), obtaining a white solid; yield: 67% (36 mg). $R_f$ = 0.28 (silica gel; dichloromethane/methanol/ammonia, 90/10/1). $^{1}$H NMR (400 MHz, DMSO-$d_6$): $\delta$ = 10.92 (br s, 2H), 7.52-7.47 (m, 4H), 7.32 (d, *J* = 16.5 Hz, 2H), 7.29-7.21 (m, 6H), 7.18-7.13 (m, 4H), 7.09 (d, *J* = 16.5 Hz, 2H), 6.79 (dd, $J_1$ = 2.5 Hz, $J_2$ = 8.0 Hz, 2H), 4.39 (t, *J* = 7.0 Hz, 1H), 3.79 (s, 6H), 3.26 (d, *J* = 7.0 Hz, 2H) ppm.$^{13}$C{$^{1}$H} NMR (101 MHz, DMSO-$d_6$): $\delta$ = 159.6, 139.1, 136.8, 130.4, 130.0, 129.6, 126.9, 125.4, 123.5, 119.2, 118.7, 117.3, 116.7, 112.8, 111.1, 110.3, 55.0, 46.8, 37.9 ppm. HRMS (m/z): [M+H]$^+$ calcd for $C_{36}H_{34}N_3O_2$ 540.2646; found 540.2666.

**2,2-bis(6-((*E*)-3-(trifluoromethyl)styryl)-1*H*-indol-3-yl)ethan-1-amine (3ai)**

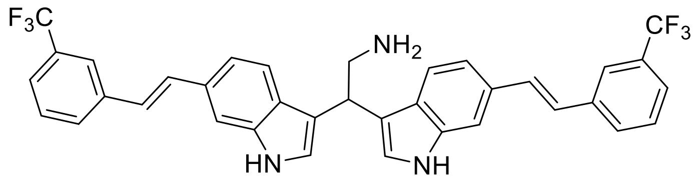

Following the **GP1** (reaction time = 4 h), compound **3ai** has been purified by flash column chromatography on silica gel (dichloromethane/methanol/ammonia, 99/1/1), obtaining a white solid; yield: 65% (40 mg). $R_f$ = 0.23 (silica gel; dichloromethane/methanol/ammonia, 90/10/1). $^1$H NMR (600 MHz, $CDCl_3$): $\delta$ = 8.24 (br s, 2H), 7.71 (s, 2H), 7.61 (d, 2H, $J$ = 7.5 Hz), 7.56 (d, 2H, $J$ = 8.0 Hz), 7.48-7.40 (m, 6H), 7.28 (d, 2H, $J$ = 8.5 Hz), 7.21 (d, 2H, $J$ = 16.5 Hz), 7.05 (d, 2H, $J$ = 16.5 Hz), 7.03 (s, 2H), 4.54 (t, 1H, $J$ = 7.0 Hz), 3.46 (d, 2H, $J$ = 7.0 Hz) ppm. $^{19}$F NMR (565 MHz, $CDCl_3$): $\delta$ = -62.70. $^{13}$C{$^1$H} NMR (151 MHz, $CDCl_3$): $\delta$ = 138.7, 137.2, 131.7, 131.3, 131.2 (q, $J$ = 32.0 Hz), 129.5, 129.2, 127.4, 125.5, 124.4 (q, $J$ = 272.5 Hz), 123.7 (q, $J$ = 4.0 Hz), 123.3, 122.9 (q, $J$ = 4.0 Hz), 119.9, 118.4, 118.1, 110.2, 46.8, 38.2 ppm. HRMS (m/z): $[M+H]^+$ calcd for $C_{36}H_{28}F_6N_3$ 616.2182; found 616.2186.

**2,2-bis(6-((*E*)-3-nitrostyryl)-1*H*-indol-3-yl)ethan-1-amine (3aj)**

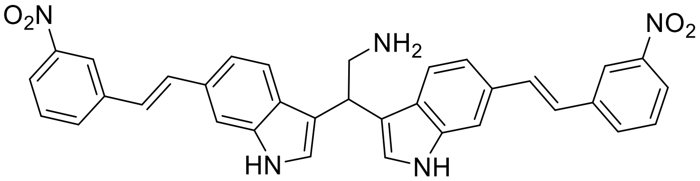

Following the **GP1** (reaction time = 4 h), compound **3aj** has been purified by flash column chromatography on silica gel (dichloromethane/methanol/ammonia, 99/1/1), obtaining a yellow solid; yield: 42% (24 mg). $R_f$ = 0.41 (silica gel; dichloromethane/methanol/ammonia, 90/10/1). $^1$H NMR (600 MHz, DMSO-$d_6$): $\delta$ = 11.00 (br d, 2H, $J$ = 2.5 Hz), 8.40 (t, 2H, $J$ = 2.0 Hz), 8.06-8.03 (m, 4H), 7.63 (dd, 2H, $J_1 \approx J_2$ = 8.0 Hz), 7.56 (d, 2H, $J$ = 1.5 Hz), 7.54 (d, 2H, $J$ = 16.5 Hz), 7.52 (d, 2H, $J$ = 8.5 Hz), 7.31-7.27 (m, 6H), 4.43 (t, 1H, $J$ = 7.0 Hz), 3.29 (d, 2 H, $J$ = 7.0 Hz) ppm.$^{13}$C{$^1$H} NMR (151 MHz, DMSO): $\delta$ = 148.4, 139.7, 136.7, 133.1, 132.1, 130.0, 129.4, 127.3, 123.9, 123.2, 121.2, 120.3, 119.2, 117.3, 117.0, 110.9, 46.7, 37.6 ppm. HRMS (m/z): $[M+H]^+$ calcd for $C_{34}H_{28}N_5O_4$ 570.2133; found 570.2136.

**2,2-bis(6-((*E*)-2-methylstyryl)-1*H*-indol-3-yl)ethan-1-amine (3ak)**

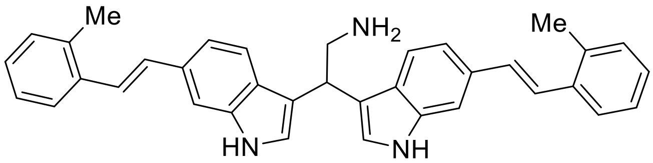

Following the **GP1** (reaction time = 4 h), compound **3ak** has been purified by flash column chromatography on silica gel (dichloromethane/methanol/ammonia, 99/1/1 to dichloromethane/methanol/ammonia, 98/2/1), obtaining a white solid; yield: 71% (36 mg). $R_f$ = 0.32 (silica gel; dichloromethane/methanol/ammonia, 90/10/1). $^1$H NMR (600 MHz, $CDCl_3$): $\delta$ = 8.12 (br s, 2H), 7.60 (d, 2H, $J$ = 7.5 Hz), 7.57 (d, 2H, $J$ = 8.5), 7.44 (s, 2H), 7.31 (d, 2H, $J$ = 16.0 Hz), 7.31 (dd, 2H, $J_1$ = 1.5 Hz, $J_2$ = 8.5 Hz), 7.22-7.15 (m, 6H), 7.09 (d, 2H,

$J$ = 16.0 Hz), 7.02 (d, 2H, $J$ = 2.0 Hz), 4.55 (t, 1H, $J$ = 7.0 Hz), 3.48 (t, 2H, $J$ = 7.0 Hz), 2.43 (s, 6H) ppm. $^{13}C\{^1H\}$ NMR (151 MHz, $CDCl_3$): $\delta$ = 137.2, 137.0, 135.7, 132.3, 131.2, 130.5, 127.3, 127.1, 126.3, 125.3, 125.0, 123.0, 119.8, 118.3, 118.2, 109.9, 46.9, 38.4, 20.1 ppm. HRMS (m/z): $[M+H]^+$ calcd for $C_{36}H_{34}N_3$ 508.2747; found 508.2765.

**2,2-bis(6-((*E*)-2-methoxystyryl)-1*H*-indol-3-yl)ethan-1-amine (3al)**

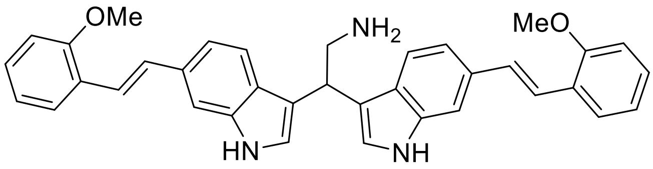


Following the **GP1** (reaction time = 2 h), compound **3al** has been purified by flash column chromatography on silica gel (dichloromethane/methanol/ammonia, 99/1/1 to dichloromethane/methanol/ammonia, 97/3/1), obtaining a white solid; yield: 74% (40 mg). $R_f$ = 0.28 (silica gel; dichloromethane/methanol/ammonia, 90/10/1). $^1H$ NMR (400 MHz, $CDCl_3$): $\delta$ = 8.06 (br s, 2H), 7.61 (dd, 2H, $J_1$ = 1.5 Hz, $J_2$ = 7.5 Hz), 7.56 (d, 2H, $J$ = 8.5 Hz), 7.50-7.44 (m, 4H), 7.32 (dd, 2H, $J_1$ = 1.5 Hz, $J_2$ = 8.5 Hz), 7.25-7.17 (m, 4H), 7.02-6.94 (m, 4H), 6.90 (dd, 2H, $J_1$ = 1.5 Hz, $J_2$ = 8.5 Hz), 4.53 (t, 1H, $J$ = 7.0 Hz), 3.89 (s, 6H), 3.45 (d, 2H, $J$ = 7.0 Hz) ppm. $^{13}C\{^1H\}$ NMR (101 MHz, $CDCl_3$): $\delta$ = 156.9, 137.2, 132.6, 130.3, 128.3, 127.1, 127.0, 126.3, 122.9, 121.7, 120.9, 119.7, 118.6, 118.1, 111.1, 109.7, 55.7, 46.9, 38.4 ppm. HRMS (m/z): $[M+H]^+$ calcd for $C_{36}H_{34}N_3O_2$ 540.2646; found 540.2644.

**2,2-bis(6-((*E*)-2-(naphthalen-2-yl)vinyl)-1*H*-indol-3-yl)ethan-1-amine (3am)**

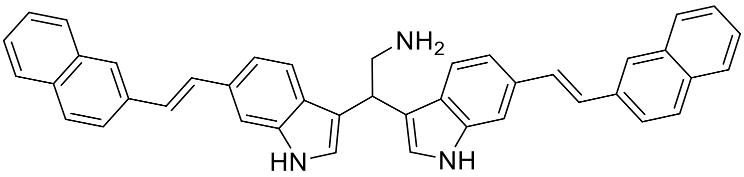


Following the **GP1** (reaction time = 18 h), compound **3am** has been purified by flash column chromatography on silica gel (dichloromethane/methanol/ammonia, 99/1/1) and further purified by preparative chromatography (dichloromethane/methanol/ammonia, 90/10/1), obtaining an off-white solid; yield: 53% (31 mg). $R_f$ = 0.31 (silica gel; dichloromethane/methanol/ammonia, 90/10/1). $^1H$ NMR (600 MHz, DMSO-$d_6$): $\delta$ = 11.04 (br d, $J$ = 2.5 Hz, 2H), 7.98 (d, $J$ = 1.5 Hz, 2H), 7.89-7.86 (m, 9H), 7.57 (d, $J$ = 1.5 Hz, 2H), 7.54 (d, $J$ = 8.5 Hz, 2H), 7.51-7.48 (m, 3H), 7.47-7.44 (m, 4H), 7.35 (d, $J$ = 2.5 Hz, 2H), 7.31 (d, $J$ = 16.5 Hz, 2H), 4.57 (t, $J$ = 7.5 Hz, 1H), 3.43 (d, $J$ = 7.5 Hz, 2H) ppm. $^{13}C\{^1H\}$ NMR (151 MHz, DMSO-$d_6$): $\delta$ = 136.9, 135.2, 133.4, 132.3, 130.7, 130.3, 128.1, 127.7, 127.6, 126.7, 126.4, 125.64, 125.60, 125.58, 123.9, 123.6, 119.1, 116.9, 116.1, 110.4, 45.0, 35.5 ppm. HRMS (m/z): $[M+H]^+$ calcd for $C_{42}H_{34}N_3$ 580.2747; found 580.2776.

**2,2-bis(6-((*E*)-2-(thiophen-2-yl)vinyl)-1*H*-indol-3-yl)ethan-1-amine (3an)**

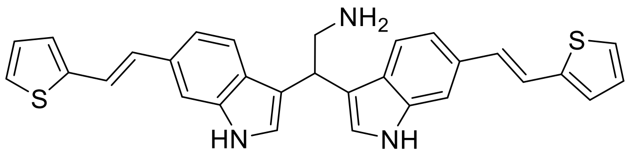

Following the **GP1** (reaction time = 18 h), compound **3an** has been purified by flash column chromatography on silica gel (dichloromethane/ammonia, 99/1 to dichloromethane/methanol/ammonia, 96/4/1), obtaining a white solid; yield: 59% (29 mg). $R_f$ = 0.29 (silica gel; dichloromethane/methanol/ammonia, 90/10/1). $^1$H NMR (400 MHz, DMSO-$d_6$): $\delta$ = 10.92 (br d, $J$ = 2.5 Hz, 2H), 7.47 (d, $J$ = 8.5 Hz, 2H), 7.44 (s, 2H), 7.39 (dd, $J_1$ = 1.0, $J_2$ = 5.0 Hz, 2H), 7.31 (d, $J$ = 16.0 Hz, 2H), 7.25 (d, $J$ = 2.5 Hz, 2H), 7.19 (dd, $J_1$ = 1.5 Hz, $J_2$ = 8.5 Hz, 2H), 7.16 (d, $J$ = 3.0 Hz, 2H), 7.04-7.02 (m, 2H), 6.99 (d, $J$ = 16.0 Hz, 2H), 4.38 (t, $J$ = 7.0 Hz, 1H), 3.26 (d, $J$ = 7.0 Hz, 2H) ppm. $^{13}$C{$^1$H} NMR (101 MHz, DMSO-$d_6$): $\delta$ = 143.0, 136.8, 129.62, 129.56, 127.9, 126.9, 125.6, 124.3, 123.6, 119.2, 119.1, 117.3, 116.6, 110.0, 46.7, 37.7 ppm. HRMS (m/z): [M+H]$^+$ calcd for $C_{30}H_{26}N_3S_2$ 492.1563; found 492.1583.

**2,2-bis(6-((*E*)-2-(pyridin-4-yl)vinyl)-1*H*-indol-3-yl)ethan-1-amine (3ao)**

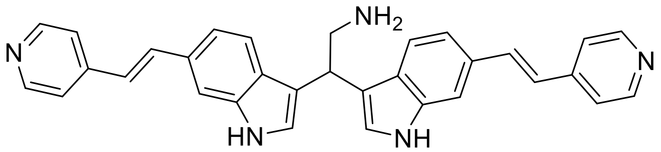

Following the **GP1** (reaction time = 3 h), compound **3ao** has been purified by flash column chromatography on silica gel (dichloromethane/methanol/ammonia, 98/2/1 to dichloromethane/methanol/ammonia, 82/18/1), obtaining a yellow solid; yield: 94% (45mg). $R_f$ = 0.15 (silica gel; dichloromethane/methanol/ammonia, 90/10/1). $^1$H NMR (400 MHz, DMSO-$d_6$): $\delta$ = 11.17 (br d, $J$ = 2.5 Hz, 2H), 8.52-8.47 (m, 4H), 7.62 (d, $J$ = 16.5 Hz, 2H), 7.59-7.55 (m, 3H), 7.55-7.52 (m, 5H), 7.42 (d, $J$ = 2.5 Hz, 2H), 7.33 (dd, $J_1$ = 1.5 Hz, $J_2$ = 8.5 Hz, 2H), 7.12 (d, $J$ = 16.5 Hz, 2H), 4.69 (t, $J$ = 7.5 Hz, 1H), 3.51 (d, $J$ = 7.5 Hz, 2H) ppm.$^{13}$C{$^1$H} NMR (101 MHz, DMSO): $\delta$ = 149.9, 144.8, 136.8, 134.7, 129.4, 127.0, 124.7, 123.1, 120.6, 119.0, 117.3, 115.2, 111.4, 43.7, 33.5 ppm. HRMS (m/z): [M+H]$^+$ calcd for $C_{32}H_{28}N_5$ 482.2339; found 482.2334.

**di-*tert*-butyl 3,3'-((2-aminoethane-1,1-diyl)bis(1*H*-indole-3,6-diyl))(2*E*,2'*E*)-diacrylate (3ap)**

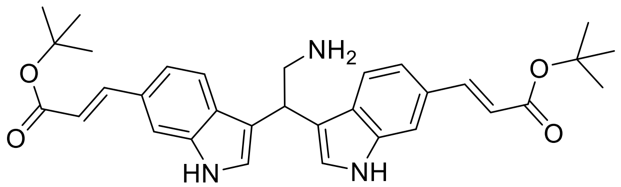

Following the **GP1** (reaction time = 4 h), compound **3ap** has been purified by flash column chromatography on silica gel (dichloromethane/methanol/ammonia, 99/1/1), and further purified by preparative chromatography (dichloromethane/methanol/ammonia, 90/10/1), obtaining a white solid; yield: 43% (23 mg). $R_f$ = 0.26 (silica gel;

dichloromethane/methanol/ammonia, 90/10/1). $^{1}H$ NMR (600 MHz, DMSO-$d_6$): $\delta$ = 11.09 (br d, $J$ = 2.5 Hz, 2H), 7.60 (d, $J$ = 16.0 Hz, 2H), 7.56 (d, $J$ = 1.5 Hz, 2H), 7.47 (d, $J$ = 8.5 Hz, 2H), 7.36 (d, $J$ = 2.5 Hz, 2H), 7.25 (dd, $J_1$ = 1.5 Hz, $J_2$ = 8.5 Hz, 2H), 6.35 (d, $J$ = 16.0 Hz, 2H), 4.39 (t, $J$ = 7.0 Hz, 1H), 3.25 (d, $J$ = 7.0 Hz, 2H), 1.47 (s, 18H) ppm. $^{13}C\{^{1}H\}$ NMR (151 MHz, DMSO-$d_6$): $\delta$ = 166.0, 145.5, 136.4, 128.6, 126.9, 125.1, 119.3, 117.6, 117.5, 116.4, 112.9, 79.5, 46.7, 37.6, 27.9 ppm. HRMS (m/z): $[M+H]^+$ calcd for $C_{32}H_{38}N_3O_4$ 528.2856; found 528.2857.

**(*E*)-6-styryl-1*H*-indole (5ea)**

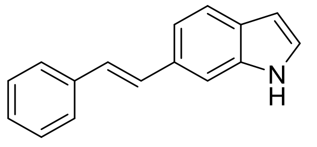

From 6-bromoindole: Following the **GP2** (reaction time = 18 h), using 1-decylamine, compound **5ea** has been purified by flash column chromatography on silica gel (cyclohexane/ EtOAc, 95/5), obtaining a white solid; yield: 85% (37 mg).

From 6-iodioindole: Following the **GP2** (reaction time = 18 h), using 1-decylamine, compound **5ea** has been purified by flash column chromatography on silica gel (cyclohexane/ EtOAc, 95/5), obtaining a white solid; yield: 70% (31 mg).

From 6-indolyl triflate: Following the **GP2** (reaction time = 18 h), using 1-decylamine, compound **5ea** has been purified by flash column chromatography on silica gel (cyclohexane/ EtOAc, 95/5), obtaining a white solid; yield: 20% (9 mg).

$^{1}H$ NMR (600 MHz, $CDCl_3$): $\delta$ = 8.16 (br s, 1H), 7.62 (d, $J$ = 8.0 Hz, 1H), 7.55-7.50 (m, 3H), 7.39-7.34 (m, 3H), 7.25-7.21 (m, 3H), 7.13 (d, $J$ = 16.5 Hz, 1H), 6.55 (ddd, $J_1$ = 1.0 Hz, $J_2$ = 2.0 Hz, $J_3$ = 3.0 Hz, 1H) ppm. $^{13}C\{^{1}H\}$ NMR (151 MHz, $CDCl_3$): $\delta$ = 138.0, 136.4, 131.9, 130.0, 128.8, 128.0, 127.3, 127.0, 126.4, 125.1, 121.0, 118.9, 109.6, 103.1 ppm. Spectroscopic data are in accordance with the literature.[12]

**(*E*)-3-styrylpyridine (5fa)**

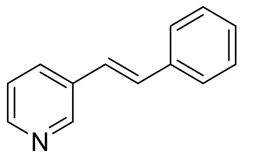

Following the **GP2** (reaction time = 2 h), using CsF, compound **5fa** has been purified by flash column chromatography on silica gel (cyclohexane/EtOAc, 80/20), obtaining a white solid; yield: 91% (33 mg). $^{1}H$ NMR (400 MHz, $CDCl_3$): $\delta$ = 8.72 (d, $J$ = 2.5 Hz, 1H), 8.48 (dd, $J_1$ = 1.5 Hz, $J_2$ = 5.0 Hz, 1H), 7.82 (dt, $J_1$ = 2.0 Hz, $J_2$ = 8.0 Hz, 1H), 7.55-7.50 (m, 2H), 7.40-7.35 (m, 2H), 7.32-7.25 (m, 2H), 7.16 (d, $J$ = 16.5 Hz, 1H), 7.06 (d, $J$ = 16.5, 1H) ppm. $^{13}C\{^{1}H\}$ NMR (101 MHz, $CDCl_3$): $\delta$ = 148.7 (2C), 136.7, 133.1, 132.7, 130.9, 128.9, 128.3, 126.8, 125.0, 123.6 ppm. Spectroscopic data are in accordance with the literature.[13]

**(*E*)-5-styrylquinoline (5ga)**

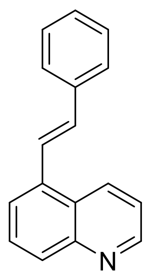

Following the **GP2** (reaction time = 18 h), using CsF, compound **5ga** has been purified by flash column chromatography on silica gel (cyclohexane/EtOAc 90/10 to cyclohexane/EtOAc, 85/15), obtaining a white solid; yield: 48% (22 mg). $^{1}$H NMR (400 MHz, $CDCl_3$): $\delta$ = 8.95 (d, $J$ = 5.5 Hz, 1H), 8.57 (d, $J$ = 8.5 Hz, 1H), 8.08 (d, $J$ = 8.5 Hz, 1H), 7.83-7.71 (m, 3H), 7.63-7.59 (m, 2H), 7.47-7.40 (m, 3H), 7.34 (d, $J$ = 7.5 Hz, 1H), 7.19 (d, $J$ = 16.0 Hz, 1H) ppm. $^{13}$C{$^{1}$H} NMR (101 MHz, $CDCl_3$): $\delta$ = 150.4, 148.7, 137.3, 135.5, 133.0, 132.3, 129.4, 129.4, 129.0, 128.3, 126.9, 126.6, 124.3, 124.0, 121.1 ppm. Spectroscopic data are in accordance with the literature.[14]

**(*E*)-4-styrylisoquinoline (5ha)**

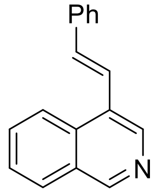


Following the **GP2** (reaction time = 18 h), using CsF, compound **5ha** has been purified by flash column chromatography on silica gel (cyclohexane/EtOAc, 95/5 to cyclohexane/EtOAc, 90/10), obtaining a white solid; yield: 71% (33 mg). $^{1}$H NMR (400 MHz, $CDCl_3$): $\delta$ = 9.17 (s, 1H), 8.76 (s, 1H), 8.15 (dd, $J_1$ = 1.0 Hz, $J_2$ = 8.5 Hz, 1H), 7.98 (d, $J$ = 8.0 Hz, 1H), 7.74 (ddd, $J_1$ = 1.0 Hz, $J_2$ = 7.0 Hz, $J_3$ = 8.5 Hz, 1H), 7.69 (d, $J$ = 16.0 Hz, 1H), 7.64-7.58 (m, 3H), 7.45-7.39 (m, 2H), 7.36- 7.30 (m, 1H), 7.20 (d, $J$ = 16.0 Hz, 1H) ppm. $^{13}$C{$^{1}$H} NMR (101 MHz, $CDCl_3$): $\delta$ = 152.0, 140.6, 137.2, 133.9, 133.3, 130.6, 128.9, 128.7, 128.3, 128.3, 128.2, 127.3, 126.9, 123.0, 122.6 ppm. Spectroscopic data are in accordance with the literature.[15]

**(*E*)-3-styrylaniline (5ia)**

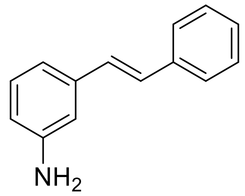


Following the **GP2** (reaction time = 4 h), using 1-decylamine, compound **5ia** has been purified by flash column chromatography on silica gel (cyclohexane/EtOAc, 80/20), obtaining a white solid; yield: 93% (36 mg). $^{1}$H NMR (400 MHz, $CDCl_3$): $\delta$ = 7.52-7.48 (m,

2H), 7.38-7.33 (m, 2H), 7.28-7.23 (m, 2H), 7.16 (dd, $J_1 \approx J_2$ = 8.0 Hz, 1H), 7.08 (d, $J$ = 16.5 Hz, 1H), 7.02 (d, $J$ = 16.5 Hz, 1H), 6.94 (d, $J$ = 7.5 Hz, 1H), 6.86 (dd, $J_1 \approx J_2$ = 2.0 Hz, 1H), 6.61 (ddd, $J_1$ = 1.0 Hz, $J_2$ = 2.0 Hz, $J_3$ = 8.0 Hz, 1H), 3.69 (br s, 2H) ppm. $^{13}C\{^1H\}$ NMR (101 MHz, $CDCl_3$): $\delta$ = 146.8, 138.6, 137.6, 129.7, 129.0, 128.8, 128.7, 127.7, 126.6, 117.5, 114.8, 113.0 ppm. Spectroscopic data are in accordance with the literature.[16]

**(*E*)-4-styrylbenzamide (5ja)**

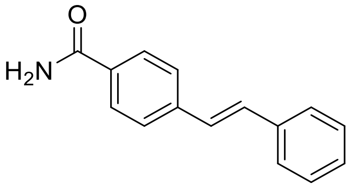


Following the **GP2** (reaction time = 4 h), using 1-decylamine, the resulting precipitate was collected by filtration and washed with diethyl ether to afford compound **5ja**, obtaining a white crystal; yield: 55% (25 mg). $^1H$ NMR (600 MHz, DMSO-$d_6$): $\delta$ = 7.96 (s, 1H), 7.89 (d, $J$ = 8.5 Hz, 2H), 7.68 (d, $J$ = 8.5 Hz, 2H), 7.63 (d, $J$ = 7.5 Hz, 2H), 7.42-7.28 (m, 6H) ppm. $^{13}C\{^1H\}$ NMR (151 MHz, DMSO-$d_6$): $\delta$ = 167.5, 139.8, 136.7, 133.0, 130.1, 128.7, 128.0, 127.9, 127.6, 126.7, 126.2 ppm. Spectroscopic data are in accordance with the literature.[17]

**(*E*)-2-(6-styryl-1*H*-indol-3-yl)ethan-1-amine (5ka)**

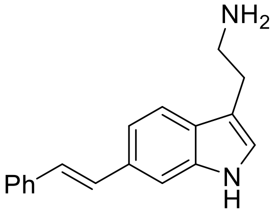


Following the **GP2** (reaction time = 18 h), using 1-decylamine, compound **5ka** has been purified by flash column chromatography on silica gel (dichloromethane/methanol/ammonia, 99/1/1 to dichloromethane/methanol/ammonia, 95/5/1), obtaining a white solid; yield: 60% (32 mg). $R_f$ = 0.29 (silica gel; dichloromethane/methanol/ammonia, 90/10/1). $^1H$ NMR (400 MHz, DMSO-$d_6$): $\delta$ = 10.98 (br d, $J$ = 2.5 Hz, 1H), 7.62-7.58 (m, 2H), 7.55-7.51 (m, 2H), 7.39-7.31 (m, 4H), 7.26-7.21 (m, 2H), 7.17 (d, $J$ = 16.5 Hz, 1H), 2.98 (t, $J$ = 7.0 Hz, 2H), 2.89 (t, $J$ = 7.0 Hz, 2H) ppm. $^{13}C\{^1H\}$ NMR (101 MHz, DMSO-$d_6$): 137.6, 136.6, 130.4, 130.0, 128.6, 127.0 (2C), 126.1, 125.7, 124.2, 118.4, 117.0, 111.1, 110.4, 40.6, 25.5 ppm. HRMS (m/z): $[M+H]^+$ calcd for $C_{18}H_{19}N_2$ 263.1543; found 263.1553.

**(*E*)-2,2-bis(1-methyl-1*H*-indol-3-yl)-*N*-(4-styrylbenzyl)ethan-1-amine (5la)**

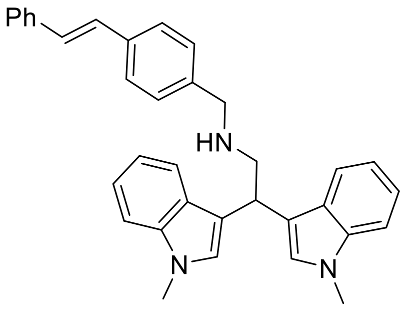

Following the **GP2** (reaction time = 18 h), using 1-decylamine, compound **5la** has been purified by flash column chromatography on silica gel (dichloromethane/methanol/ammonia, 99/1/1), obtaining an off white solid; yield: 77% (38 mg). $R_f$ = 0.30 (silica gel; dichloromethane/methanol/ammonia, 95/10/1). $^1$H NMR (600 MHz, $CDCl_3$): $\delta$ = 7.62 (d, $J$ = 8.0 Hz, 2H), 7.51 (d, $J$ = 8.0 Hz, 2H), 7.43 (d, $J$ = 8.0 Hz, 2H), 7.36 (dd, $J_1 \approx J_2$ = 8.0 Hz, 2H), 7.28-7.27 (m, 3H), 7.23-7.18 (m, 4H), 7.09 (s, 2H), 7.05 (dd, $J_1 \approx J_2$ = 8.0 Hz, 2H), 6.85 (s, 2H), 4.82 (t, $J$ = 7.0 Hz, 1H), 3.85 (s, 2H), 3.70 (s, 6H), 3.39 (d, $J$ = 7.0 Hz, 2H) ppm. $^{13}$C{$^1$H} NMR (151 MHz, $CDCl_3$): $\delta$ = 140.2, 137.6, 137.5, 136.1, 128.8, 128.7 (2C), 128.4, 127.7, 127.6, 126.9, 126.6 (2C), 121.6, 119.8, 118.8, 116.7, 109.3, 53.9, 53.7, 34.5, 32.8 ppm. HRMS (m/z): [M+H]$^+$ calcd for $C_{35}H_{34}N_3$ 496.2747; found 496.2747.

**(*E*)-2-styrylthiophene (5ma)**

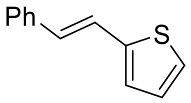

Following the **GP2** (reaction time = 2 h), using CsF, compound **5ma** has been purified by flash column chromatography on silica gel (cyclohexane/EtOAc, 90/10), obtaining a yellowish solid; yield: 40% (15 mg). $^1$H NMR (400 MHz, $CDCl_3$): $\delta$ = 7.49-7.46 (m, 2H), 7.38-7.33 (m, 2H), 7.28-7.23 (m, 2H), 7.20 (d, $J$ = 5.0 Hz, 1H), 7.08 (d, $J$ = 3.5 Hz, 1H), 7.01 (dd, $J_1$ = 3.5 Hz, $J_2$ = 5.0 Hz, 1H), 6.94 (d, $J$ = 16.0 Hz, 1H) ppm. $^{13}$C{$^1$H} NMR (101 MHz, $CDCl_3$): $\delta$ = 143.0, 137.1, 128.8, 128.5, 127.7, 126.4, 126.2, 124.5, 121.9 ppm. Spectroscopic data are in accordance with the literature.[18]

**(E)-2-(3-styrylphenyl)ethan-1-amine (5na)**

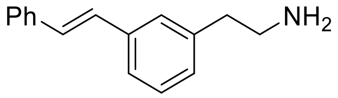

Following the **GP2** (reaction time = 4 h), using CsF, compound **5na** has been purified by flash column chromatography on silica gel (dichloromethane/methanol/ammonia, 98/2/1), obtaining a yellowish solid; yield: 94% (42 mg). $R_f$ = 0.28 (silica gel; dichloromethane/methanol/ammonia, 90/10/1). $^1$H NMR (400 MHz, DMSO-$d_6$) δ 7.65 – 7.57 (m, 2H), 7.42 (d, $J$ = 8.9 Hz, 2H), 7.40 – 7.35 (m, 2H), 7.32 – 7.25 (m, 2H), 7.23 (s, 2H), 7.15 – 7.08 (m, 1H), 2.97 (br s, 2H), 2.82 (t, $J$ = 7.0 Hz, 2H), 2.67 (t, $J$ = 7.0 Hz, 2H). $^{13}$C{$^1$H} NMR (101 MHz, DMSO-$d_6$): $\delta$ = δ 140.8, 137.1, 136.9, 128.7, 128.6, 128.5, 128.2, 128.1,

127.6, 126.8, 126.4, 124.1, 43.5, 40.2 ppm. HRMS (m/z): $[M+H]^+$ calcd for $C_{16}H_{18}N$ 224.1439; found 224.1438.

***N*-(2,2-bis(6-((*E*)-styryl)-1*H*-indol-3-yl)ethyl)acetamide (3oa)**

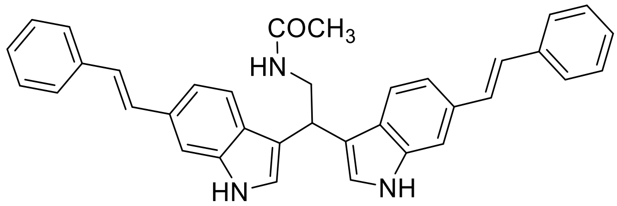


Following the **GP2** (reaction time = 18 h), using 1-decylamine, compound **3oa** has been purified by preparative chromatography (dichloromethane/methanol/ammonia, 90/10/1), obtaining white solid; yield: 60% (31 mg). $R_f$ = 0.28 (silica gel; dichloromethane/methanol/ammonia, 90/10/1). $^1H$ NMR (400 MHz, DMSO-$d_6$): $\delta$ = 10.91 (br s, 2H), 7.92 (br t, $J$ = 5.5 Hz,1H), 7.58 (d, $J$ = 8.0 Hz, 4H), 7.50-7.47 (m, 4H), 7.38-7.34 (m, 4H), 7.31 (d, $J$ = 16.0 Hz, 2H), 7.27-7.19 (m, 6H), 7.12 (d, $J$ = 16.0 Hz, 2H), 4.61 (t, $J$ = 7.0 Hz, 1H), 3.77 (dd, $J_1$ = 5.5 Hz, $J_2$ = 7.0 Hz, 2H), 1.76 (s, 3H) ppm. $^{13}C\{^1H\}$ NMR (101 MHz, DMSO-$d_6$): $\delta$ = 169.2, 137.6, 136.8, 130.1 (2C), 128.6, 126.9, 126.8, 126.1, 125.5, 123.5, 119.1, 116.9, 116.8, 110.3, 43.8, 33.8, 22.7 ppm. HRMS (m/z): $[M+Na]^+$ calcd for $C_{36}H_{31}N_3ONa$ 544.2359; found 544.2364.

**(*E*)-1,3-dimethoxy-5-(4-methoxystyryl)benzene (5pc)**

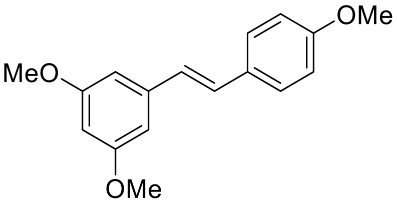


Following the **GP2** (reaction time = 4 h), using 1-decylamine, compound **5pc** has been purified by flash column chromatography on silica gel (cyclohexane/EtOAc, 98/2 to cyclohexane/EtOAc, 95/5), obtaining a colorless oil; yield: 70% (252 mg). $^1H$ NMR (600 CDCl$_3$): $\delta$ = 7.45 (d, $J$ = 8.5 Hz, 2H), 7.04 (d, $J$ = 16.0 Hz, 1H), 6.92-6.88 (m, 3H), 6.65 (d, $J$ = 2.5 Hz, 2H), 6.38 (dd, $J_1 \approx J_2$ = 2.5 Hz, 1H), 3.83 (s, 9H) ppm. $^{13}C\{^1H\}$ NMR (151 MHz, CDCl$_3$): δ = 161.1, 159.6, 139.9, 130.1, 128.9, 128.0, 126.8, 114.3, 104.5, 99.8, 55.5, 55.5 ppm. Spectroscopic data are in accordance with the literature.[10]

# 9. NMR Spectra

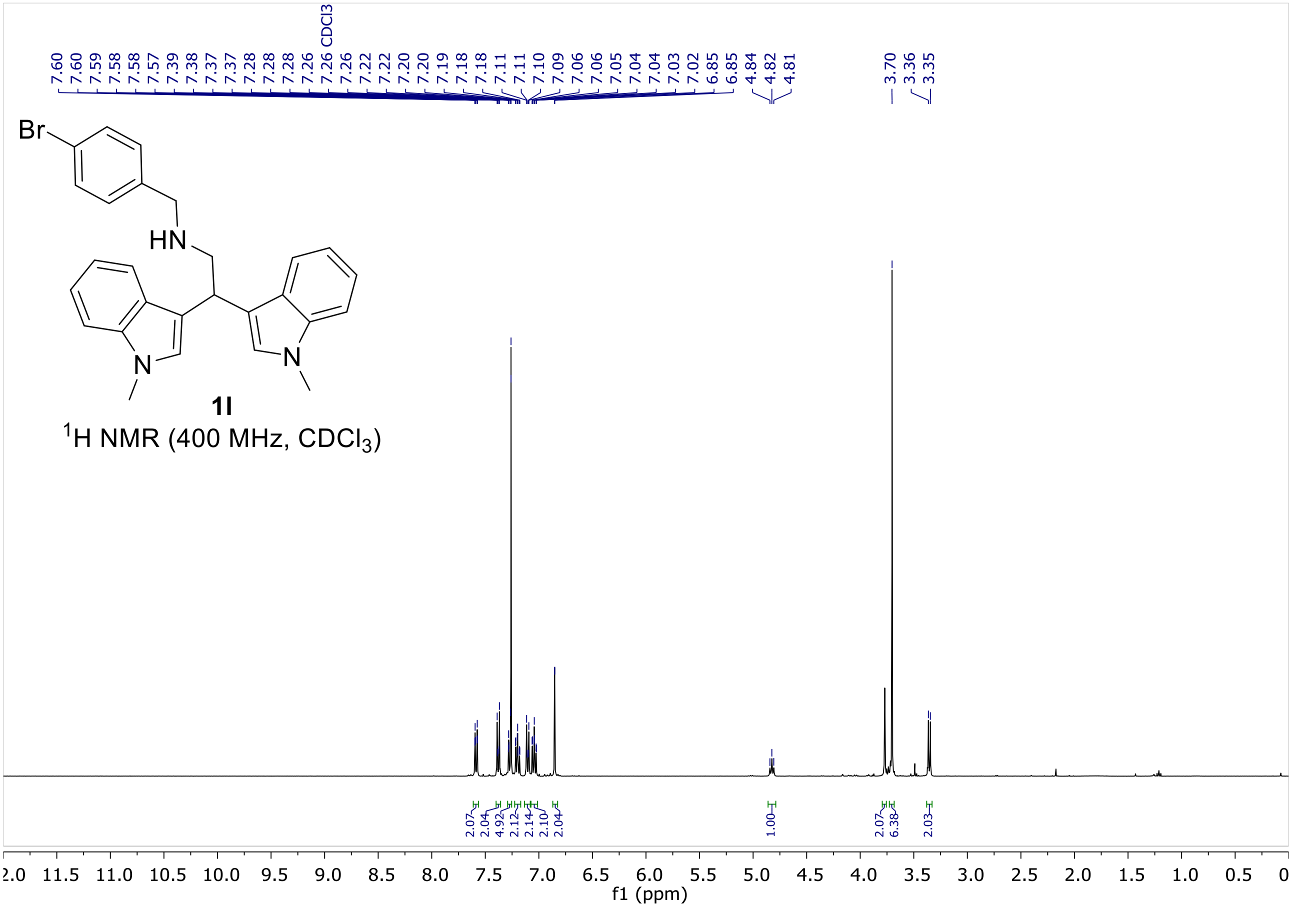


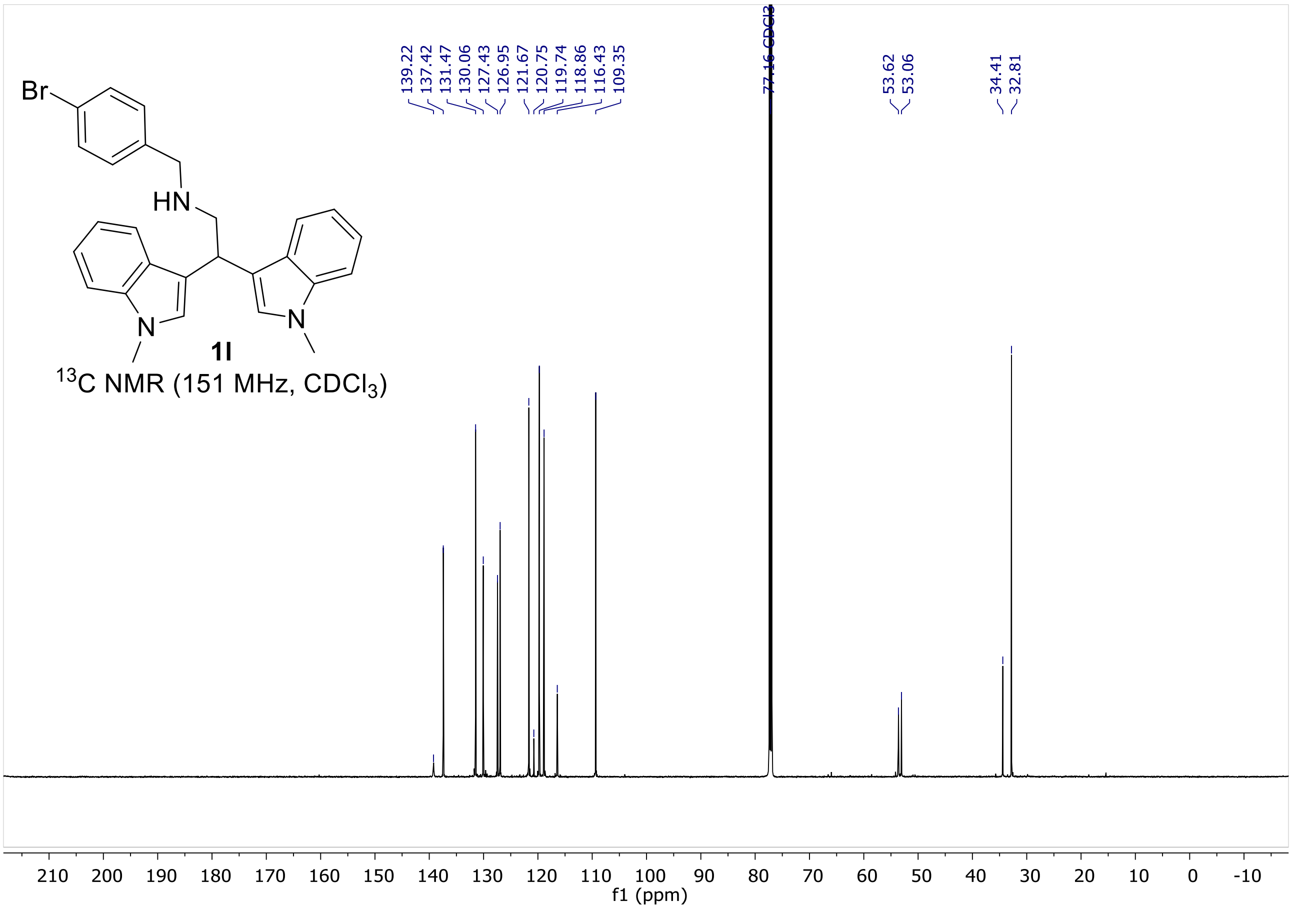

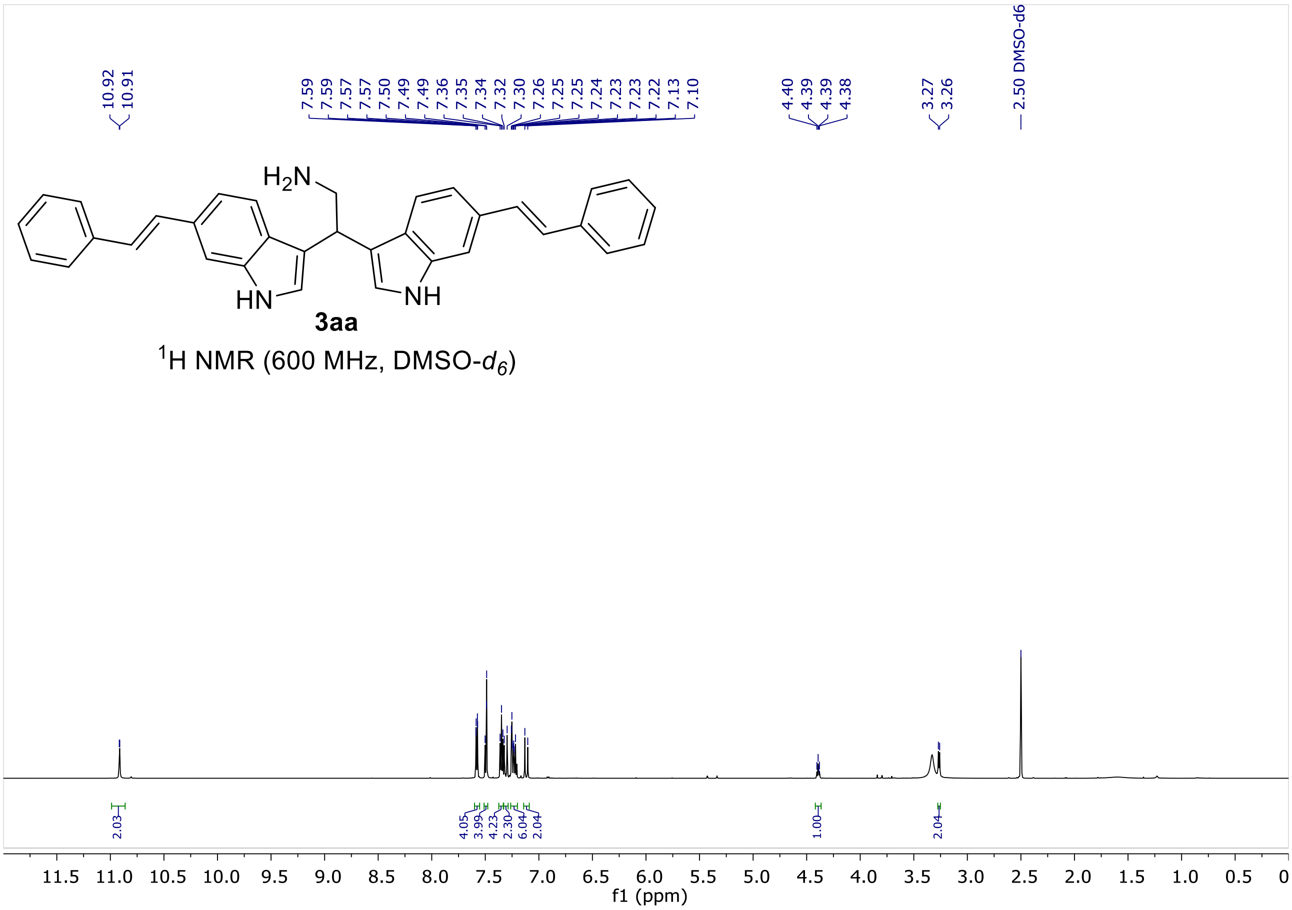


**3aa**

$^{1}H$ NMR (600 MHz, DMSO-$d_6$)

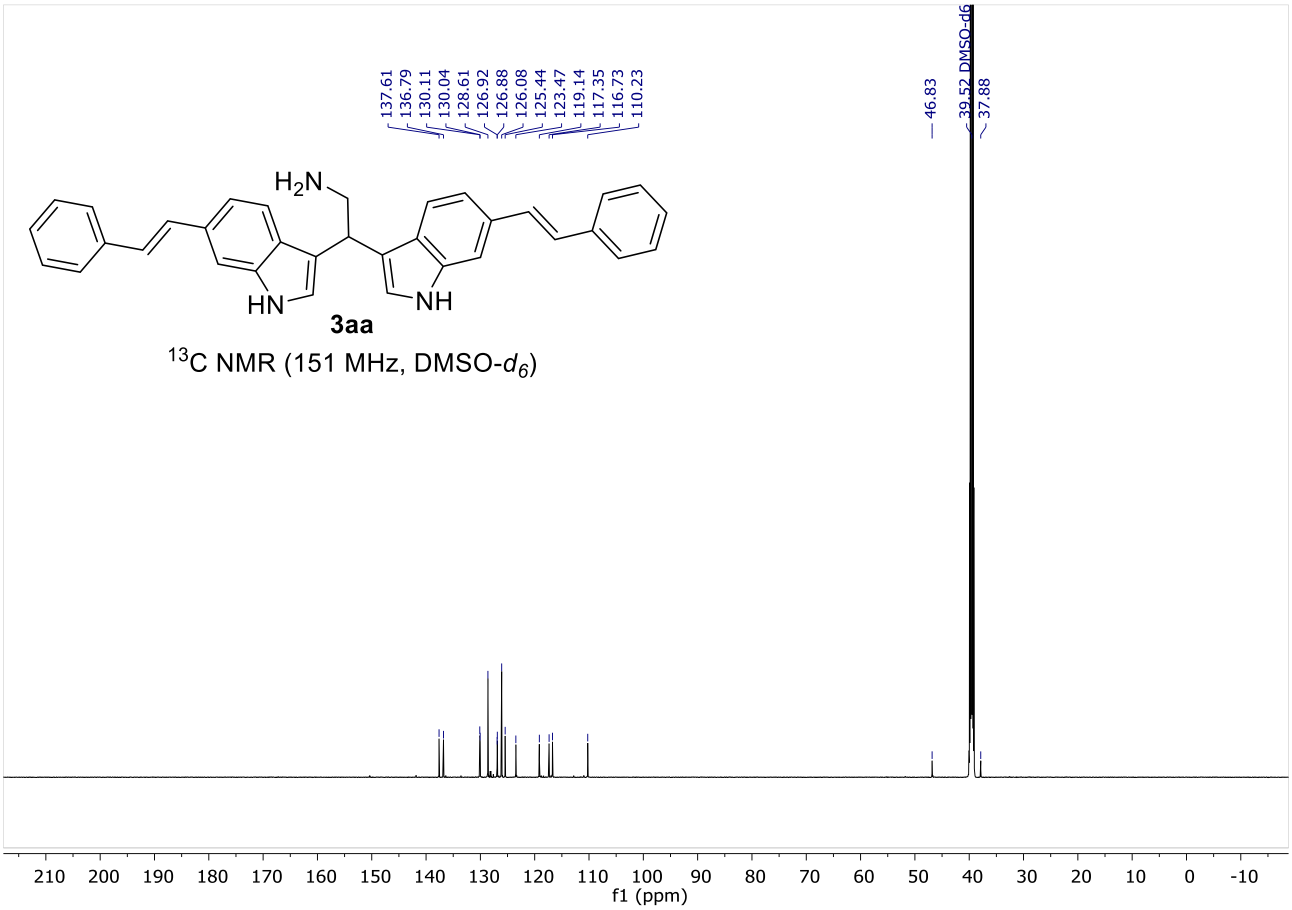


**3aa**

$^{13}C$ NMR (151 MHz, DMSO-$d_6$)

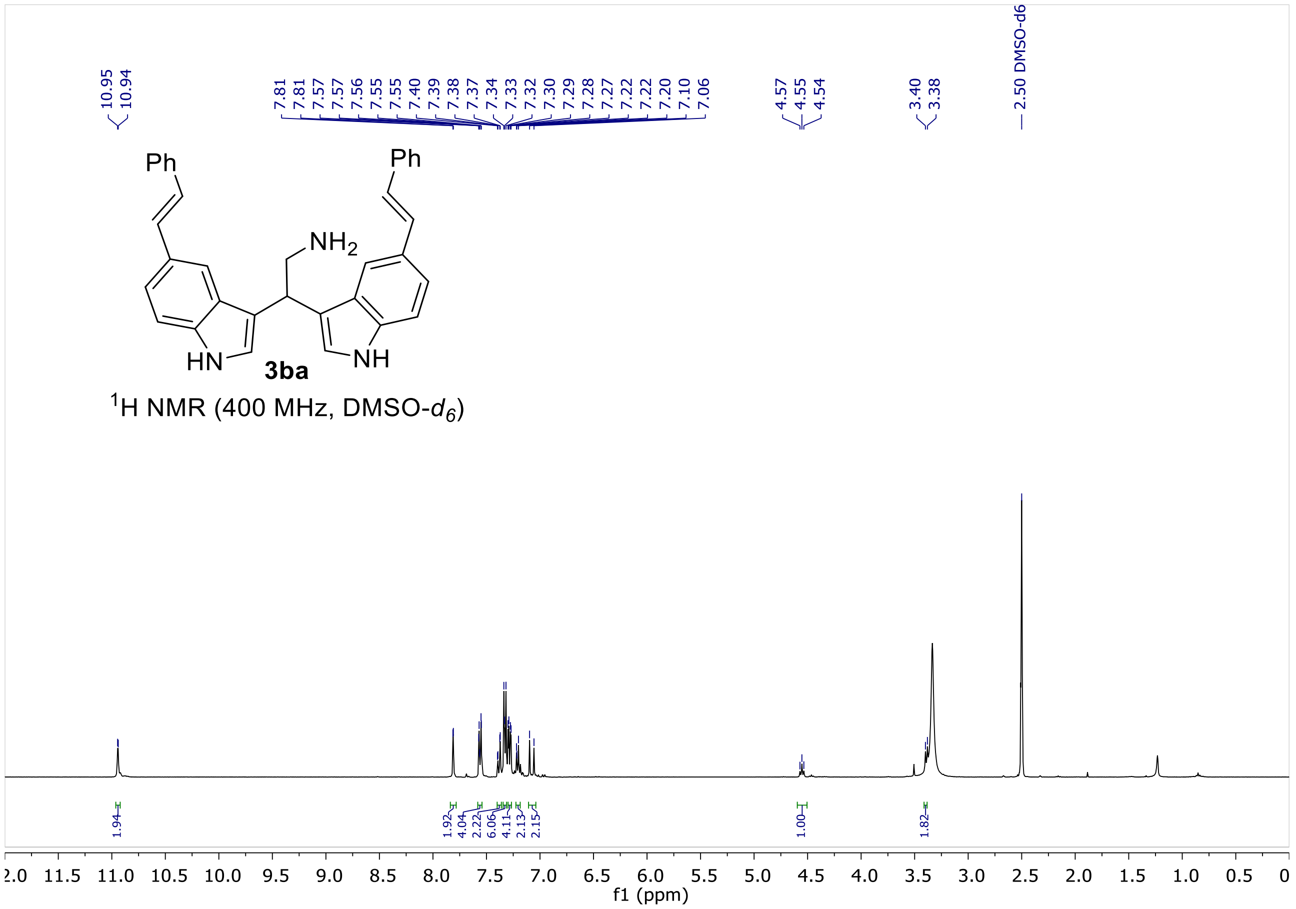
10.95
10.94
7.81
7.81
7.57
7.57
7.56
7.55
7.55
7.40
7.39
7.38
7.37
7.34
7.33
7.32
7.30
7.29
7.28
7.27
7.22
7.22
7.20
7.10
7.06
4.57
4.55
4.54
3.40
3.38
2.50 DMSO-d6
Ph
Ph
NH2
HN
NH
3ba
1H NMR (400 MHz, DMSO-d6)
1.94
1.92
4.04
2.22
6.06
4.11
2.13
2.15
1.00
1.82
f1 (ppm)

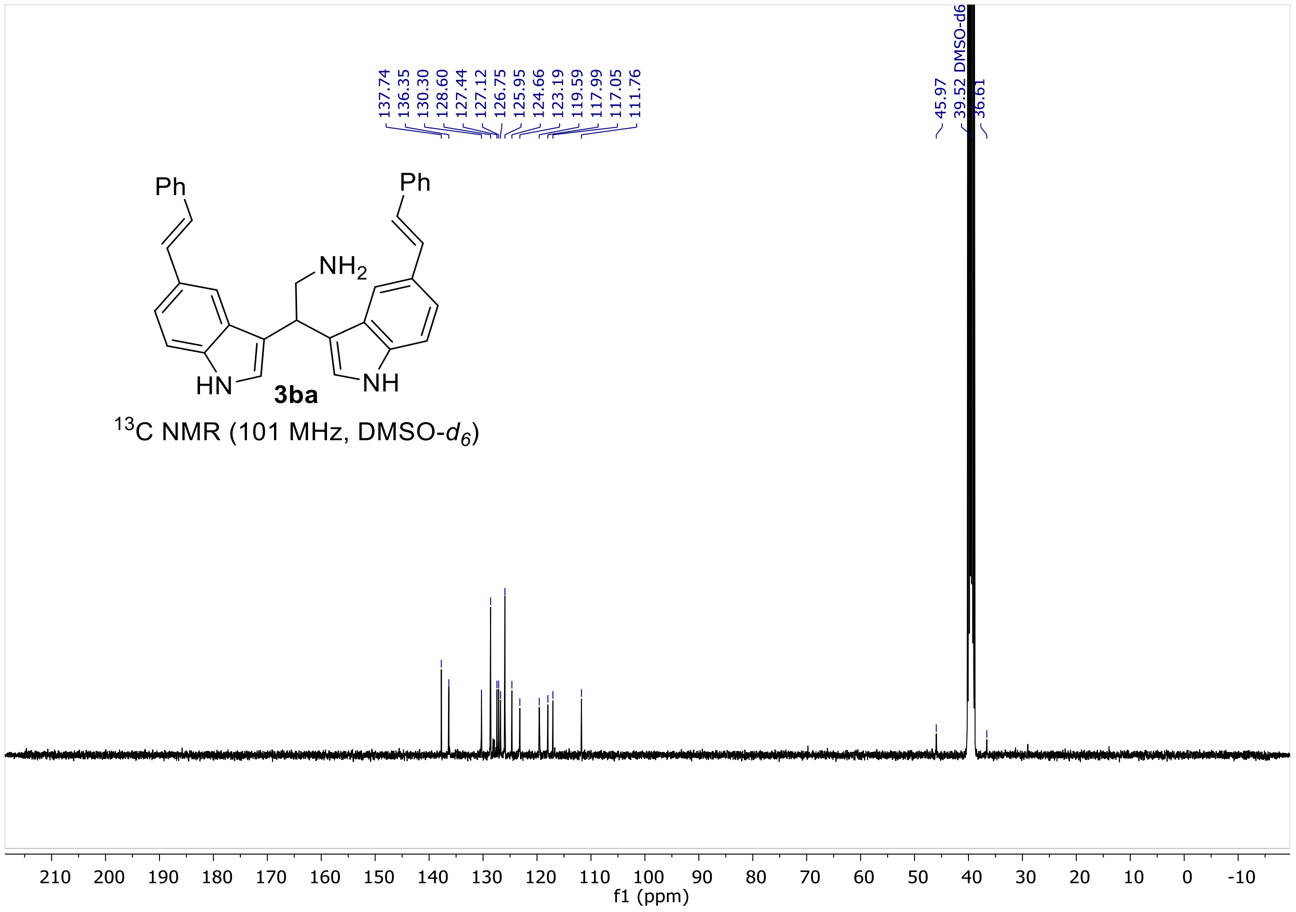
137.74
136.35
130.30
128.60
127.44
127.12
126.75
125.95
124.66
123.19
119.59
117.99
117.05
111.76
45.97
39.52 DMSO-d6
36.61
Ph
Ph
NH2
HN
NH
3ba
13C NMR (101 MHz, DMSO-d6)
f1 (ppm)

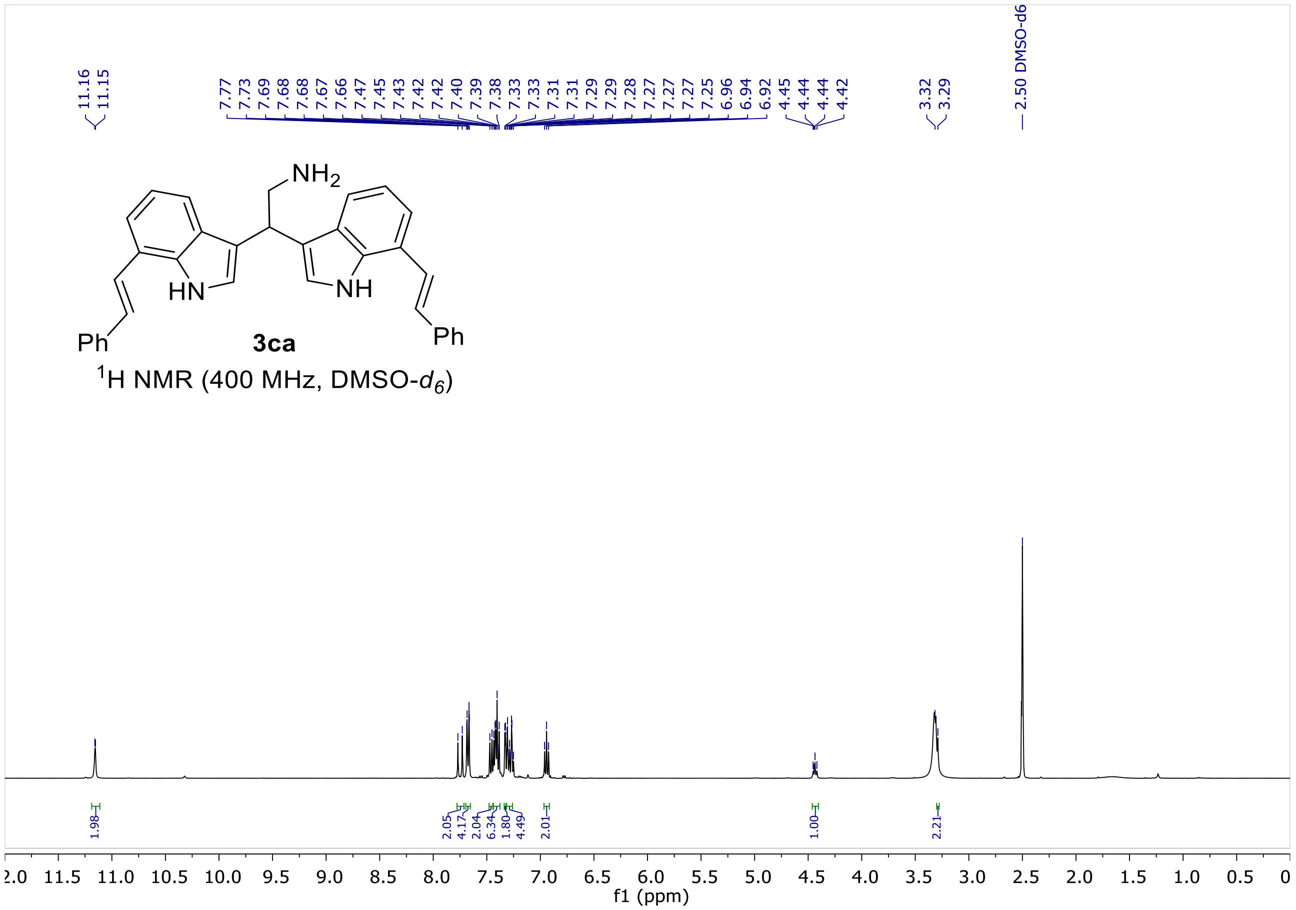
11.16
11.15
7.77
7.73
7.69
7.68
7.68
7.67
7.66
7.47
7.45
7.43
7.42
7.42
7.40
7.39
7.38
7.33
7.33
7.31
7.31
7.29
7.29
7.28
7.27
7.27
7.27
7.25
6.96
6.94
6.92
4.45
4.44
4.44
4.42
3.32
3.29
2.50 DMSO-d6
NH2
HN
NH
Ph
Ph
3ca
1H NMR (400 MHz, DMSO-d6)
1.98
2.05
4.17
2.04
6.34
1.80
4.49
2.01
1.00
2.21
f1 (ppm)
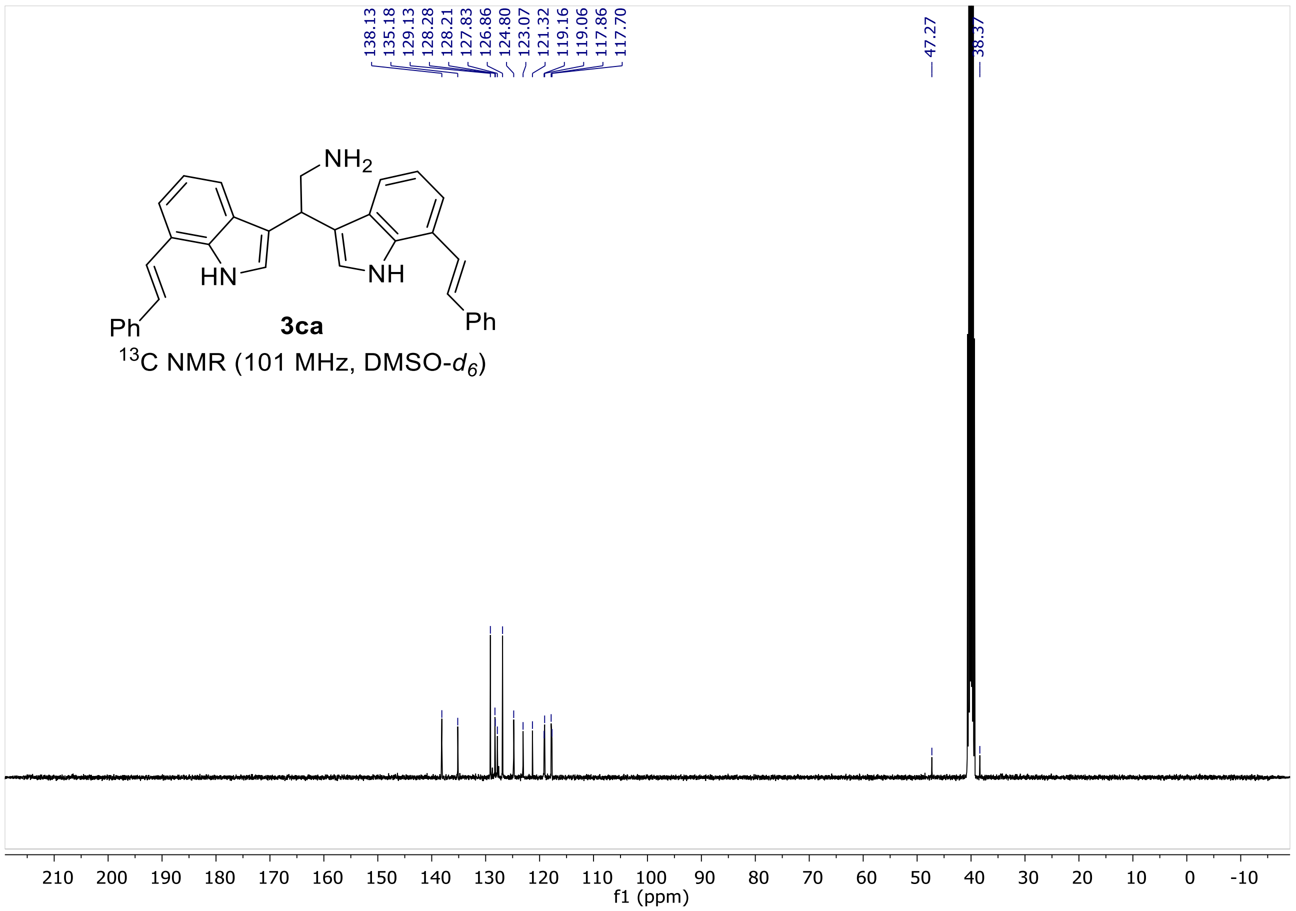
138.13
135.18
129.13
128.28
128.21
127.83
126.86
124.80
123.07
121.32
119.16
119.06
117.86
117.70
47.27
38.37
NH2
HN
NH
Ph
Ph
3ca
13C NMR (101 MHz, DMSO-d6)
f1 (ppm)

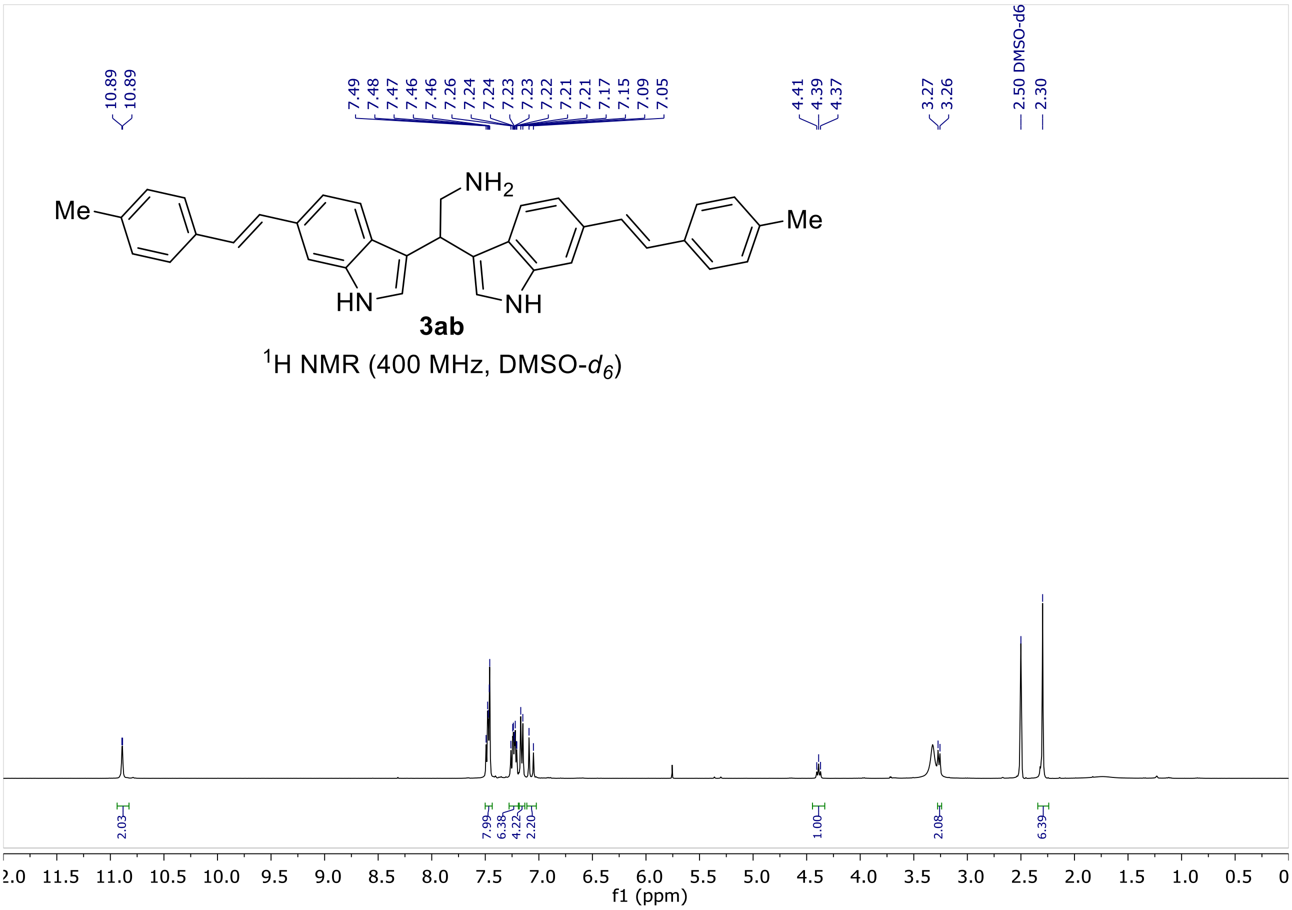


**3ab**

$^{1}$H NMR (400 MHz, DMSO-$d_6$)

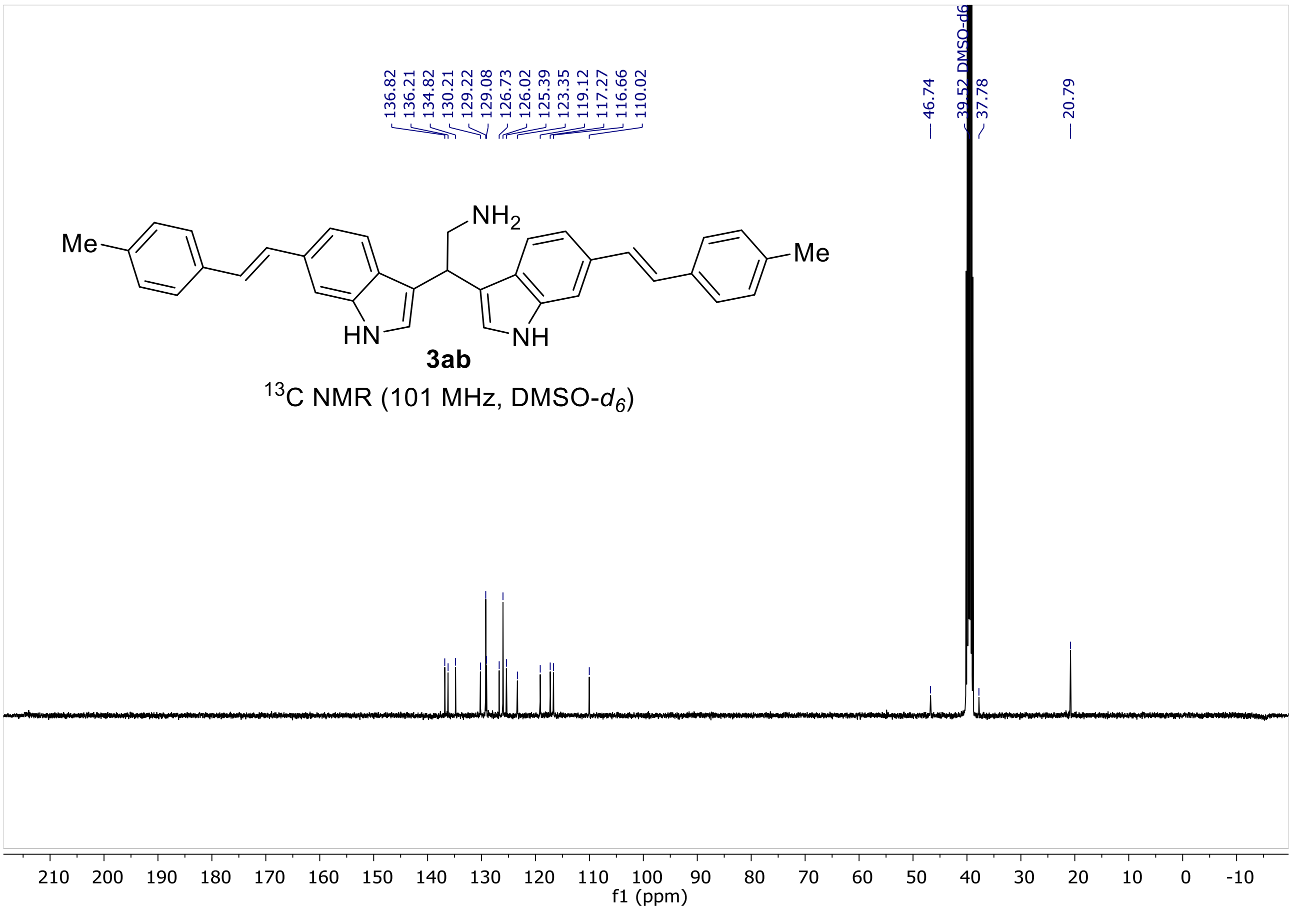


**3ab**

$^{13}$C NMR (101 MHz, DMSO-$d_6$)

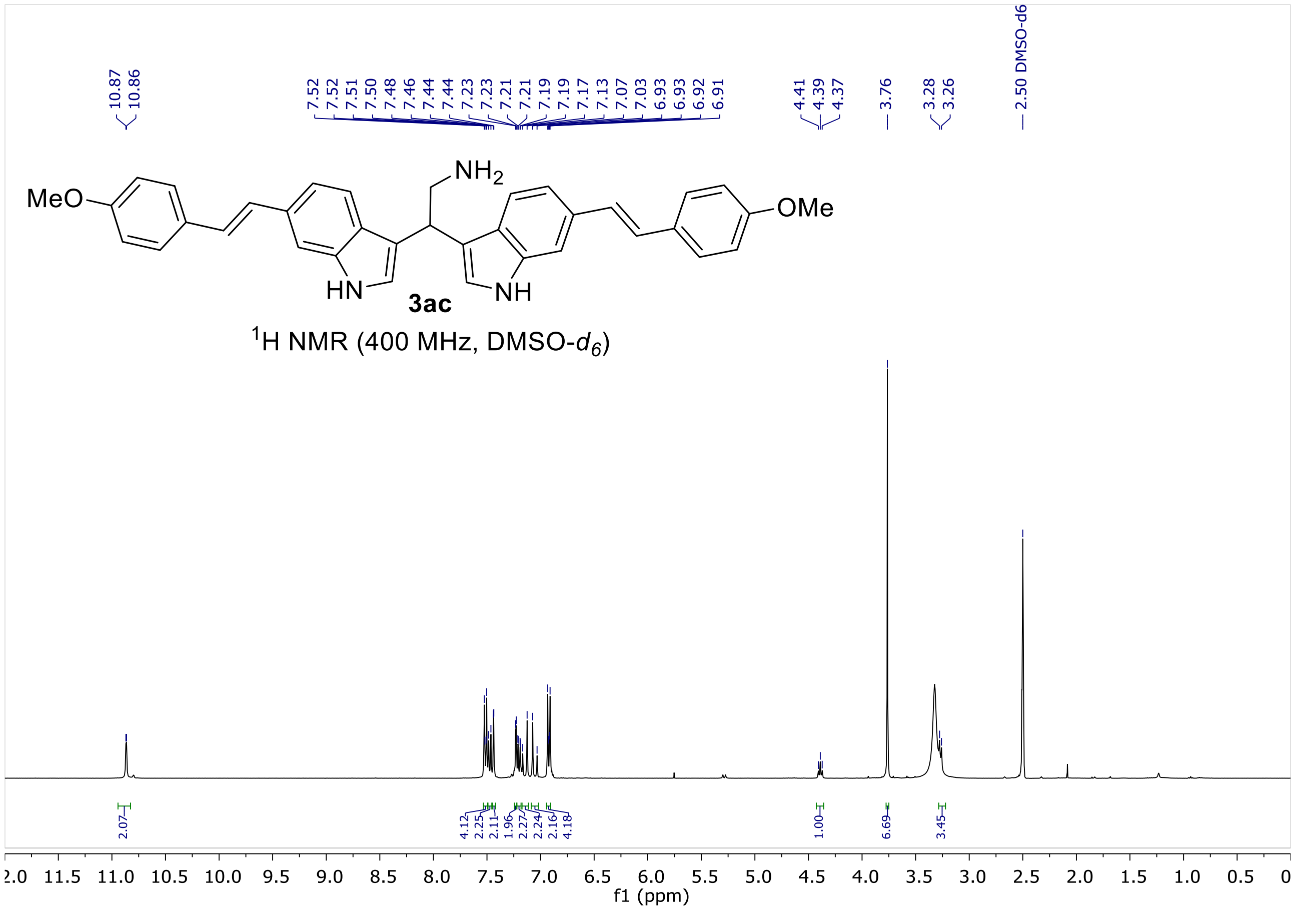
10.87
10.86
7.52
7.52
7.51
7.50
7.48
7.46
7.44
7.44
7.23
7.23
7.21
7.21
7.19
7.19
7.17
7.13
7.07
7.03
6.93
6.93
6.92
6.91
4.41
4.39
4.37
3.76
3.28
3.26
2.50 DMSO-d6
MeO
NH2
OMe
HN
3ac
NH
1H NMR (400 MHz, DMSO-d6)
2.07
4.12
2.25
2.11
1.96
2.27
2.24
2.16
4.18
1.00
6.69
3.45
f1 (ppm)

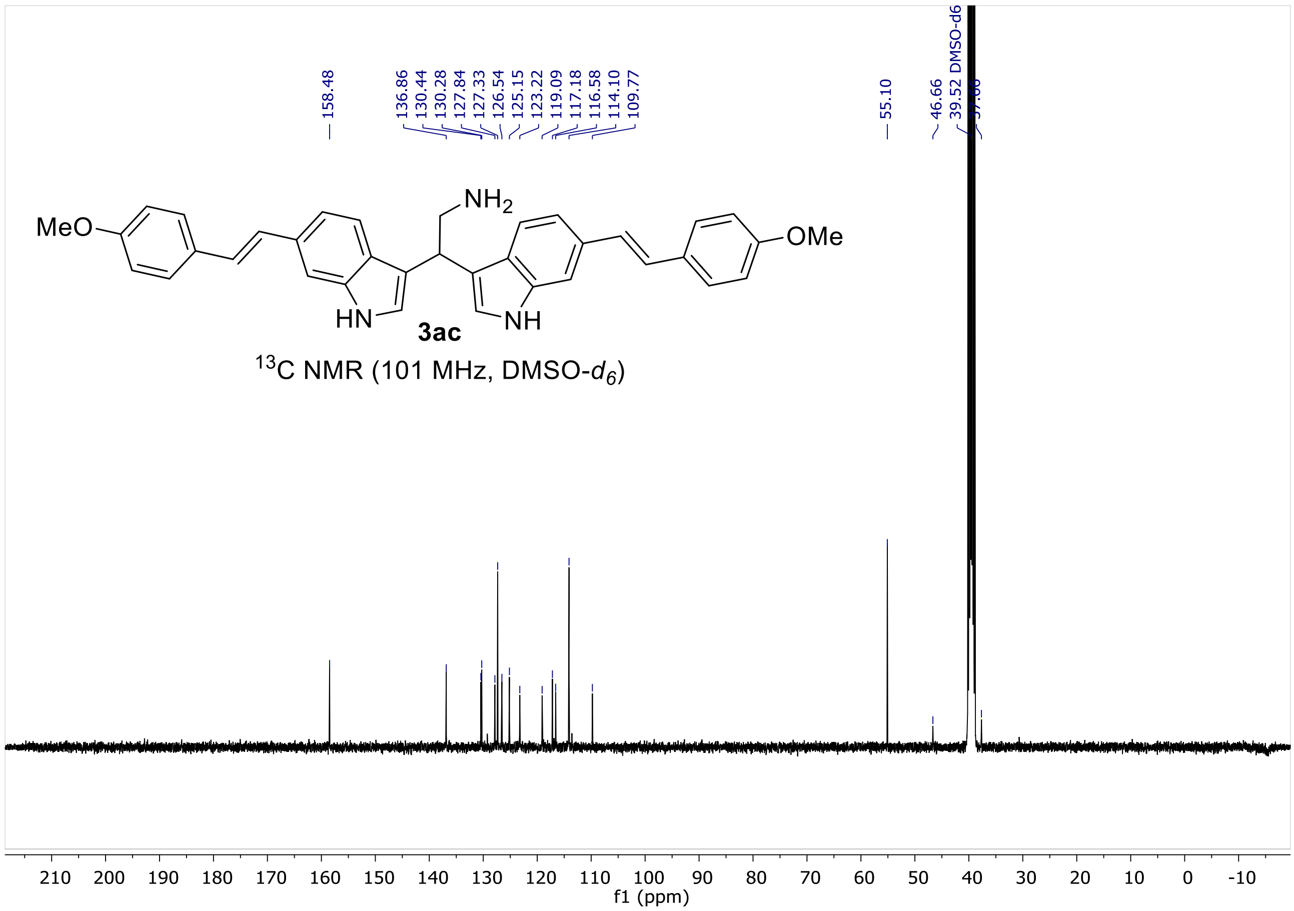
158.48
136.86
130.44
130.28
127.84
127.33
126.54
125.15
123.22
119.09
117.18
116.58
114.10
109.77
55.10
46.66
39.52 DMSO-d6
MeO
NH2
OMe
HN
3ac
NH
13C NMR (101 MHz, DMSO-d6)
f1 (ppm)

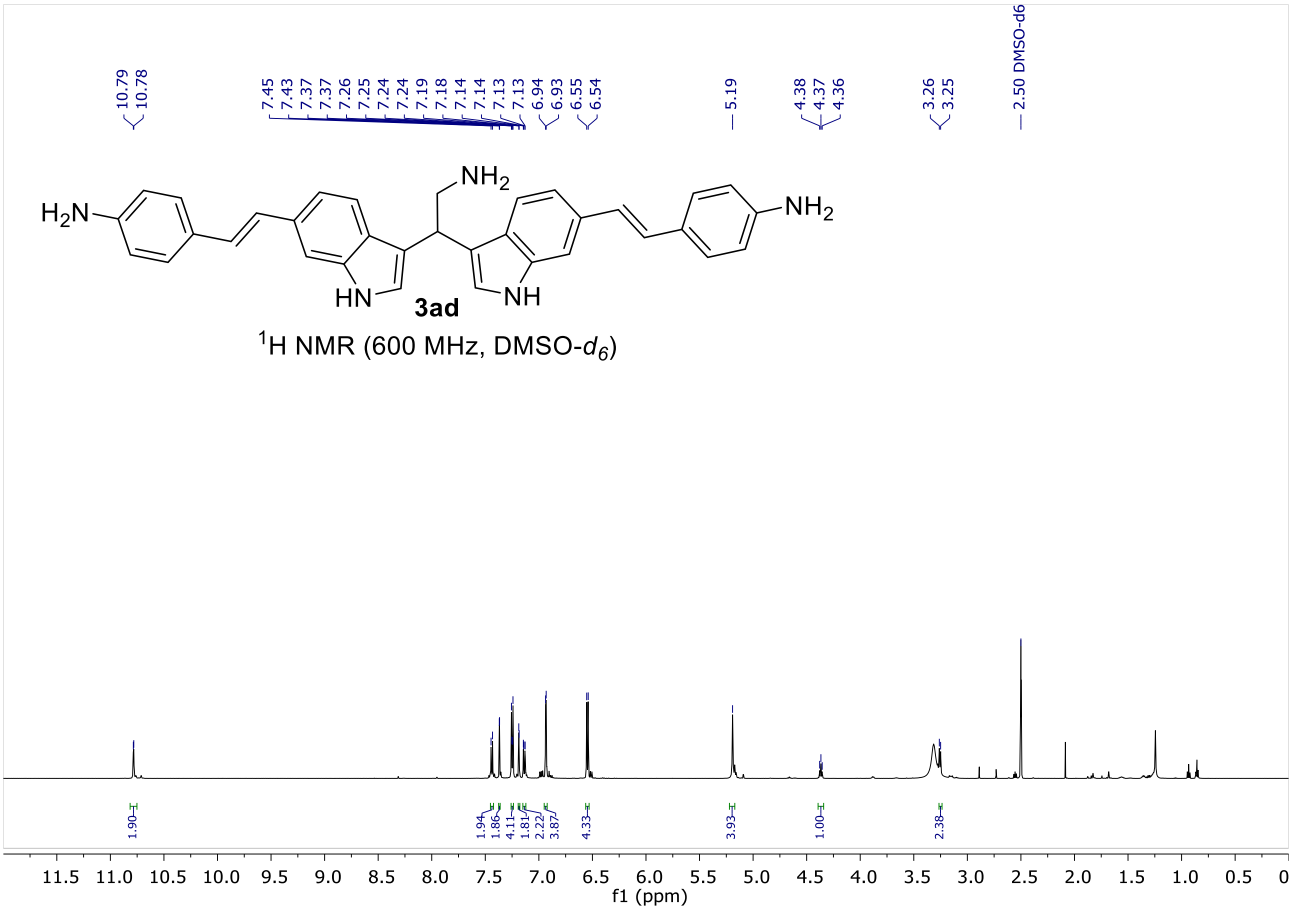


$^{1}H$ NMR (600 MHz, DMSO-$d_6$)

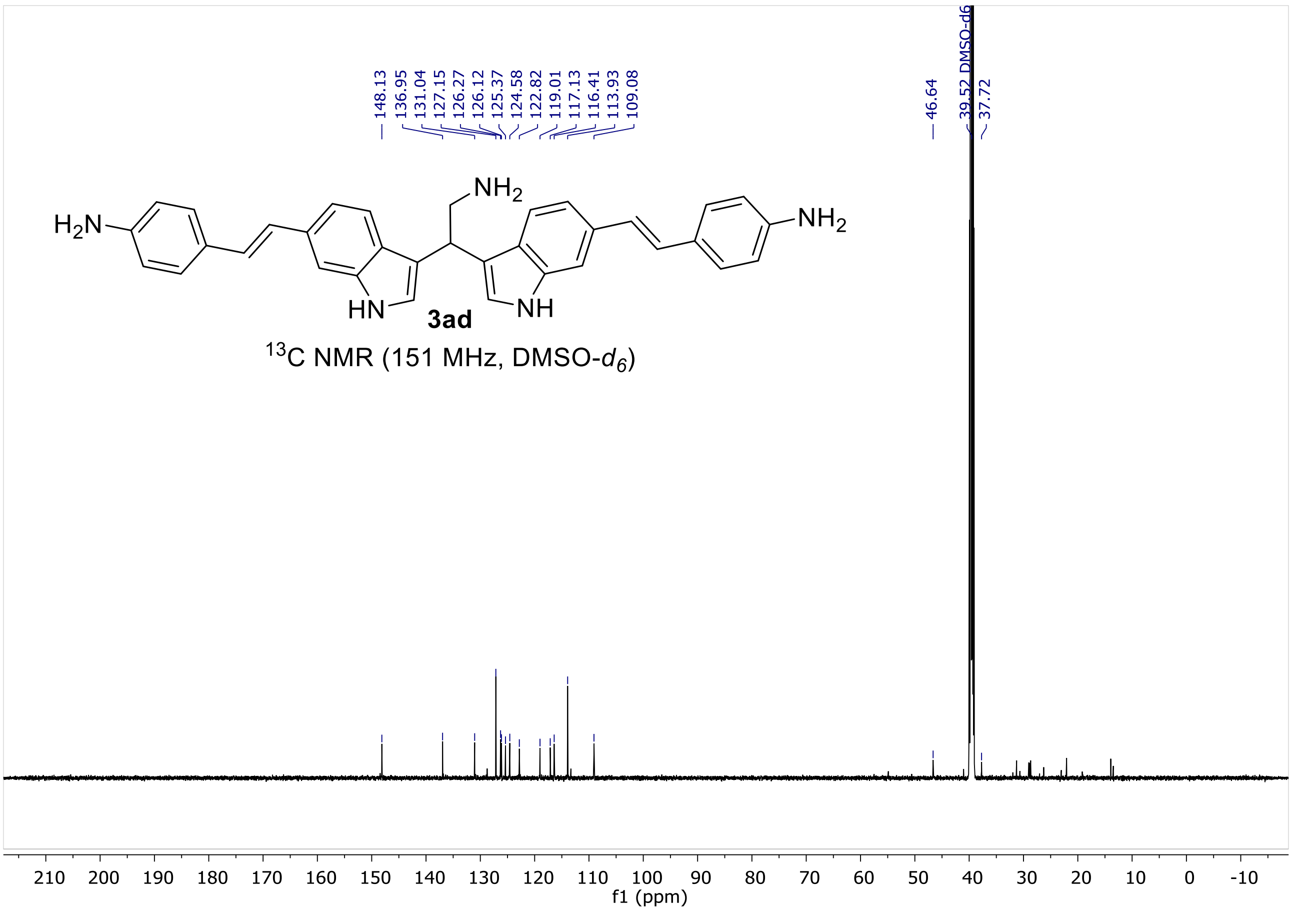


$^{13}C$ NMR (151 MHz, DMSO-$d_6$)

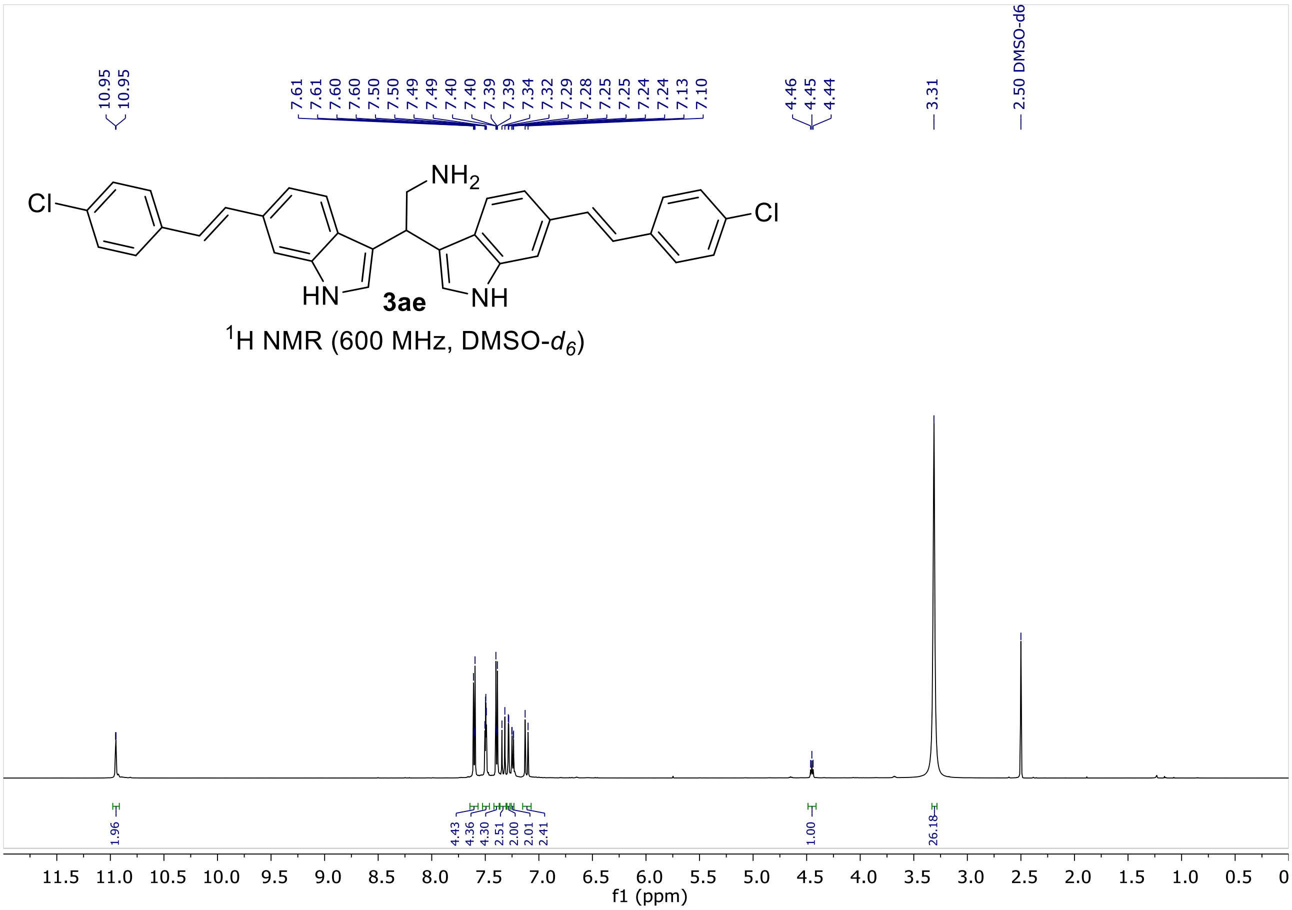
10.95
10.95
7.61
7.61
7.60
7.50
7.50
7.49
7.49
7.40
7.40
7.39
7.39
7.34
7.32
7.29
7.28
7.25
7.25
7.24
7.24
7.13
7.10
4.46
4.45
4.44
3.31
2.50 DMSO-d6
NH2
Cl
Cl
HN
NH
3ae
1H NMR (600 MHz, DMSO-d6)
1.96
4.43
4.36
4.30
2.51
2.00
2.01
2.41
1.00
26.18
11.5 11.0 10.5 10.0 9.5 9.0 8.5 8.0 7.5 7.0 6.5 6.0 5.5 5.0 4.5 4.0 3.5 3.0 2.5 2.0 1.5 1.0 0.5 0
f1 (ppm)

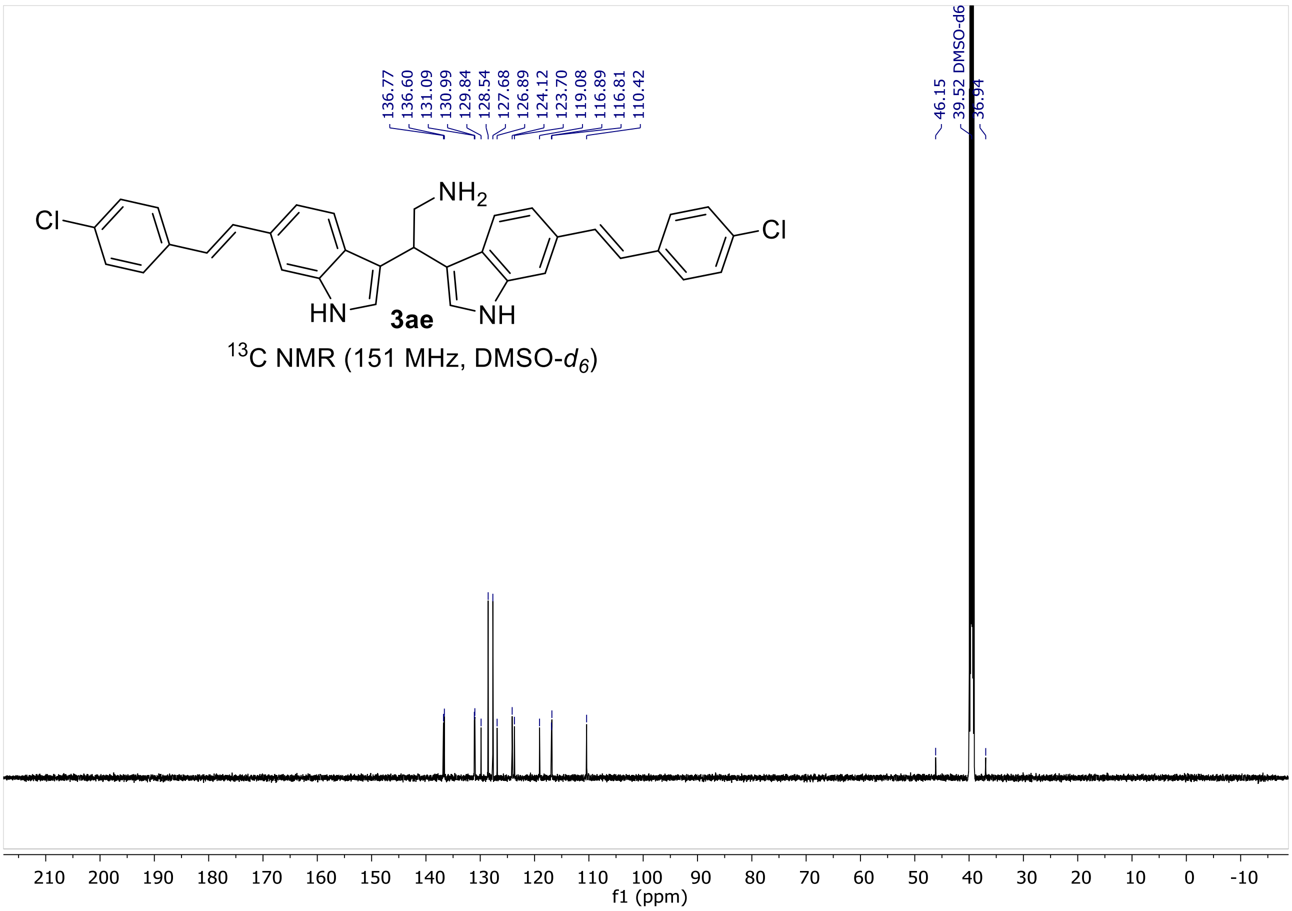
136.77
136.60
131.09
130.99
129.84
128.54
127.68
126.89
124.12
123.70
119.08
116.89
116.81
110.42
46.15
39.52 DMSO-d6
36.94
NH2
Cl
Cl
HN
NH
3ae
13C NMR (151 MHz, DMSO-d6)
210 200 190 180 170 160 150 140 130 120 110 100 90 80 70 60 50 40 30 20 10 0 -10
f1 (ppm)

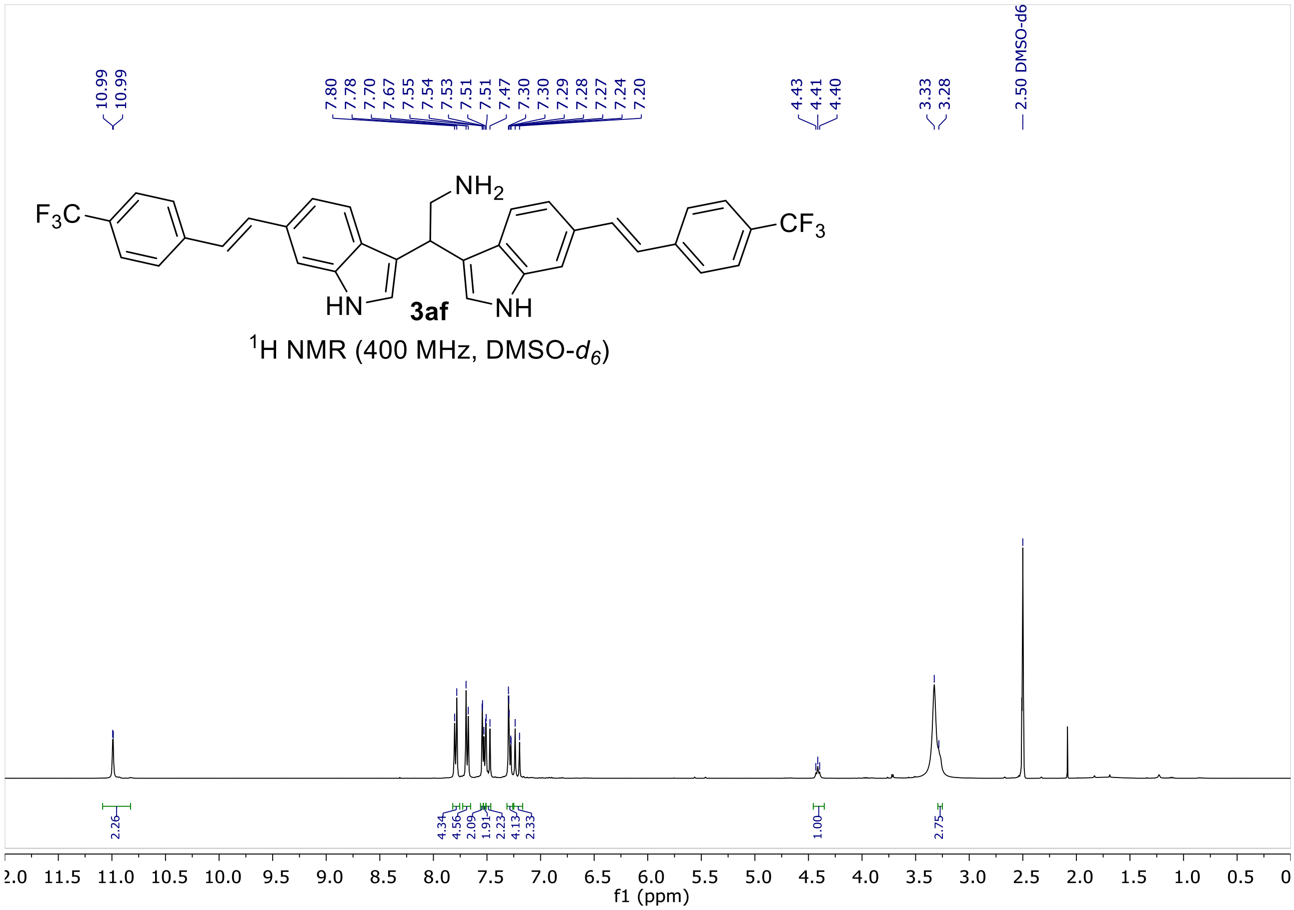
10.99
10.99
7.80
7.78
7.70
7.67
7.55
7.54
7.53
7.51
7.51
7.47
7.30
7.30
7.29
7.28
7.27
7.24
7.20
4.43
4.41
4.40
3.33
3.28
2.50 DMSO-d6
$NH_2$
$F_3C$
$CF_3$
HN
NH
3af
$^1H$ NMR (400 MHz, DMSO-$d_6$)
2.26
4.34
4.56
2.09
1.91
2.23
4.13
2.33
1.00
2.75
f1 (ppm)

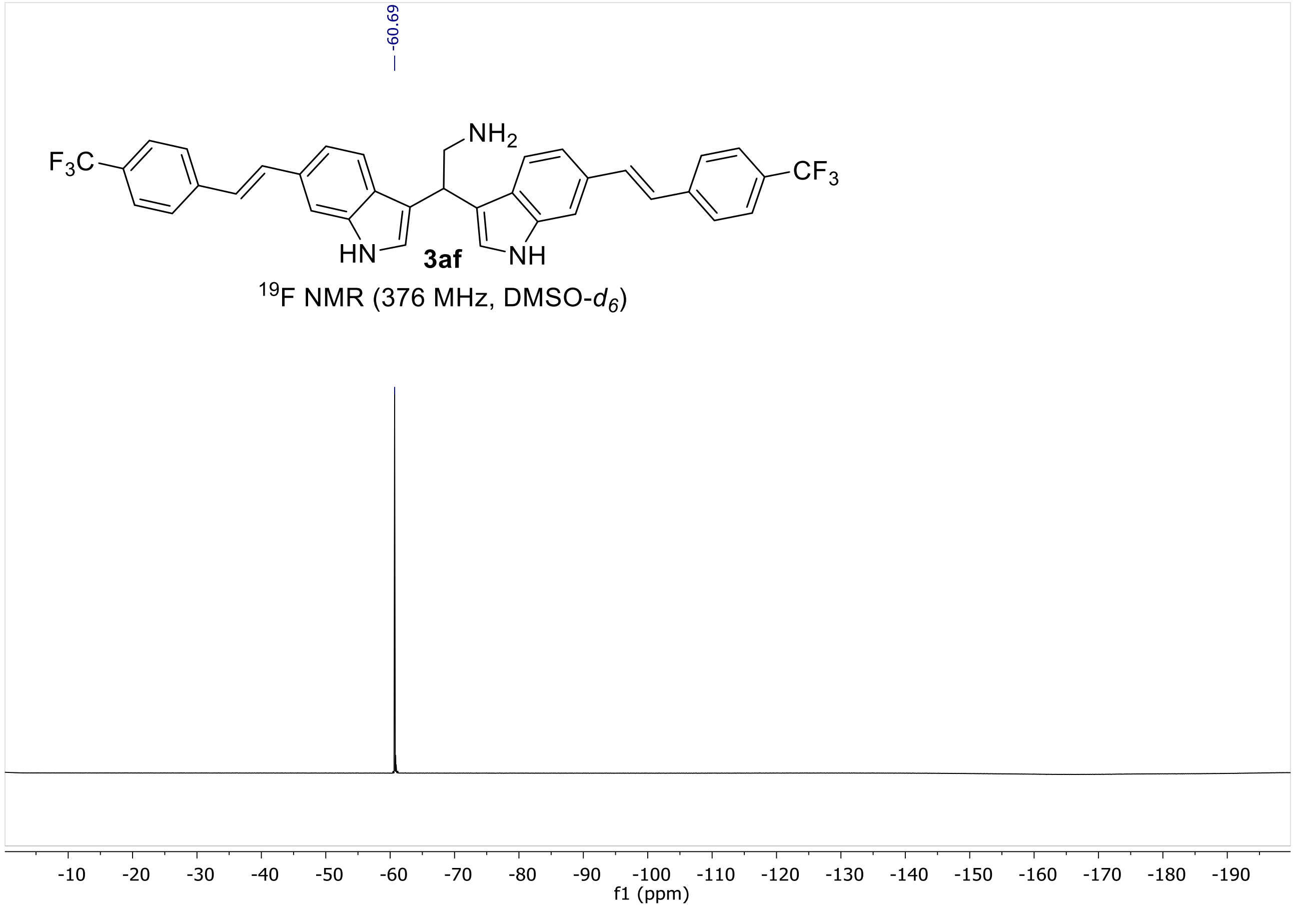
-60.69
$NH_2$
$F_3C$
$CF_3$
HN
NH
3af
$^{19}F$ NMR (376 MHz, DMSO-$d_6$)
f1 (ppm)

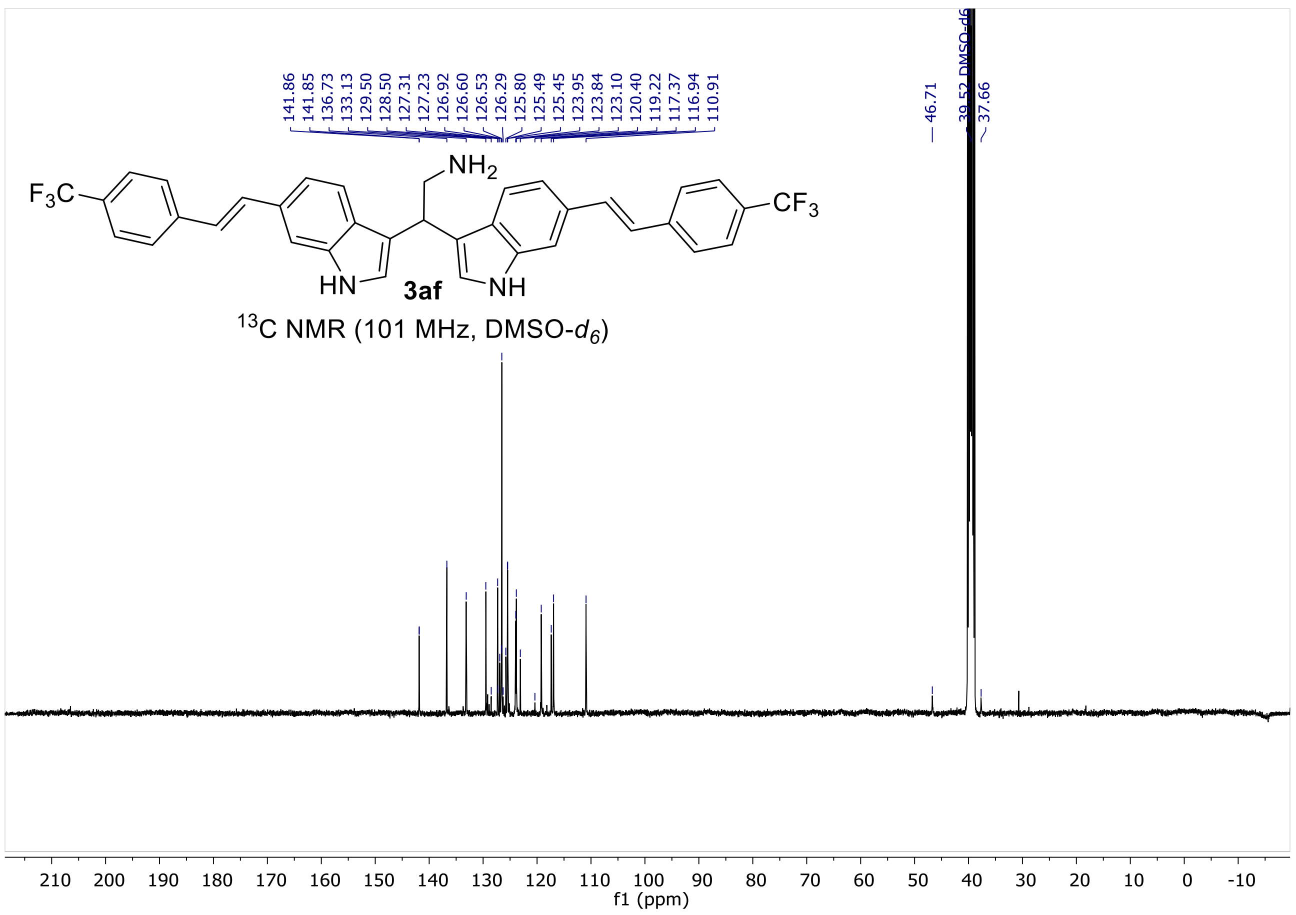

141.86
141.85
136.73
133.13
129.50
128.50
127.31
127.23
126.92
126.60
126.53
126.29
125.80
125.49
125.45
123.95
123.84
123.10
120.40
119.22
117.37
116.94
110.91
46.71
39.52 DMSO-d6
37.66
3af
13C NMR (101 MHz, DMSO-d6)
f1 (ppm)

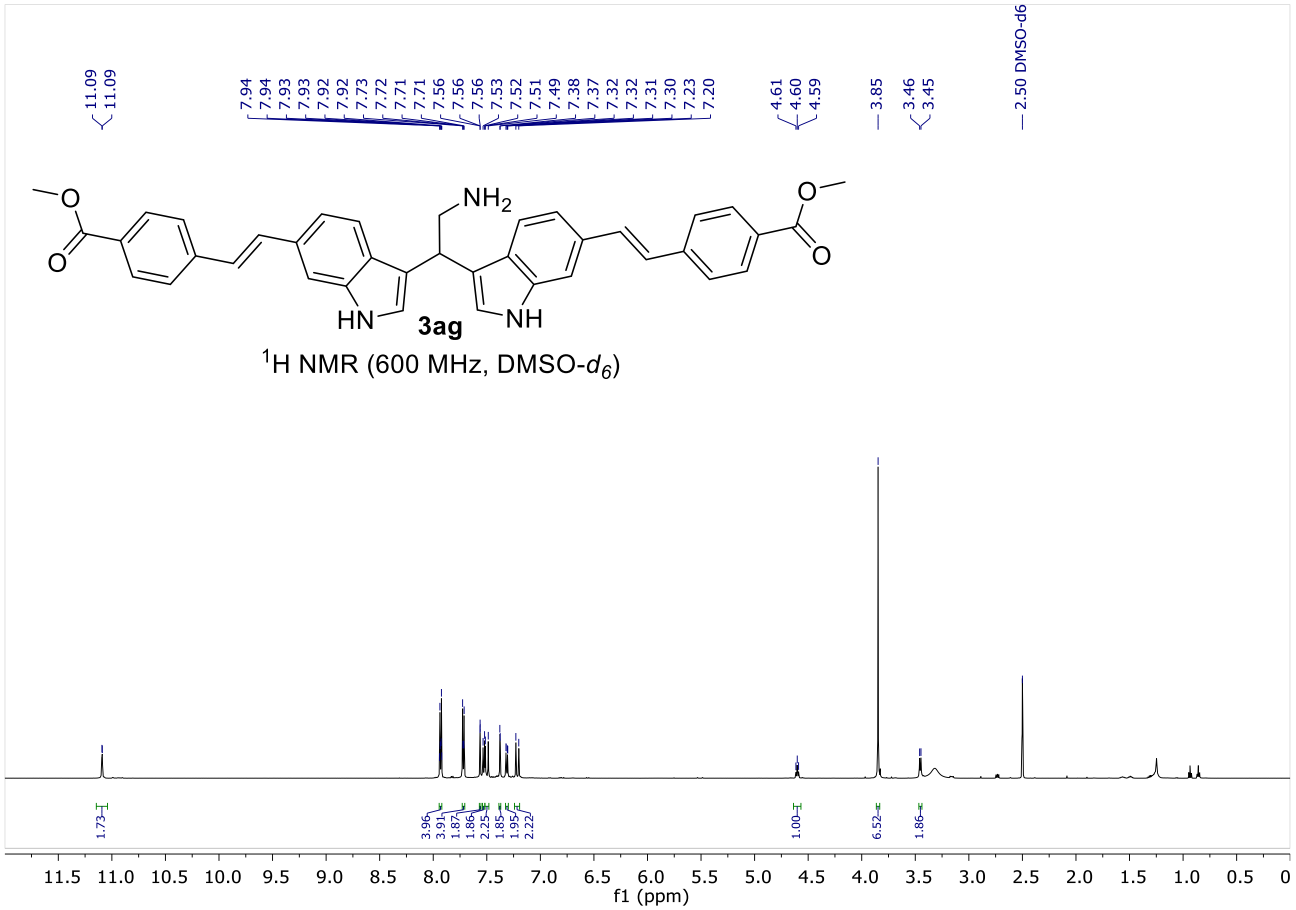
11.09
11.09
7.94
7.94
7.93
7.93
7.92
7.92
7.73
7.72
7.71
7.71
7.56
7.56
7.56
7.53
7.52
7.51
7.49
7.38
7.37
7.32
7.31
7.30
7.23
7.20
4.61
4.60
4.59
3.85
3.46
3.45
2.50 DMSO-d6
NH2
O
O
HN
3ag
NH
1H NMR (600 MHz, DMSO-d6)
1.73
3.96
3.91
1.87
1.86
2.25
1.85
1.95
2.22
1.00
6.52
1.86
11.5 11.0 10.5 10.0 9.5 9.0 8.5 8.0 7.5 7.0 6.5 6.0 5.5 5.0 4.5 4.0 3.5 3.0 2.5 2.0 1.5 1.0 0.5 0
f1 (ppm)

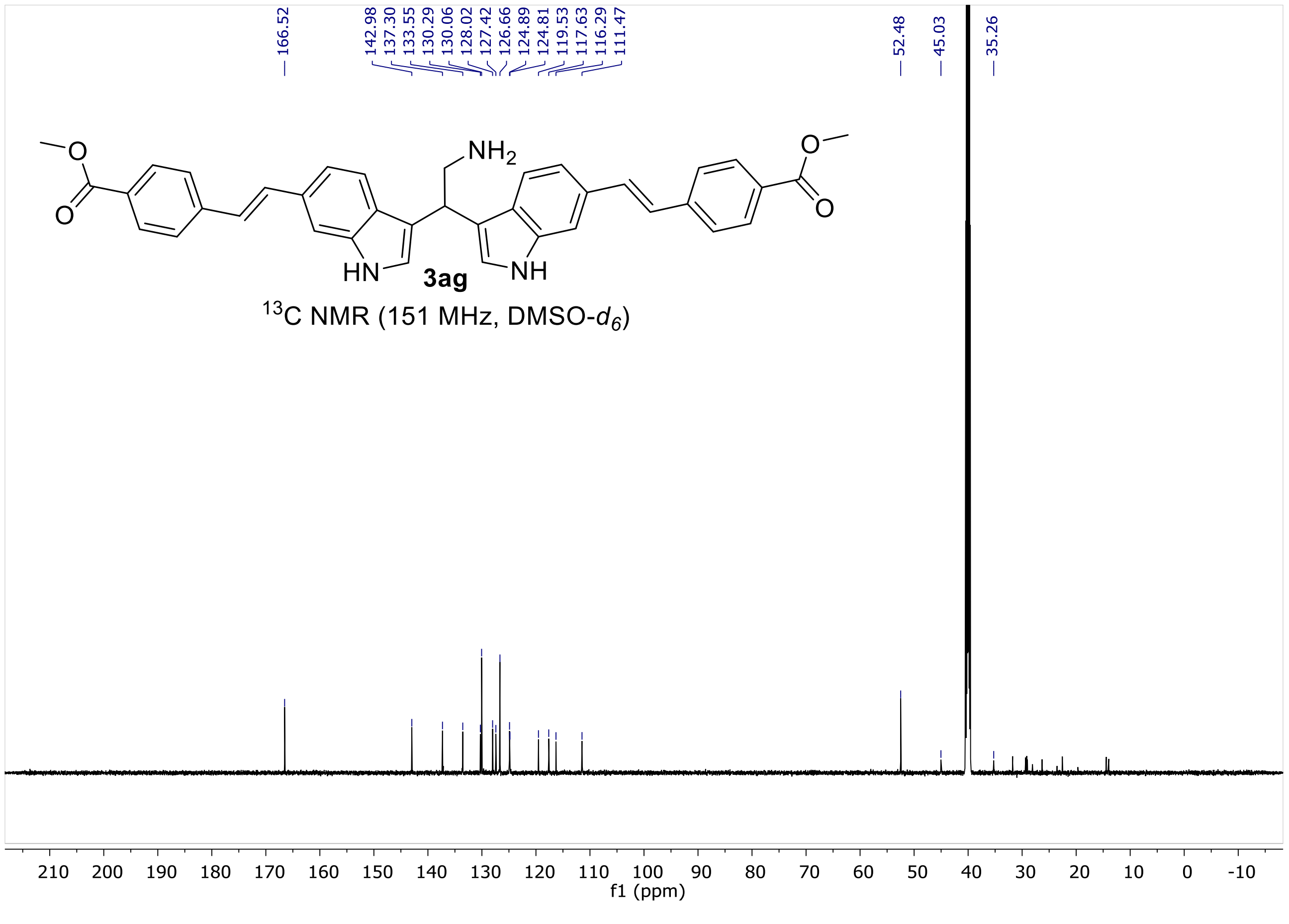
166.52
142.98
137.30
133.55
130.29
130.06
128.02
127.42
126.66
124.89
124.81
119.53
117.63
116.29
111.47
52.48
45.03
35.26
NH2
O
O
HN
3ag
NH
13C NMR (151 MHz, DMSO-d6)
210 200 190 180 170 160 150 140 130 120 110 100 90 80 70 60 50 40 30 20 10 0 -10
f1 (ppm)

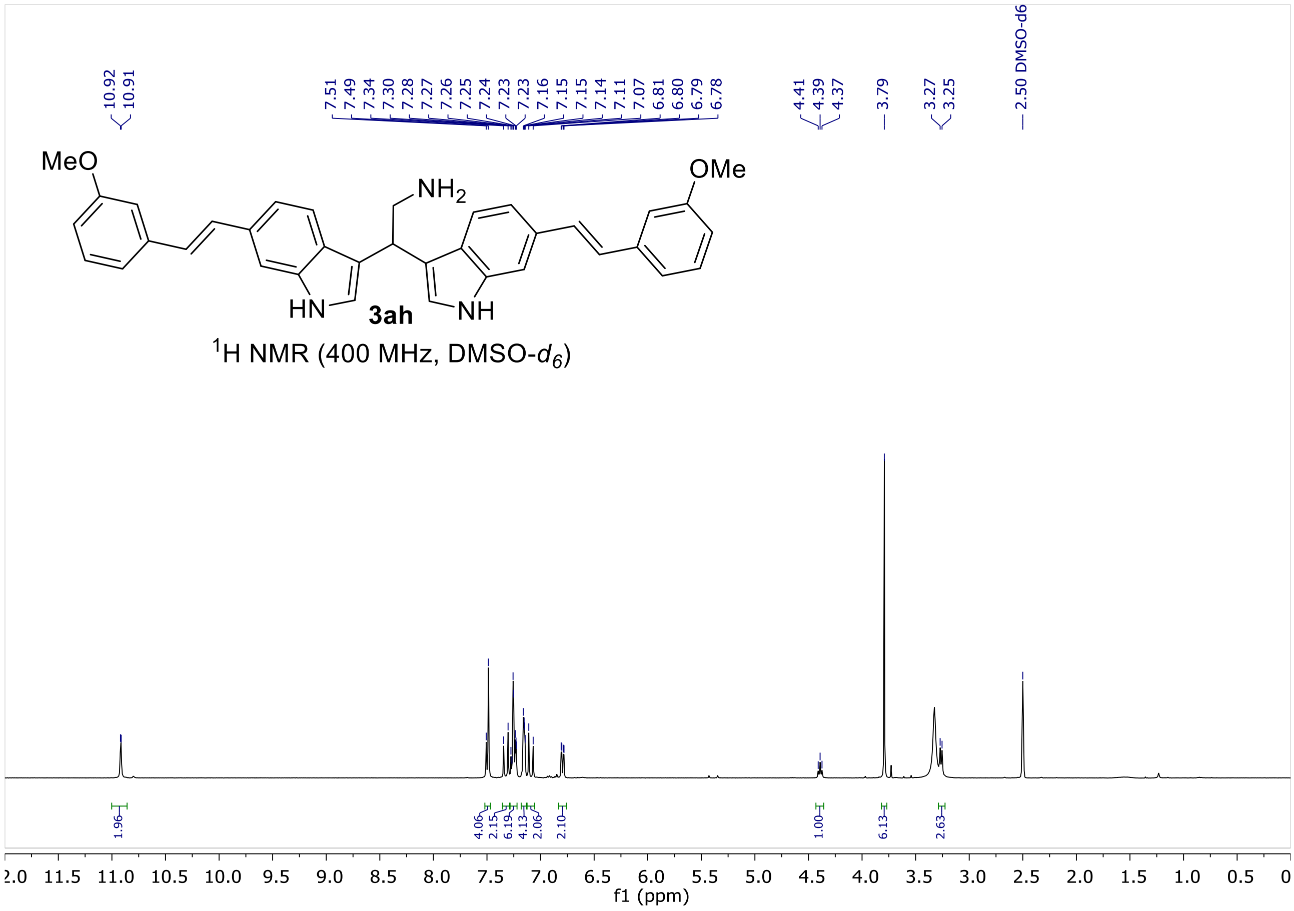
10.92
10.91
7.51
7.49
7.34
7.30
7.28
7.27
7.26
7.25
7.24
7.23
7.23
7.16
7.15
7.15
7.14
7.11
7.07
6.81
6.80
6.79
6.78
4.41
4.39
4.37
3.79
3.27
3.25
2.50 DMSO-d6
MeO
OMe
NH2
HN
3ah
NH
1H NMR (400 MHz, DMSO-d6)
1.96
4.06
2.15
6.19
4.13
2.06
2.10
1.00
6.13
2.63
f1 (ppm)

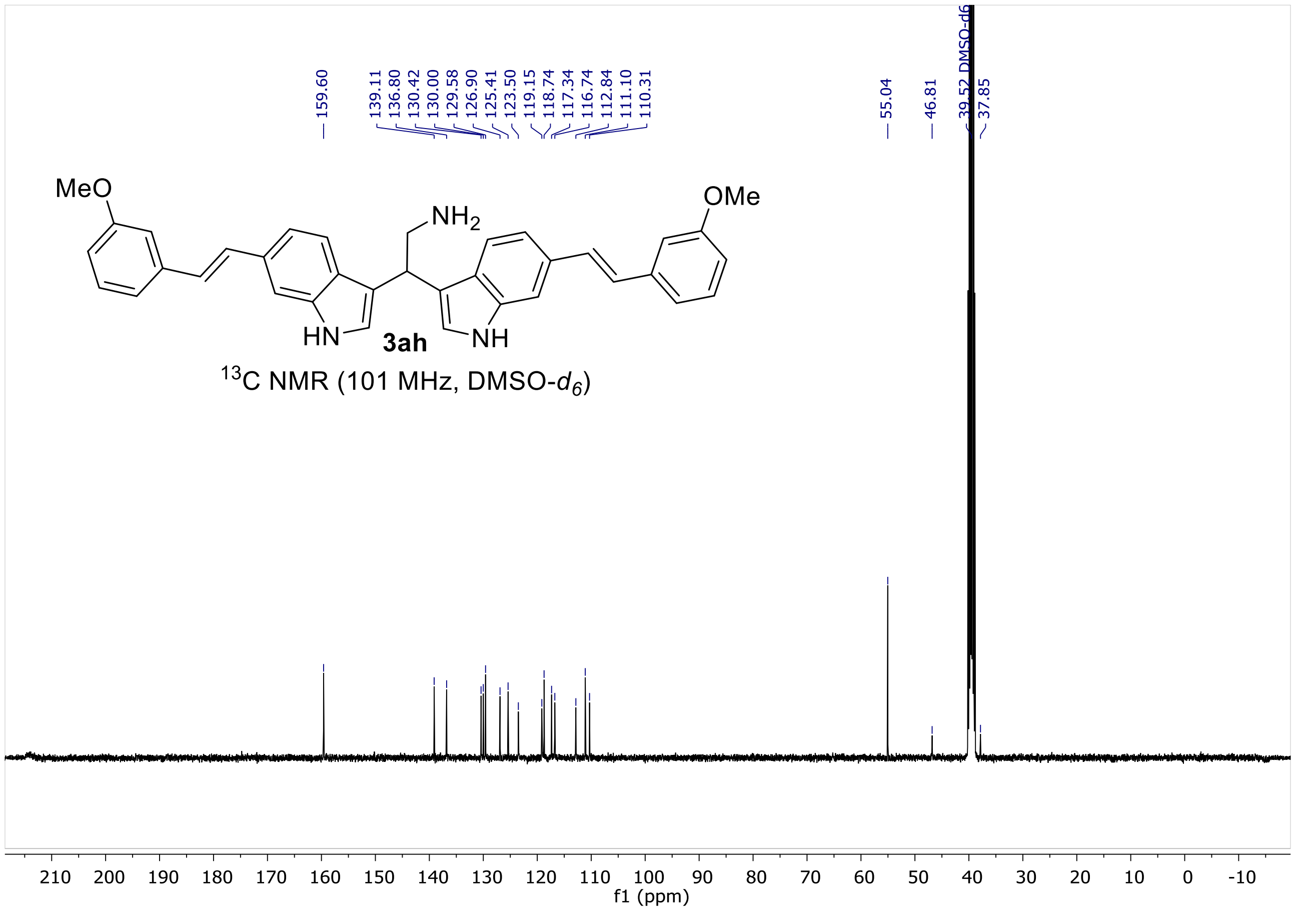
159.60
139.11
136.80
130.42
130.00
129.58
126.90
125.41
123.50
119.15
118.74
117.34
116.74
112.84
111.10
110.31
55.04
46.81
39.52 DMSO-d6
37.85
MeO
OMe
NH2
HN
3ah
NH
13C NMR (101 MHz, DMSO-d6)
f1 (ppm)

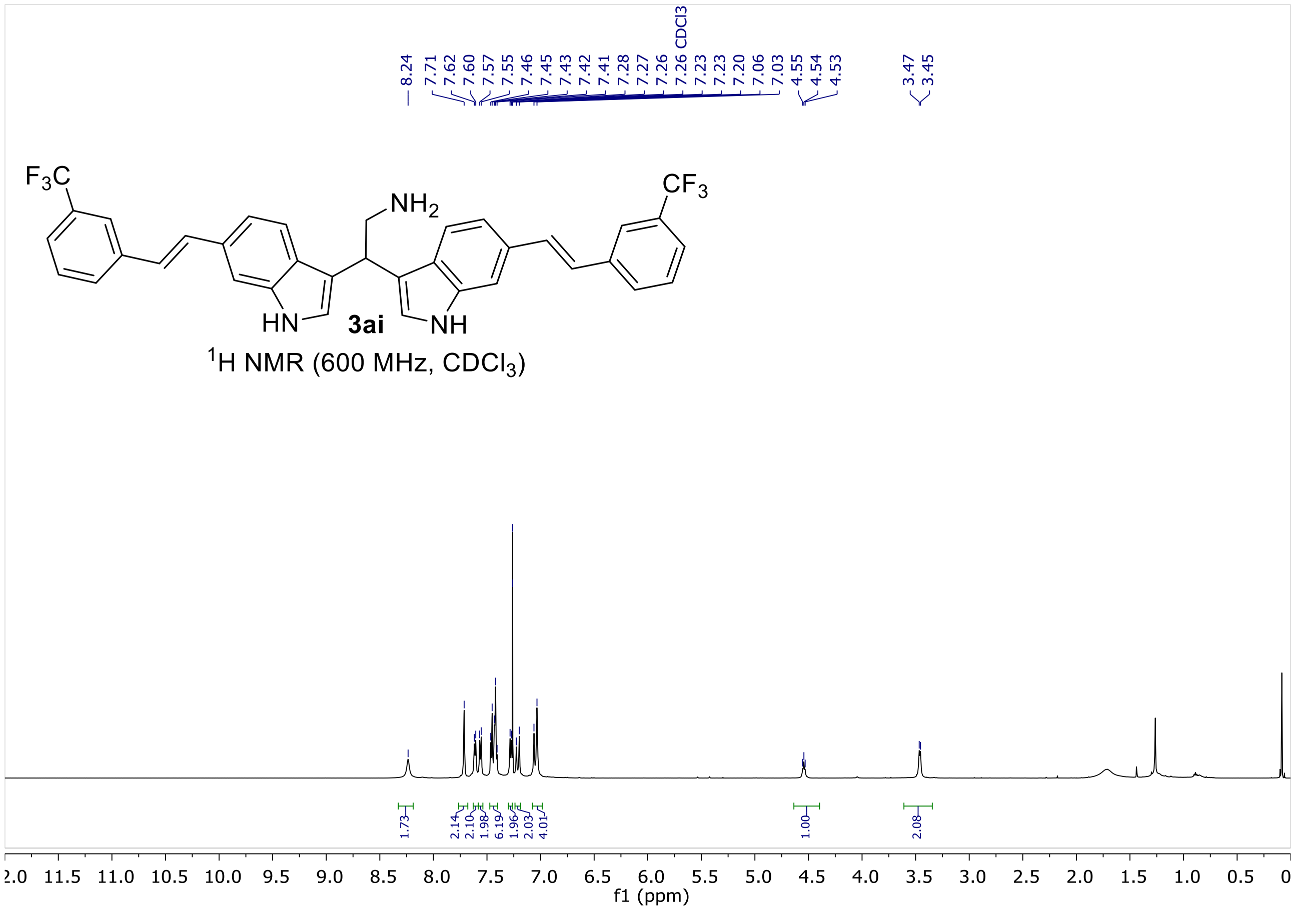
8.24
7.71
7.62
7.60
7.57
7.55
7.46
7.45
7.43
7.42
7.41
7.28
7.27
7.26
7.26 CDCl3
7.23
7.23
7.20
7.06
7.03
4.55
4.54
4.53
3.47
3.45
F3C
NH2
CF3
HN
3ai
NH
1H NMR (600 MHz, CDCl3)
1.73
2.14
2.10
1.98
6.19
1.96
2.03
4.01
1.00
2.08
f1 (ppm)

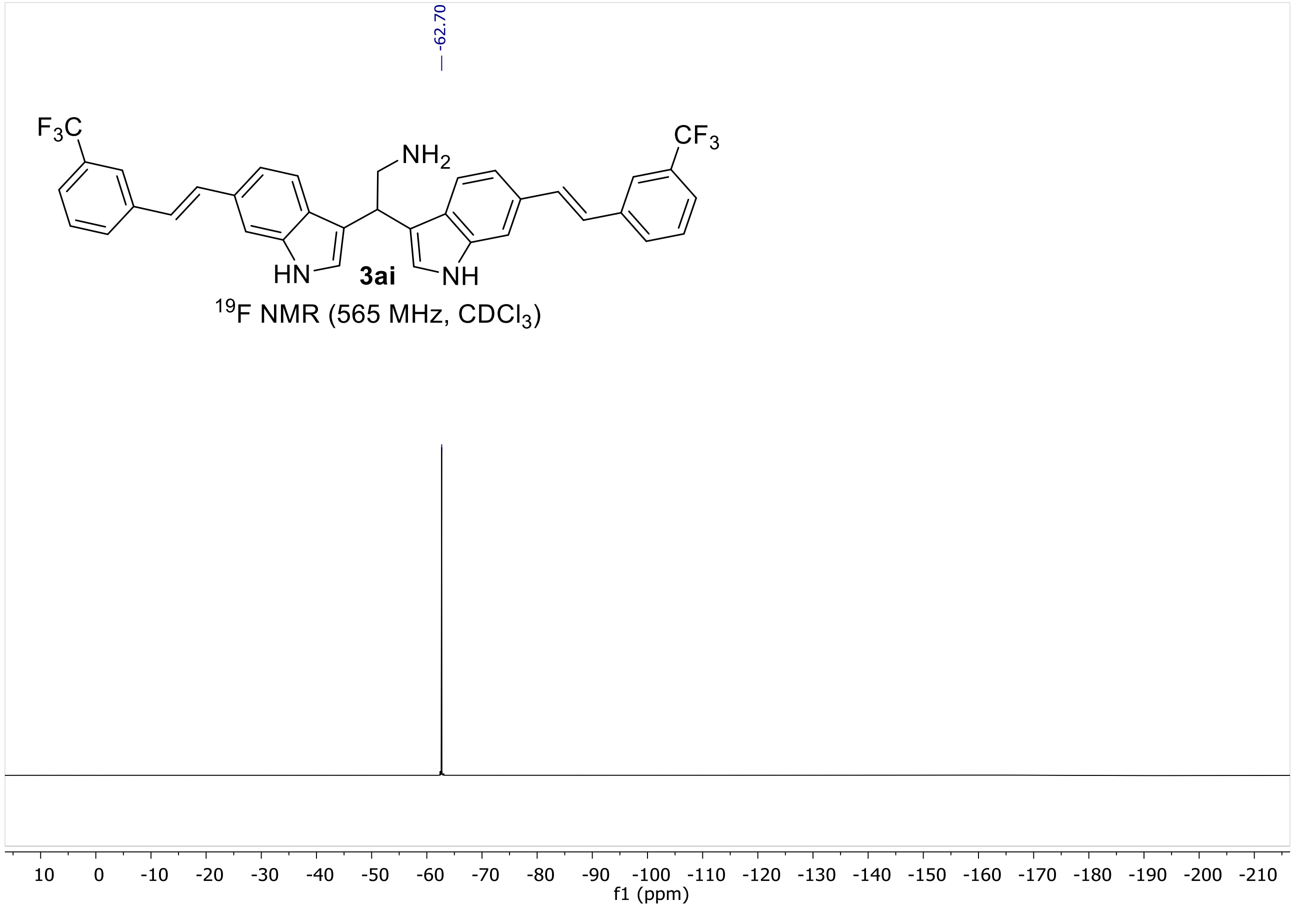
-62.70
F3C
NH2
CF3
HN
3ai
NH
19F NMR (565 MHz, CDCl3)
f1 (ppm)

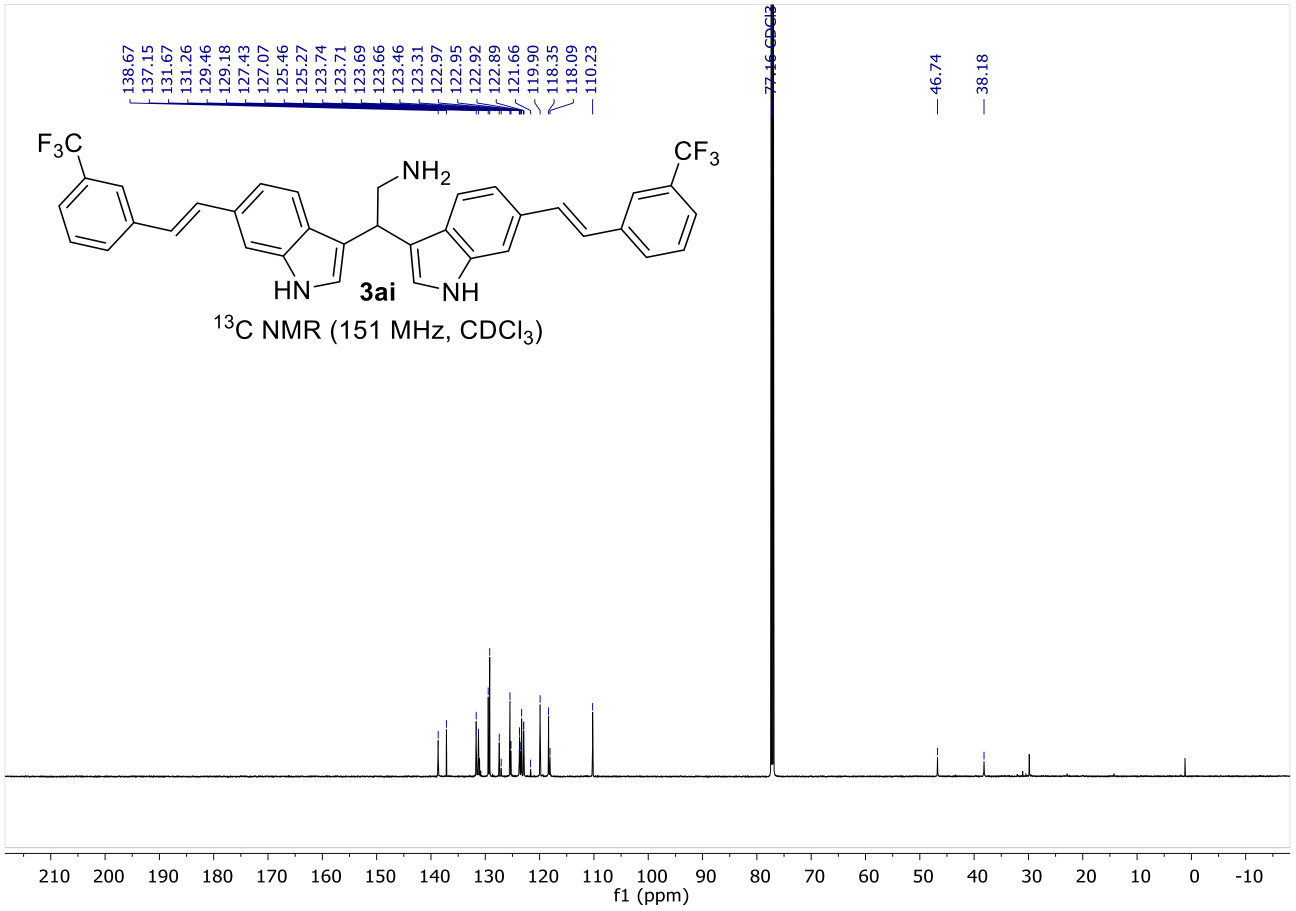
138.67
137.15
131.67
131.26
129.46
129.18
127.43
127.07
125.46
125.27
123.74
123.71
123.69
123.66
123.46
123.31
122.97
122.95
122.92
122.89
121.66
119.90
118.35
118.09
110.23
77.16 CDCl3
46.74
38.18
F3C
CF3
NH2
HN
NH
3ai
13C NMR (151 MHz, CDCl3)
210 200 190 180 170 160 150 140 130 120 110 100 90 80 70 60 50 40 30 20 10 0 -10
f1 (ppm)

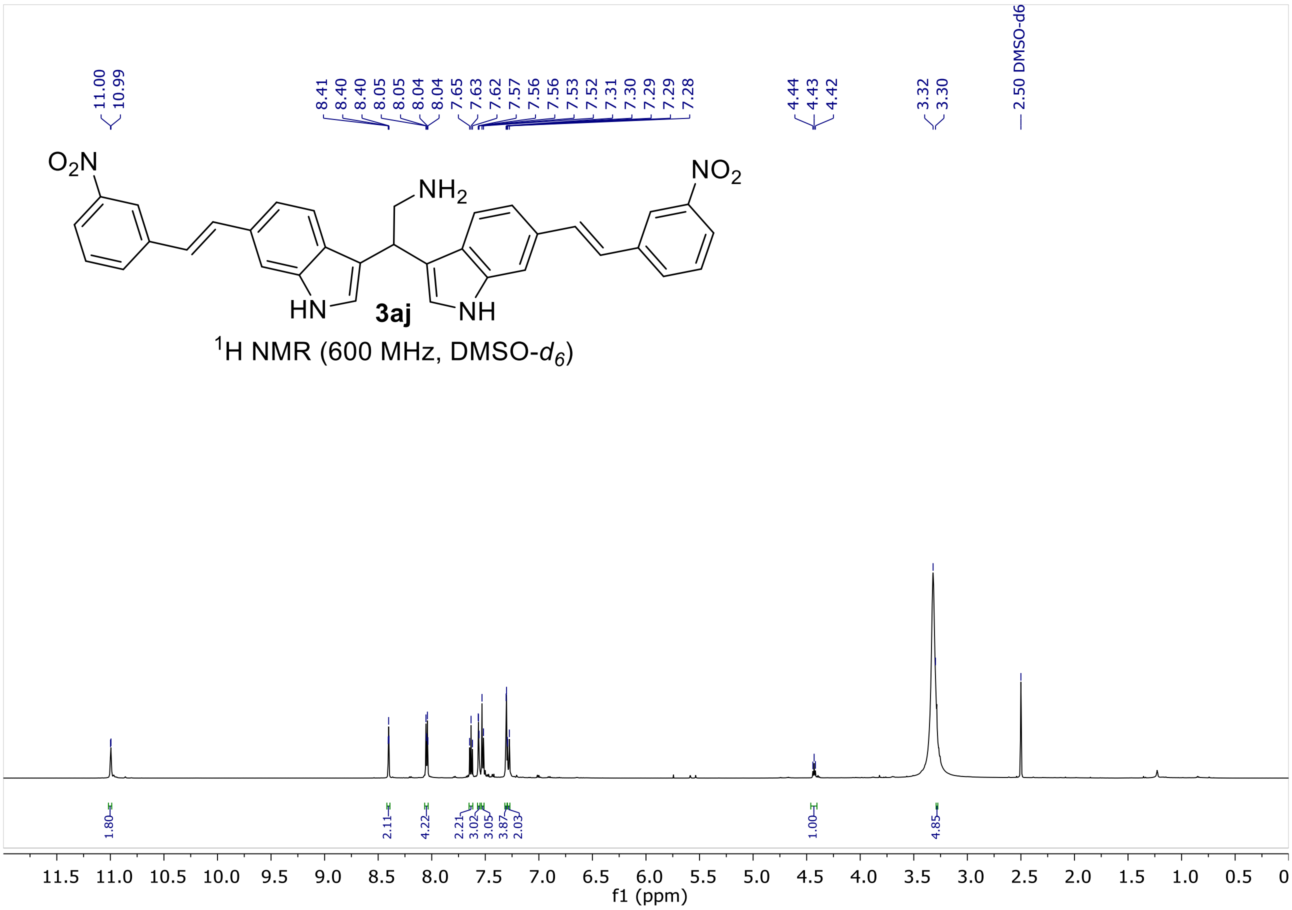
11.00
10.99
8.41
8.40
8.40
8.05
8.05
8.04
8.04
7.65
7.63
7.62
7.57
7.56
7.56
7.53
7.52
7.31
7.30
7.29
7.29
7.28
4.44
4.43
4.42
3.32
3.30
2.50 DMSO-d6
O2N
NH2
NO2
HN
3aj
NH
1H NMR (600 MHz, DMSO-d6)
1.80
2.11
4.22
2.21
3.02
3.05
3.87
2.03
1.00
4.85
f1 (ppm)

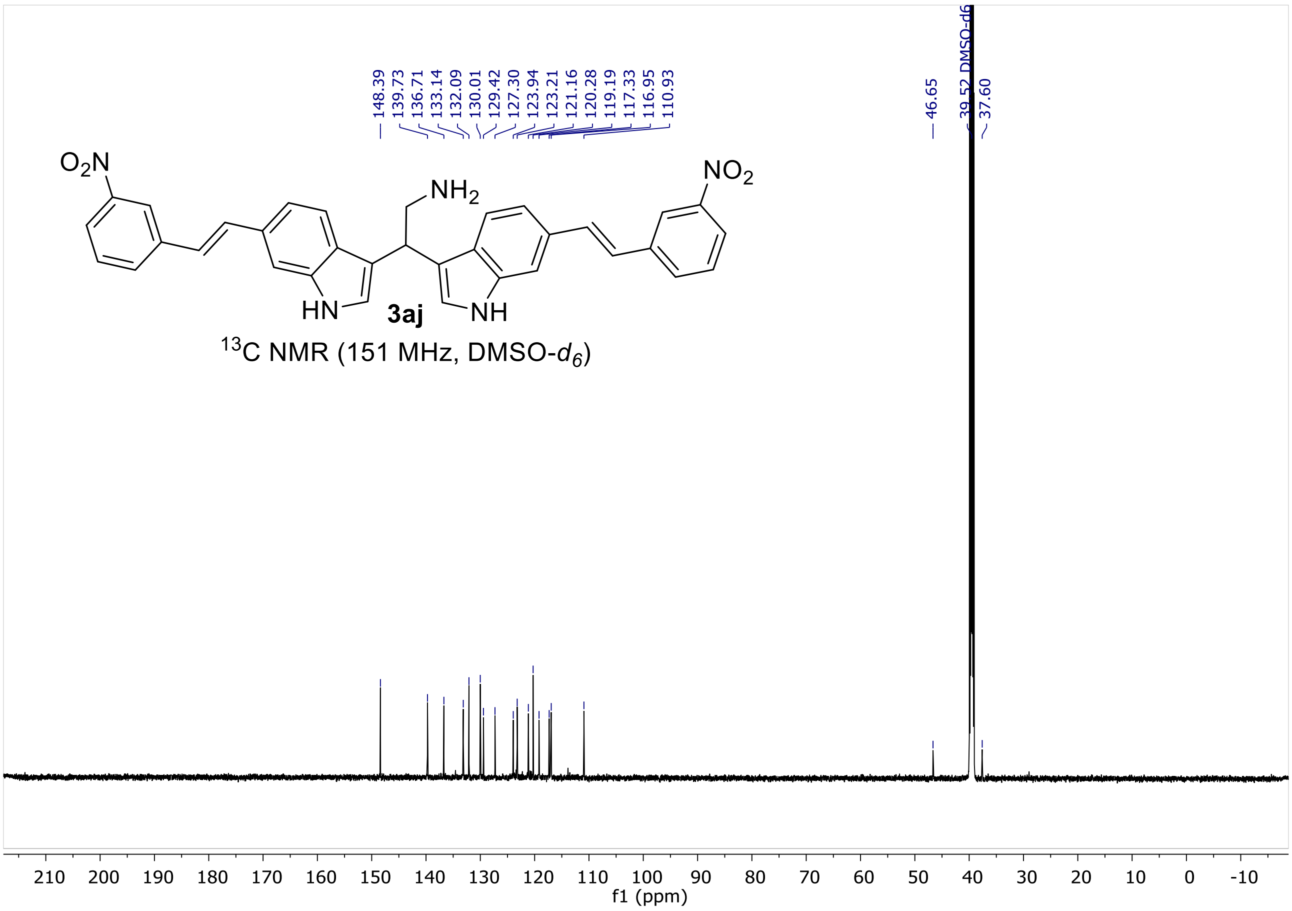
148.39
139.73
136.71
133.14
132.09
130.01
129.42
127.30
123.94
123.21
121.16
120.28
119.19
117.33
116.95
110.93
46.65
39.52 DMSO-d6
37.60
O2N
NH2
NO2
HN
3aj
NH
13C NMR (151 MHz, DMSO-d6)
f1 (ppm)

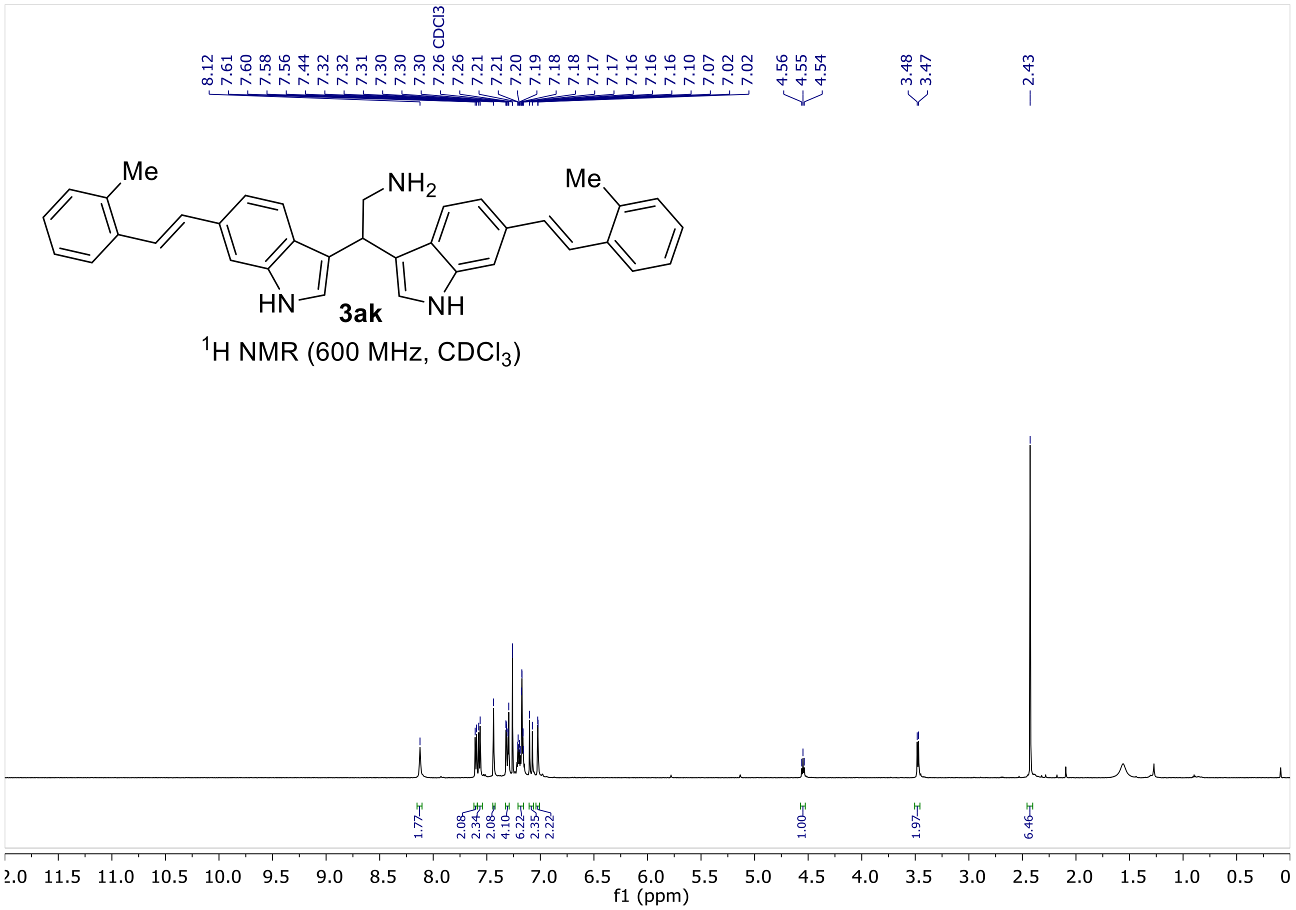
8.12 7.61 7.60 7.58 7.56 7.44 7.32 7.32 7.31 7.30 7.30 7.30 7.26 CDCl3 7.26 7.21 7.21 7.20 7.19 7.18 7.18 7.17 7.17 7.16 7.16 7.16 7.10 7.07 7.02 7.02 4.56 4.55 4.54 3.48 3.47 2.43
Me
NH2
Me
HN
3ak
NH
1H NMR (600 MHz, CDCl3)
1.77 2.08 2.34 2.08 4.10 6.22 2.35 2.22 1.00 1.97 6.46
f1 (ppm)

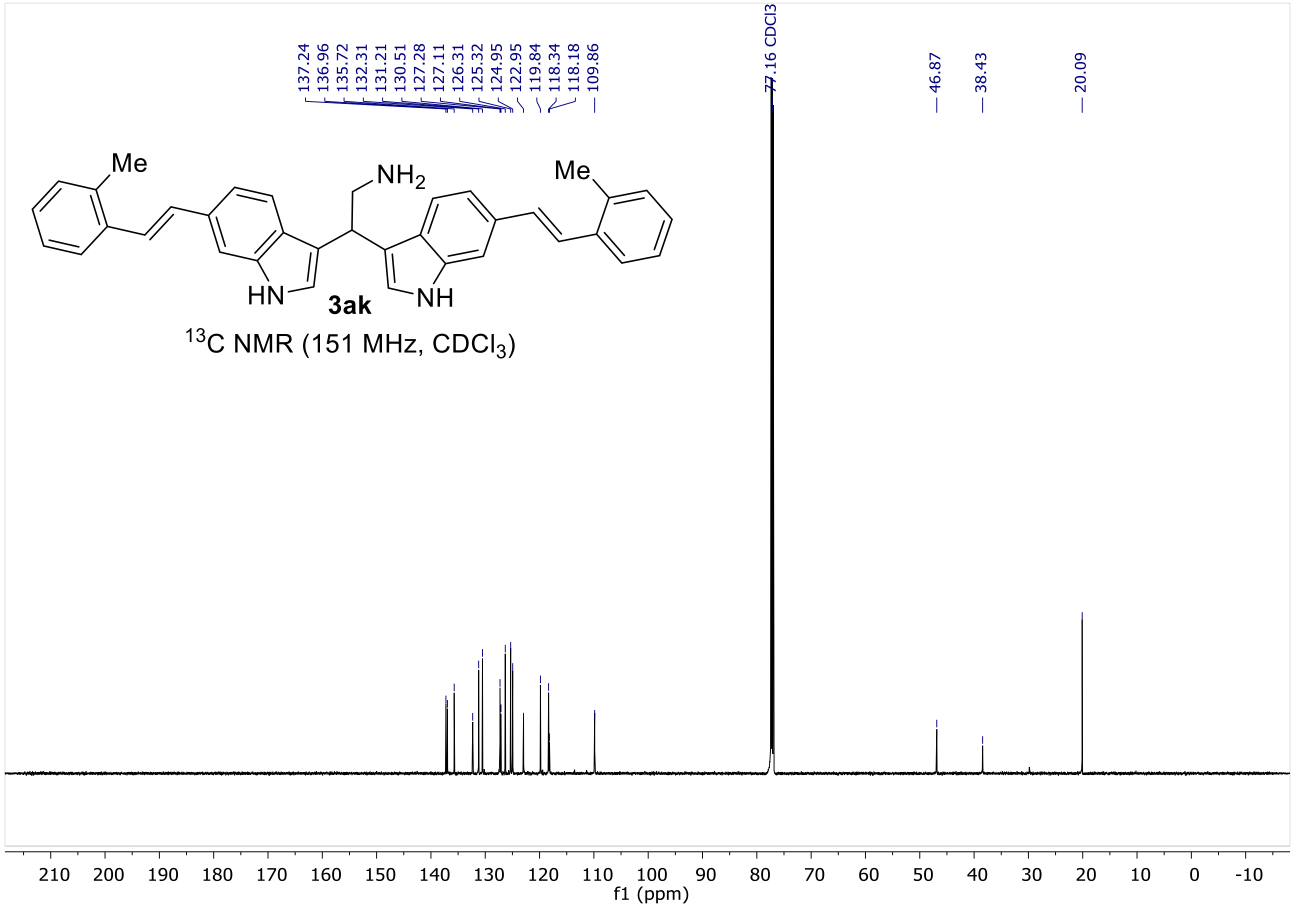
137.24 136.96 135.72 132.31 131.21 130.51 127.28 127.11 126.31 125.32 124.95 122.95 119.84 118.34 118.18 109.86 77.16 CDCl3 46.87 38.43 20.09
Me
NH2
Me
HN
3ak
NH
13C NMR (151 MHz, CDCl3)
f1 (ppm)

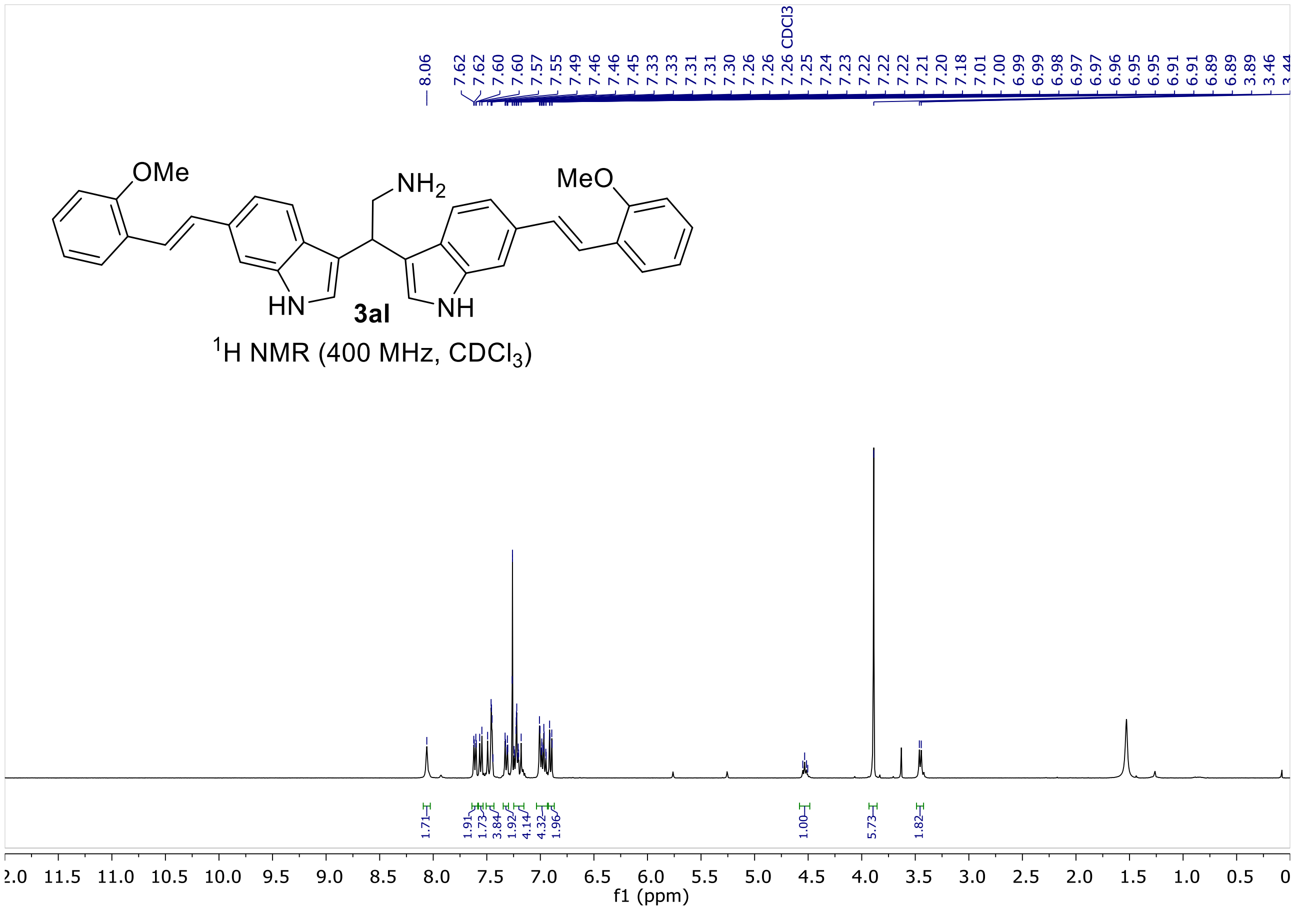
8.06
7.62 7.62 7.60 7.60 7.57 7.55 7.49 7.46 7.46 7.45 7.33 7.33 7.31 7.31 7.30 7.26 7.26 CDCl3 7.25 7.24 7.23 7.22 7.22 7.22 7.21 7.20 7.18 7.01 7.00 6.99 6.99 6.98 6.97 6.96 6.95 6.95 6.91 6.91 6.89 6.89 3.89 3.46
OMe
NH2
MeO
HN
3al
NH
1H NMR (400 MHz, CDCl3)
1.71
1.91
1.73
3.84
1.92
4.14
4.32
1.96
1.00
5.73
1.82
f1 (ppm)

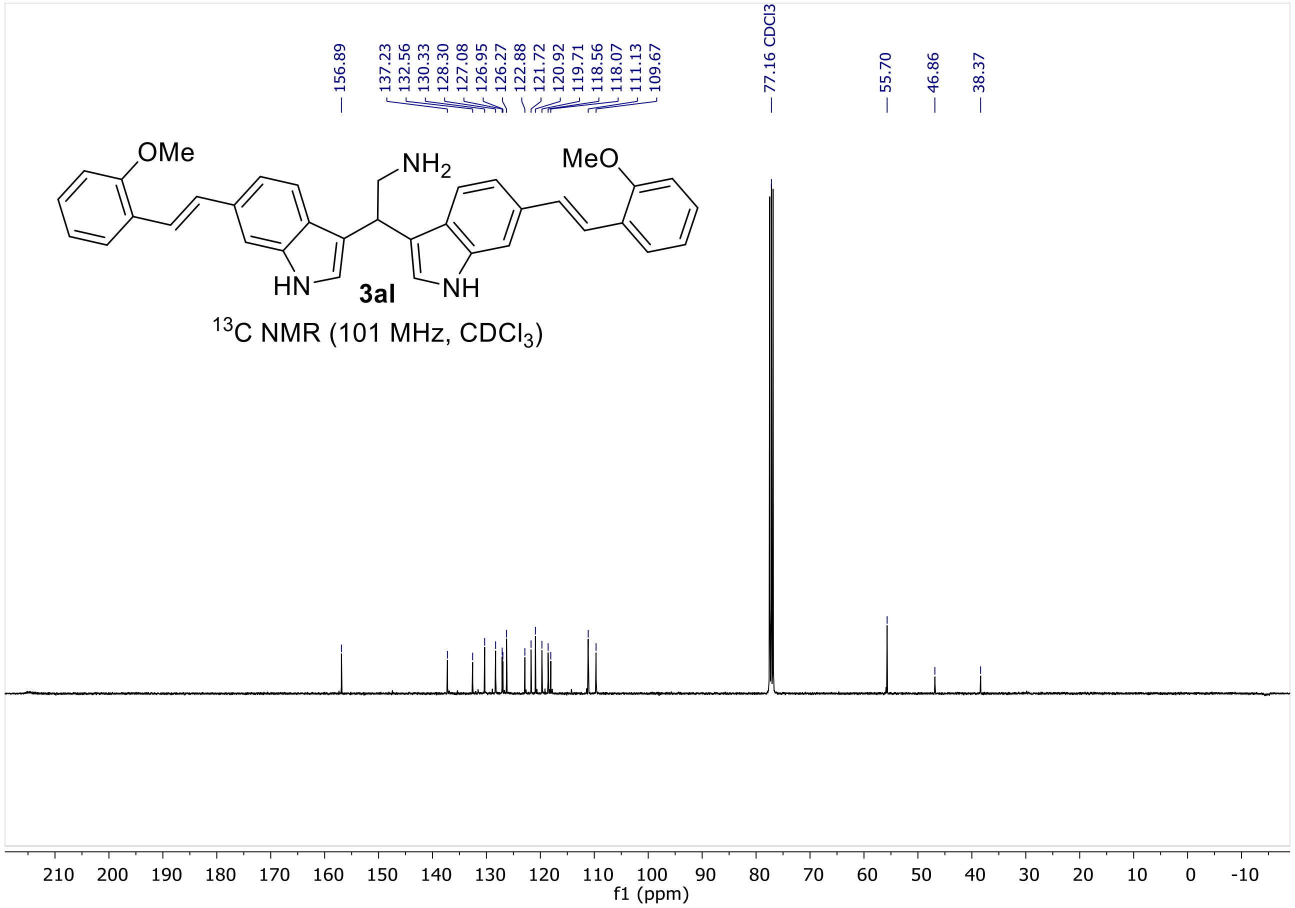
156.89
137.23 132.56 130.33 128.30 127.08 126.95 126.27 122.88 121.72 120.92 119.71 118.56 118.07 111.13 109.67
77.16 CDCl3
55.70
46.86
38.37
OMe
NH2
MeO
HN
3al
NH
13C NMR (101 MHz, CDCl3)
f1 (ppm)

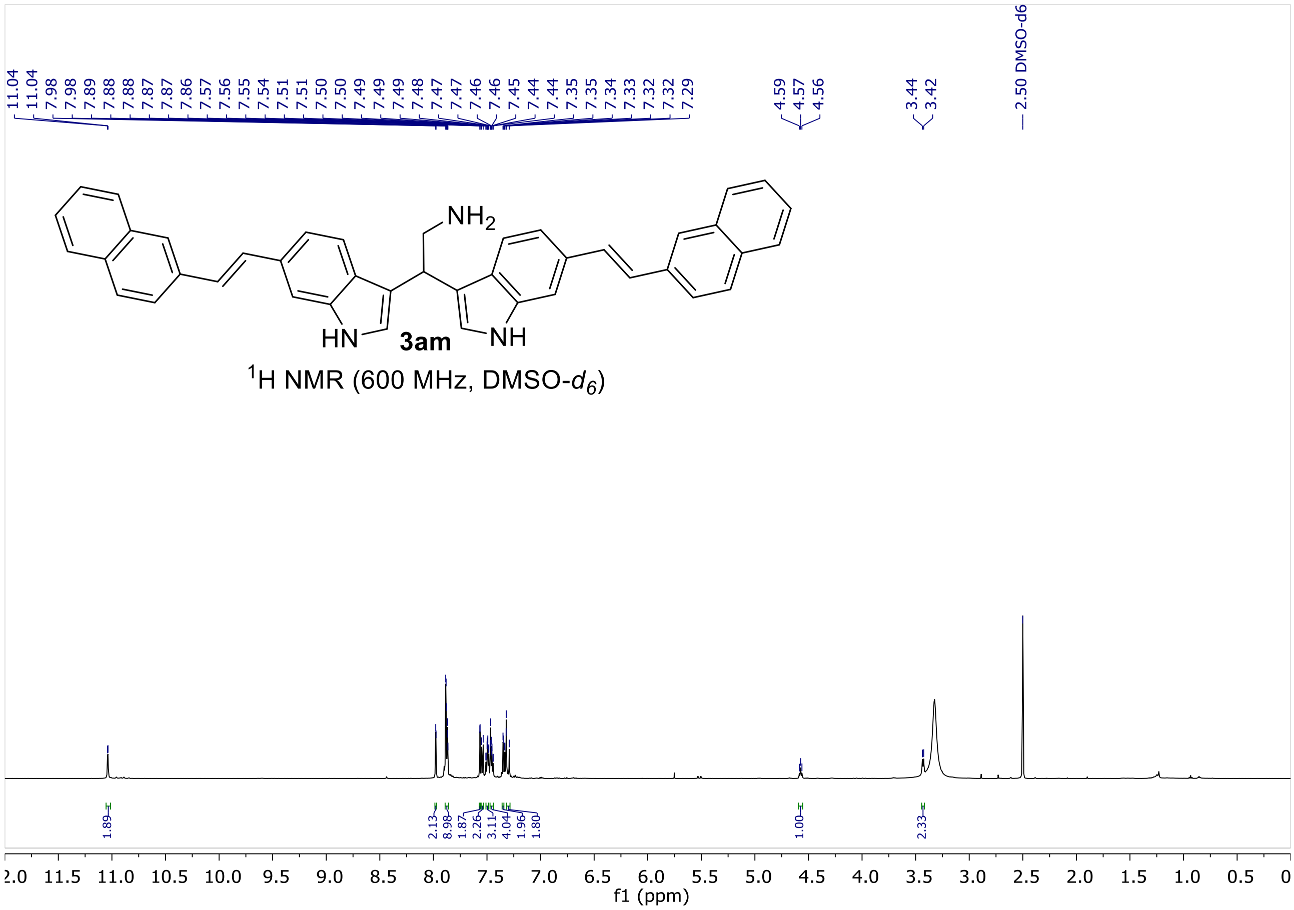
11.04
11.04
7.98
7.98
7.89
7.88
7.88
7.87
7.87
7.86
7.57
7.56
7.55
7.54
7.51
7.51
7.50
7.50
7.49
7.49
7.49
7.48
7.47
7.47
7.46
7.46
7.45
7.44
7.44
7.35
7.35
7.34
7.33
7.32
7.32
7.29
4.59
4.57
4.56
3.44
3.42
2.50 DMSO-d6
NH2
HN
3am
NH
1H NMR (600 MHz, DMSO-d6)
1.89
2.13
8.98
1.87
2.26
3.11
4.04
1.96
1.80
1.00
2.33
f1 (ppm)

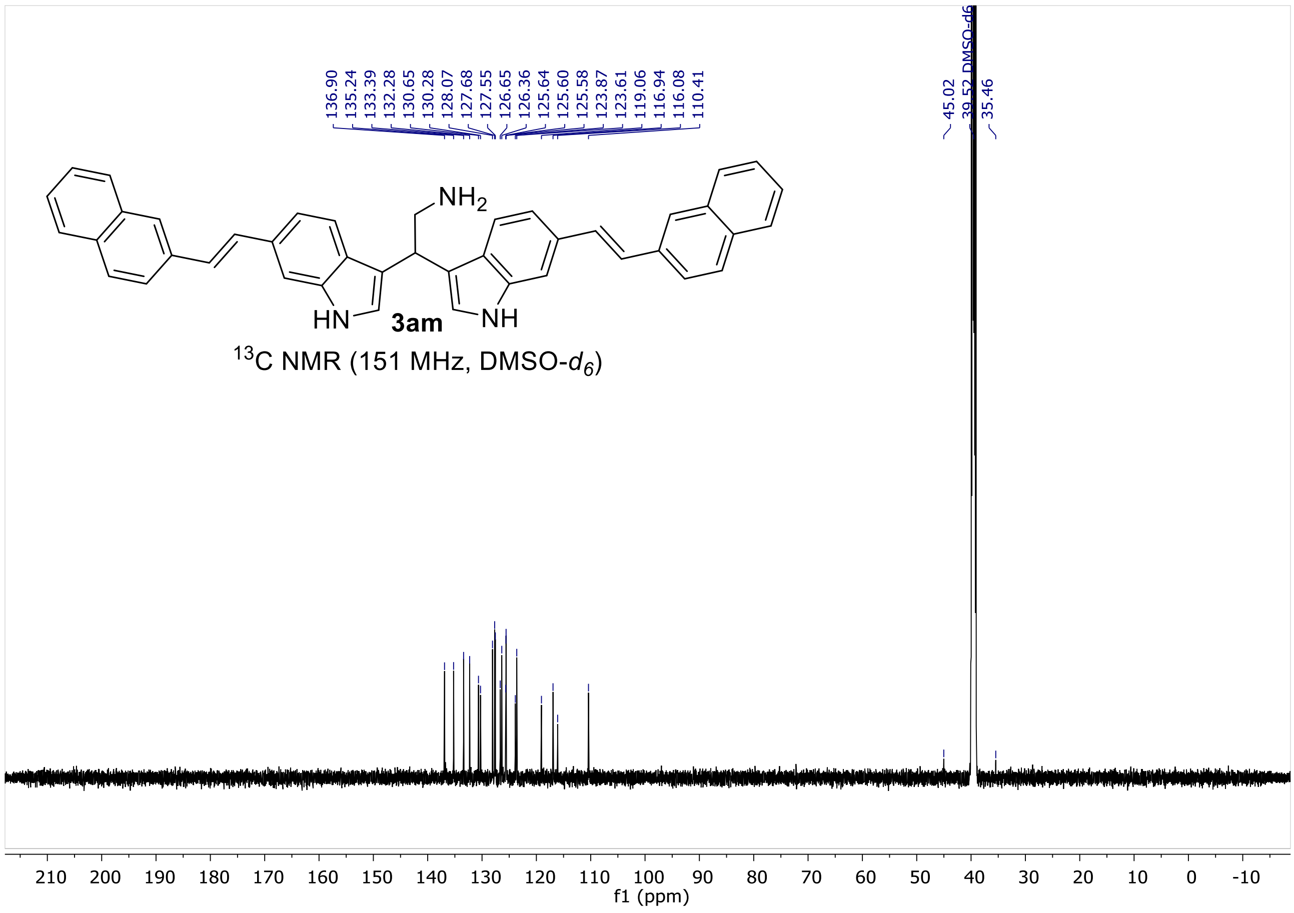
136.90
135.24
133.39
132.28
130.65
130.28
128.07
127.68
127.55
126.65
126.36
125.64
125.60
125.58
123.87
123.61
119.06
116.94
116.08
110.41
45.02
39.52 DMSO-d6
35.46
NH2
HN
3am
NH
13C NMR (151 MHz, DMSO-d6)
f1 (ppm)

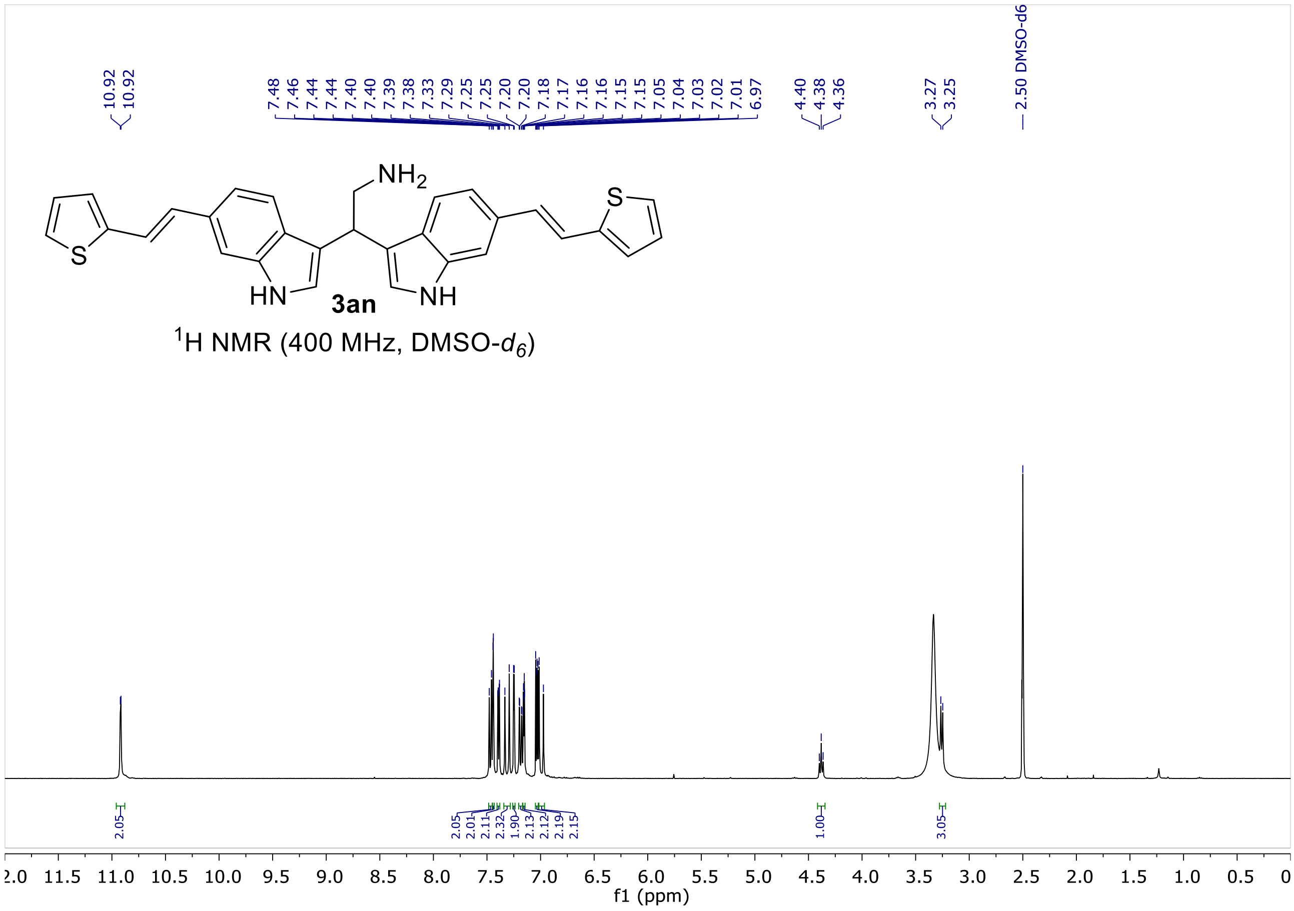
10.92
10.92
7.48
7.46
7.44
7.44
7.40
7.40
7.39
7.38
7.33
7.29
7.25
7.25
7.20
7.20
7.18
7.17
7.16
7.15
7.15
7.05
7.04
7.03
7.02
7.01
6.97
4.40
4.38
4.36
3.27
3.25
2.50 DMSO-d6
NH2
S
S
HN
3an
NH
1H NMR (400 MHz, DMSO-d6)
2.05
2.05
2.01
2.11
2.32
1.90
2.13
2.12
2.19
2.15
1.00
3.05
f1 (ppm)

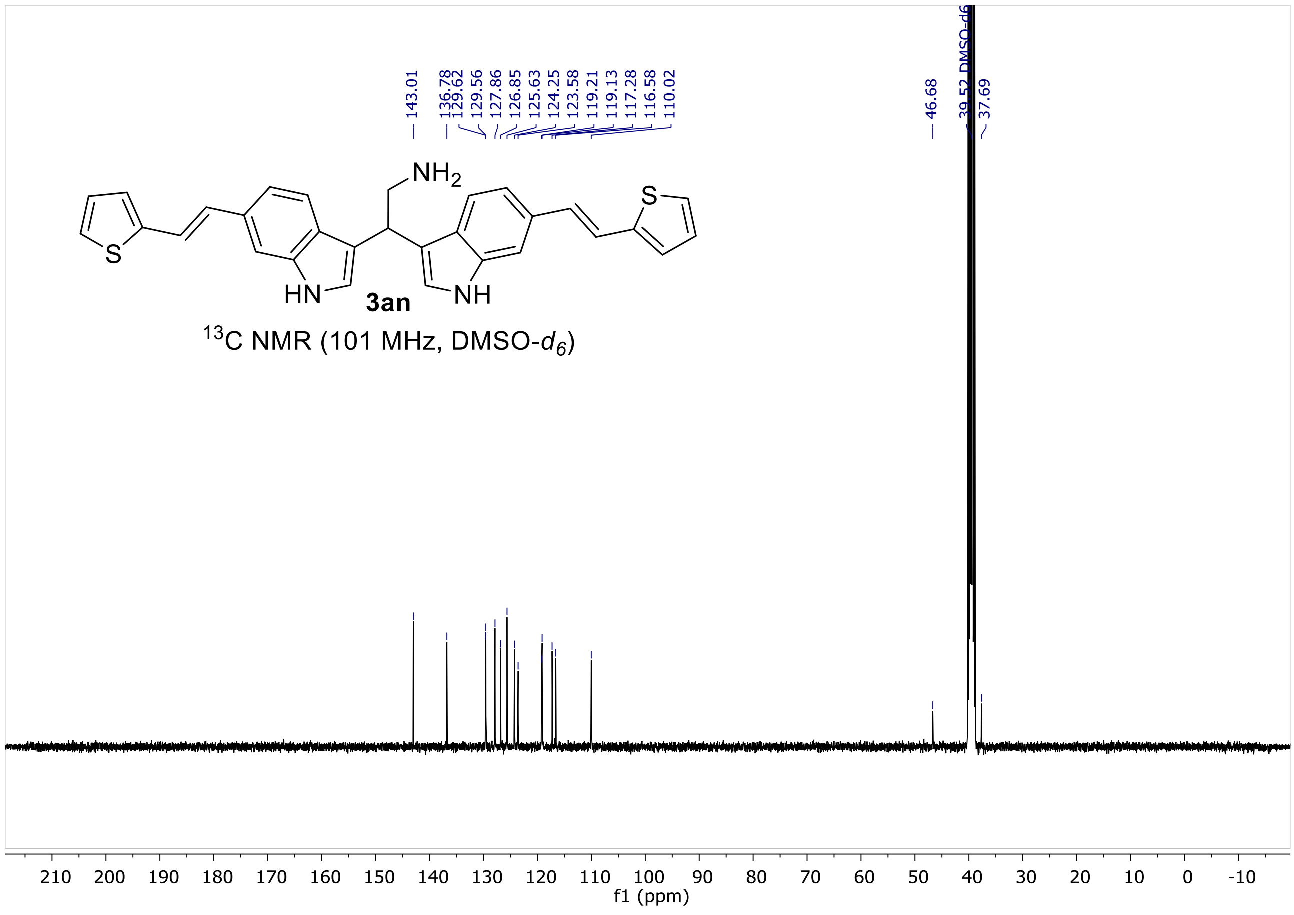
143.01
136.78
129.62
129.56
127.86
126.85
125.63
124.25
123.58
119.21
119.13
117.28
116.58
110.02
46.68
39.52 DMSO-d6
37.69
NH2
S
S
HN
3an
NH
13C NMR (101 MHz, DMSO-d6)
f1 (ppm)

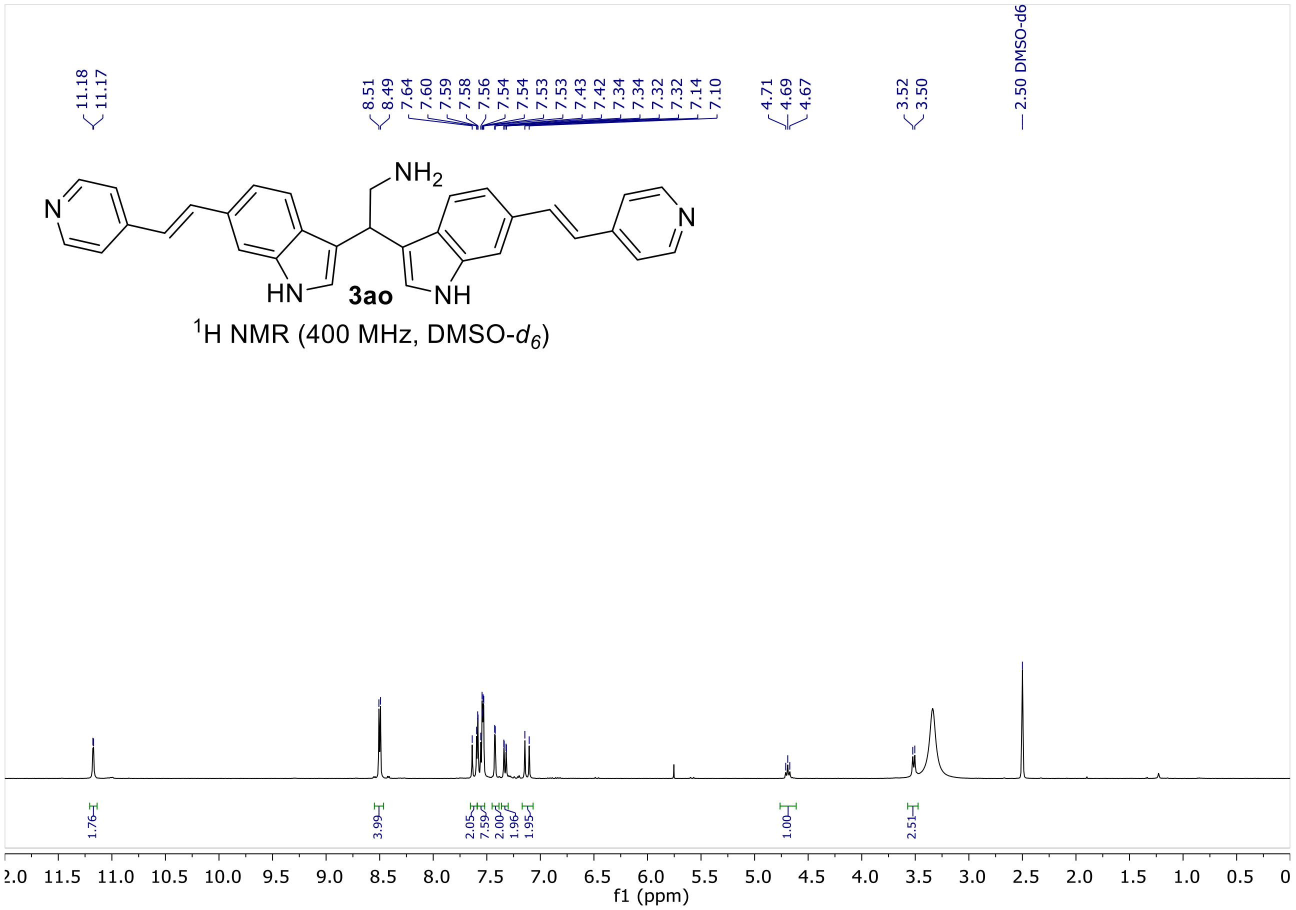
11.18
11.17
8.51
8.49
7.64
7.60
7.59
7.58
7.56
7.54
7.54
7.53
7.53
7.43
7.42
7.34
7.34
7.32
7.32
7.14
7.10
4.71
4.69
4.67
3.52
3.50
2.50 DMSO-d6
NH2
N
HN
3ao
NH
N
1H NMR (400 MHz, DMSO-d6)
1.76
3.99
2.05
7.59
2.00
1.96
1.95
1.00
2.51
f1 (ppm)

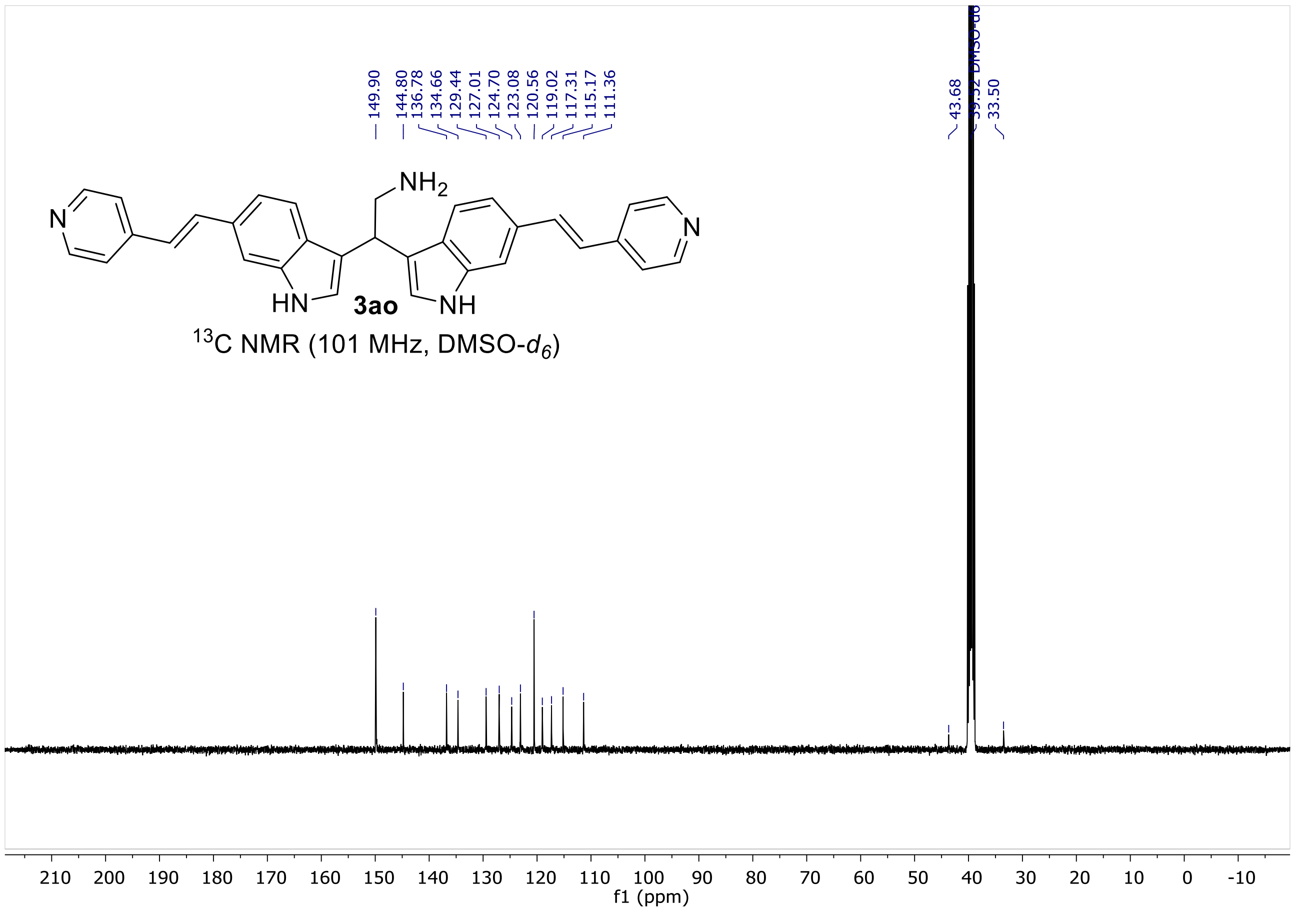
149.90
144.80
136.78
134.66
129.44
127.01
124.70
123.08
120.56
119.02
117.31
115.17
111.36
43.68
39.52
33.50
NH2
N
HN
3ao
NH
N
13C NMR (101 MHz, DMSO-d6)
f1 (ppm)

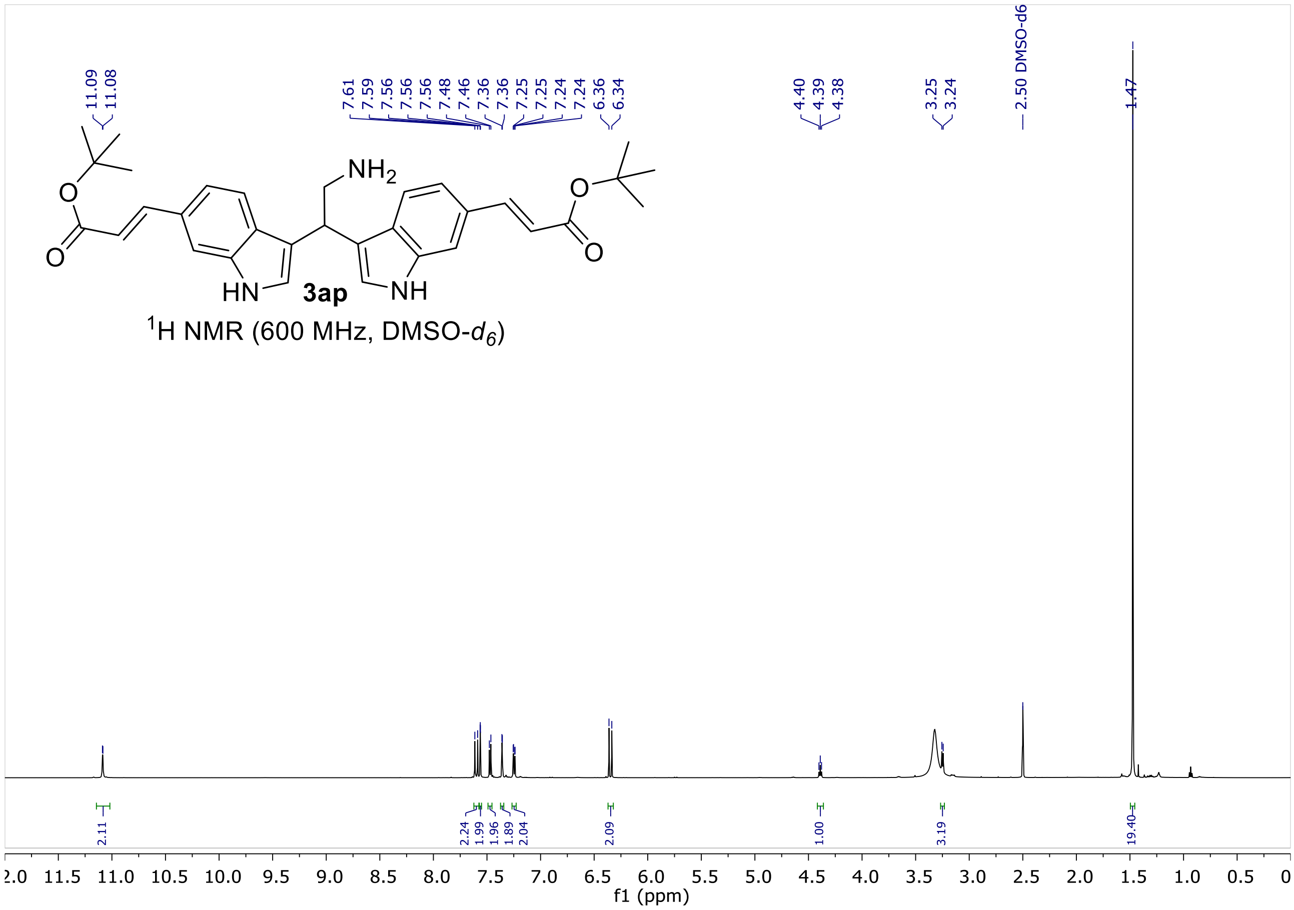
11.09
11.08
7.61
7.59
7.56
7.56
7.56
7.48
7.46
7.36
7.36
7.25
7.25
7.24
7.24
6.36
6.34
4.40
4.39
4.38
3.25
3.24
2.50 DMSO-d6
1.47
NH2
O
O
O
O
HN
NH
3ap
1H NMR (600 MHz, DMSO-d6)
2.11
2.24
1.99
1.96
1.89
2.04
2.09
1.00
3.19
19.40
f1 (ppm)

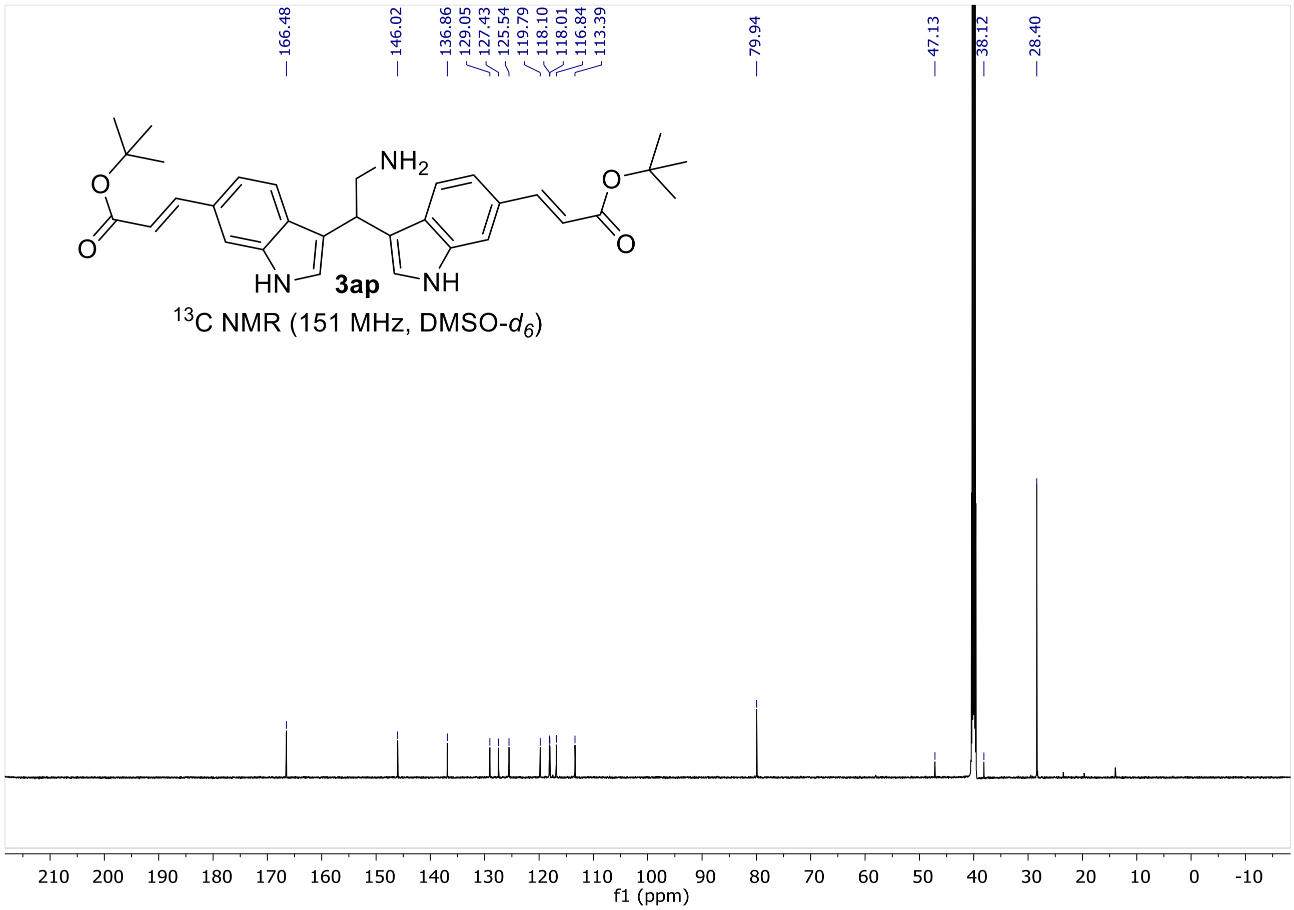
166.48
146.02
136.86
129.05
127.43
125.54
119.79
118.10
118.01
116.84
113.39
79.94
47.13
38.12
28.40
NH2
O
O
O
O
HN
NH
3ap
13C NMR (151 MHz, DMSO-d6)
f1 (ppm)

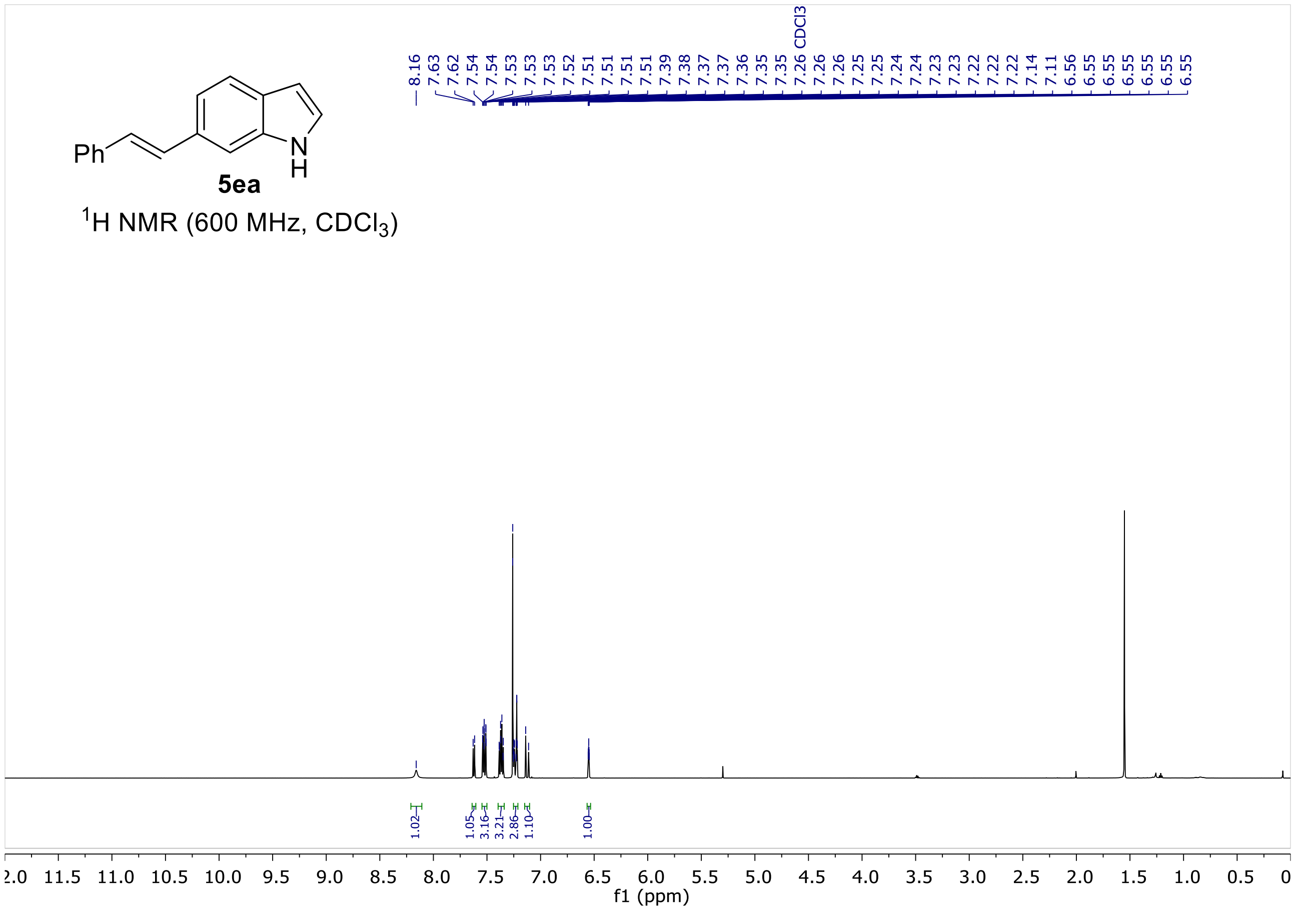
Ph
N
H
5ea
$^{1}$H NMR (600 MHz, CDCl3)
f1 (ppm)

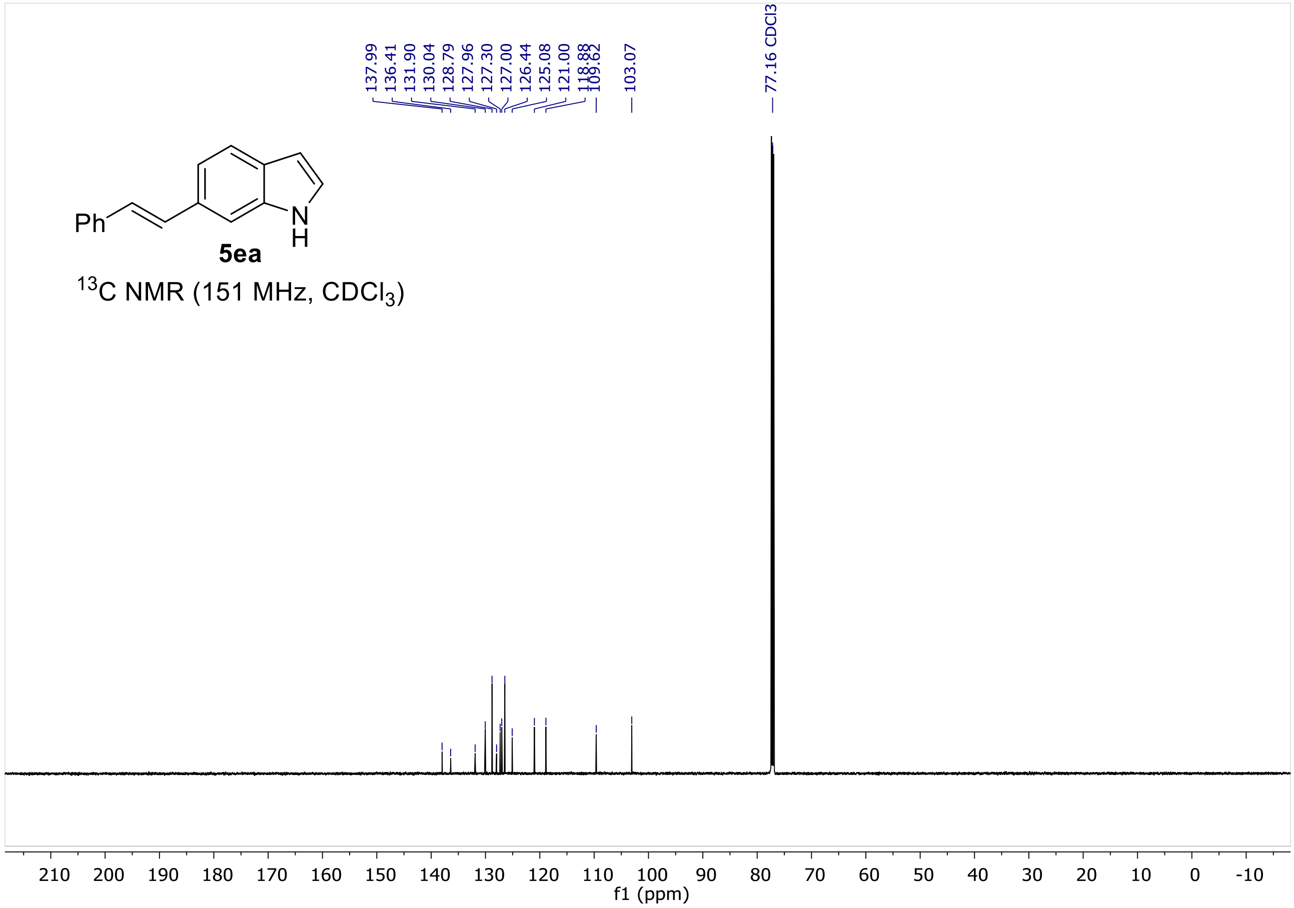
Ph
N
H
5ea
$^{13}$C NMR (151 MHz, CDCl3)
77.16 CDCl3
f1 (ppm)

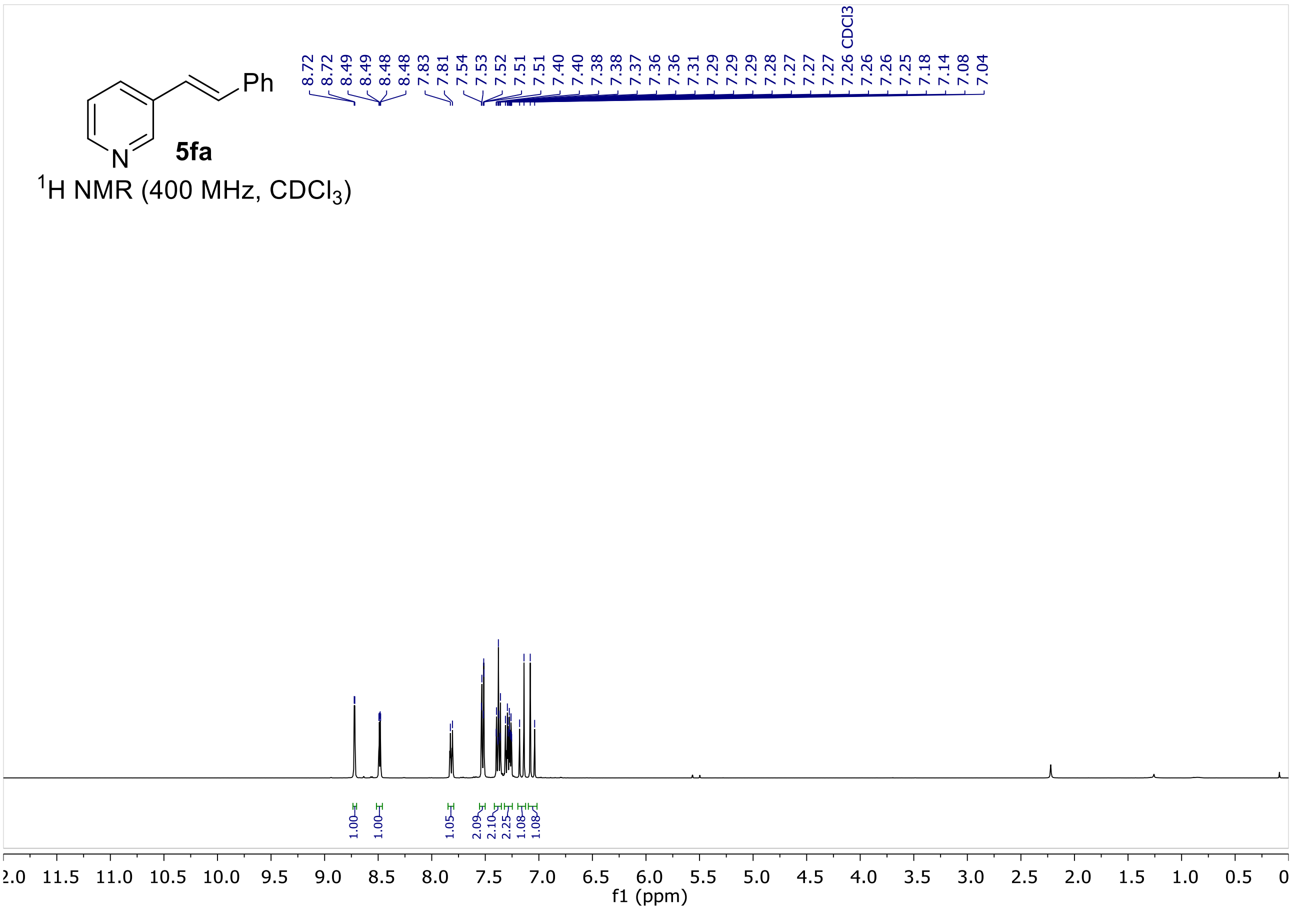
Ph
N
5fa
1H NMR (400 MHz, CDCl3)
8.72 8.72 8.49 8.49 8.48 8.48 7.83 7.81 7.54 7.53 7.52 7.51 7.51 7.40 7.40 7.38 7.38 7.37 7.36 7.36 7.31 7.29 7.29 7.29 7.28 7.27 7.27 7.27 7.26 CDCl3 7.26 7.26 7.25 7.18 7.14 7.08 7.04
1.00 1.00 1.05 2.09 2.10 2.25 1.08 1.08
2.0 11.5 11.0 10.5 10.0 9.5 9.0 8.5 8.0 7.5 7.0 6.5 6.0 5.5 5.0 4.5 4.0 3.5 3.0 2.5 2.0 1.5 1.0 0.5 0
f1 (ppm)

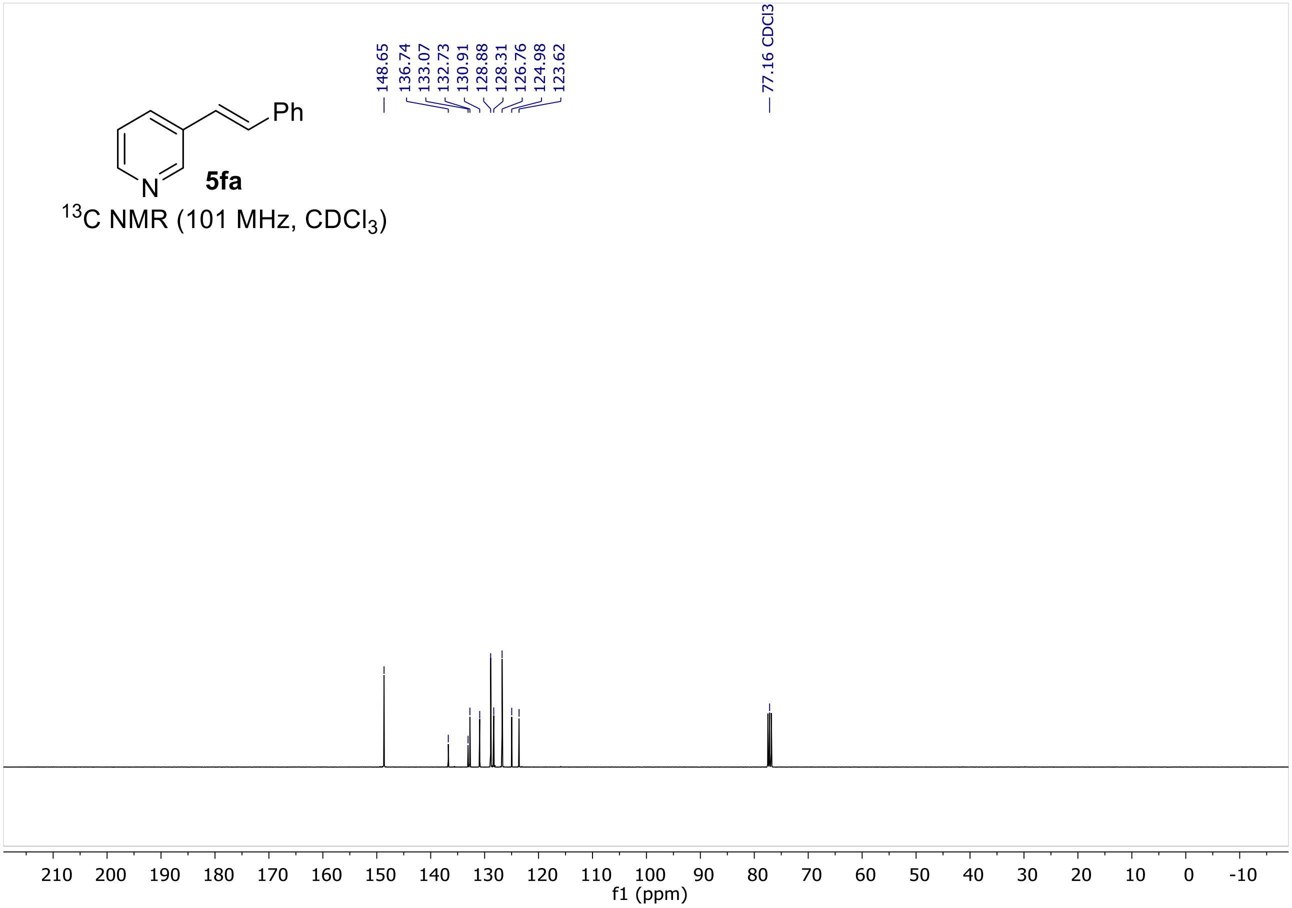
Ph
N
5fa
13C NMR (101 MHz, CDCl3)
148.65 136.74 133.07 132.73 130.91 128.88 128.31 126.76 124.98 123.62
77.16 CDCl3
210 200 190 180 170 160 150 140 130 120 110 100 90 80 70 60 50 40 30 20 10 0 -10
f1 (ppm)

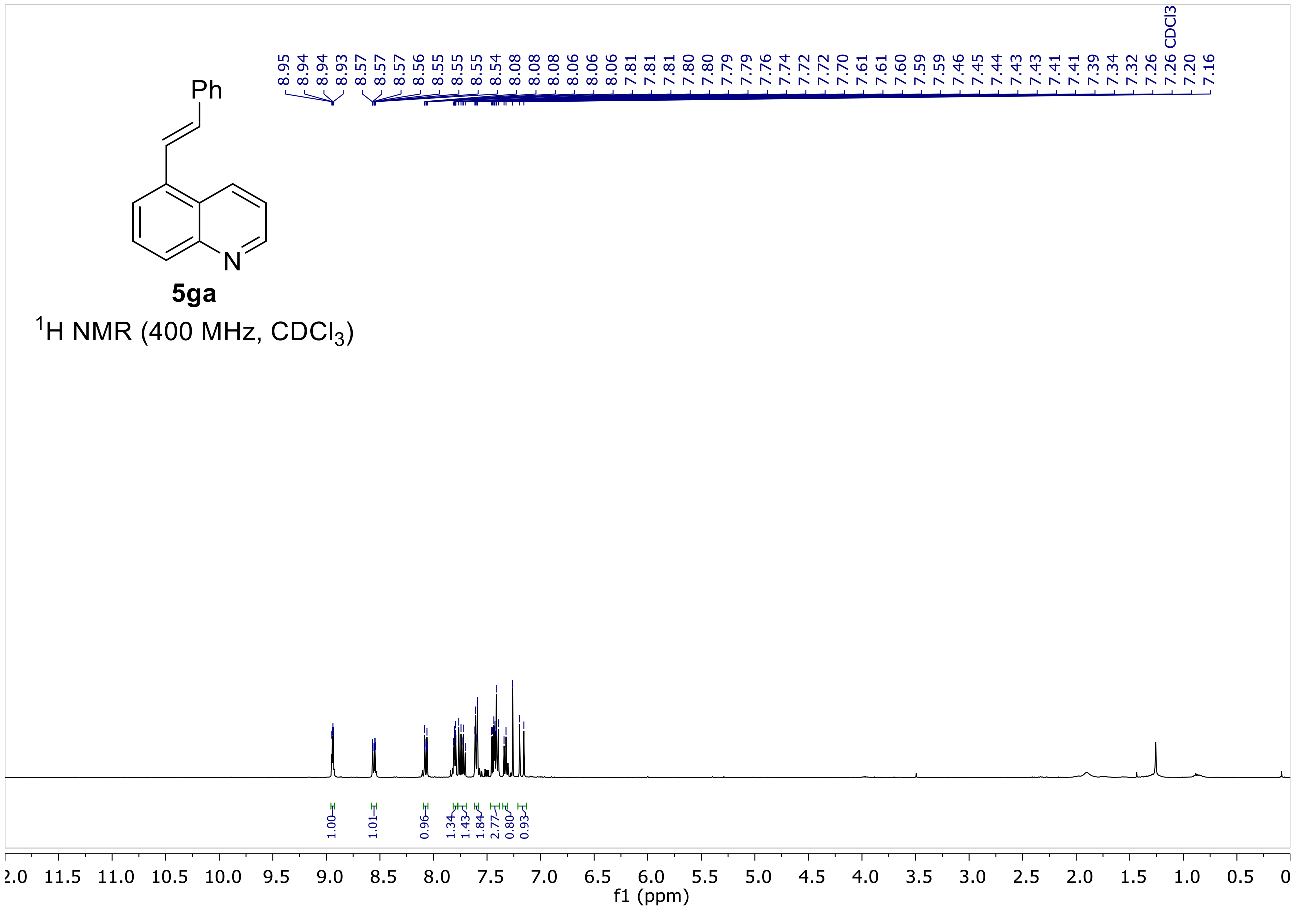
Ph
N
5ga
1H NMR (400 MHz, CDCl3)
8.95 8.94 8.94 8.93 8.57 8.57 8.57 8.56 8.55 8.55 8.55 8.54 8.08 8.08 8.08 8.06 8.06 8.06 7.81 7.81 7.81 7.80 7.80 7.79 7.79 7.76 7.74 7.72 7.72 7.70 7.61 7.61 7.60 7.59 7.59 7.46 7.45 7.44 7.43 7.43 7.41 7.41 7.39 7.34 7.32 7.26 7.26 CDCl3 7.20 7.16
1.00 1.01 0.96 1.34 1.43 1.84 2.77 0.80 0.93
f1 (ppm)

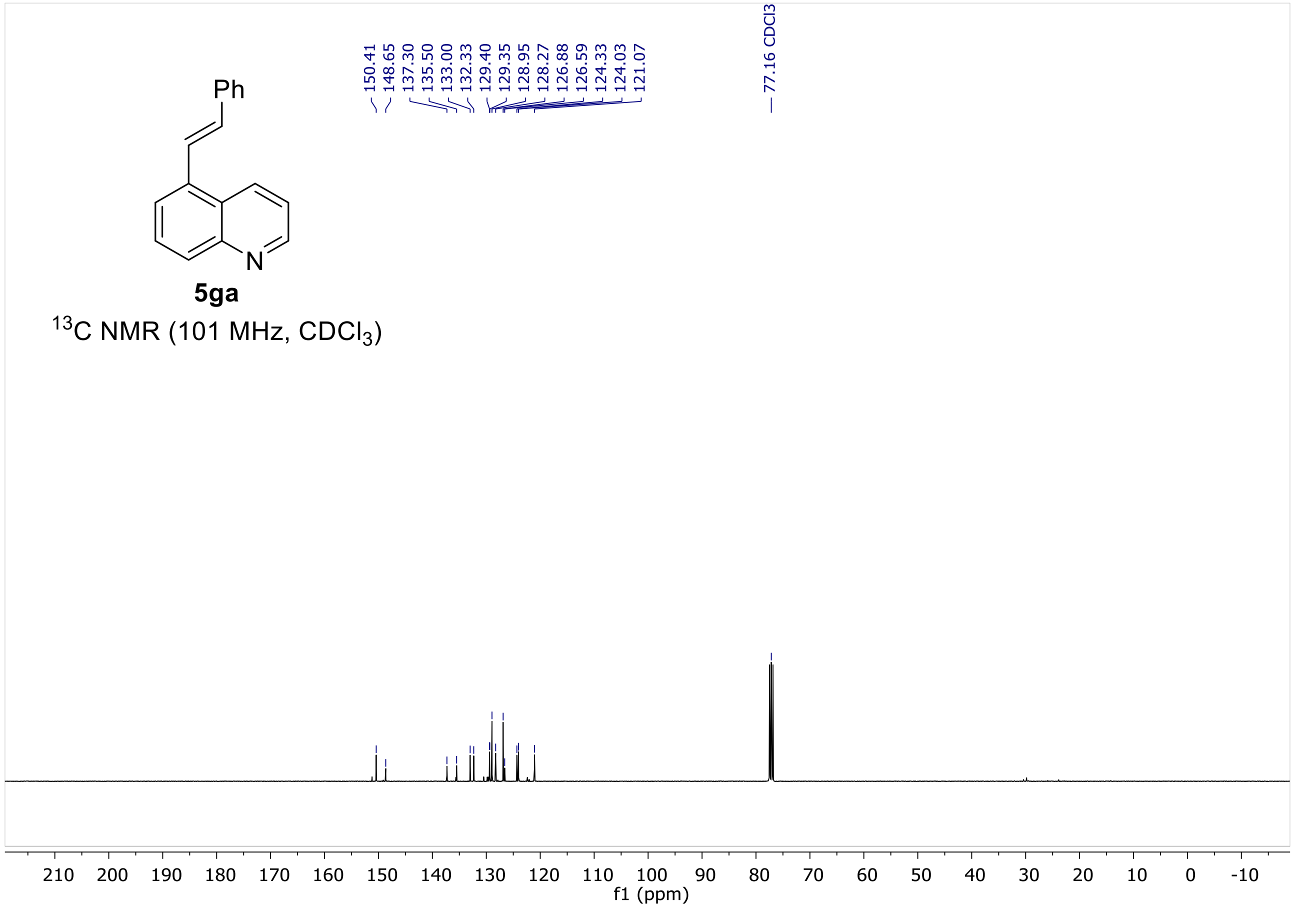
Ph
N
5ga
13C NMR (101 MHz, CDCl3)
150.41 148.65 137.30 135.50 133.00 132.33 129.40 129.35 128.95 128.27 126.88 126.59 124.33 124.03 121.07
77.16 CDCl3
f1 (ppm)

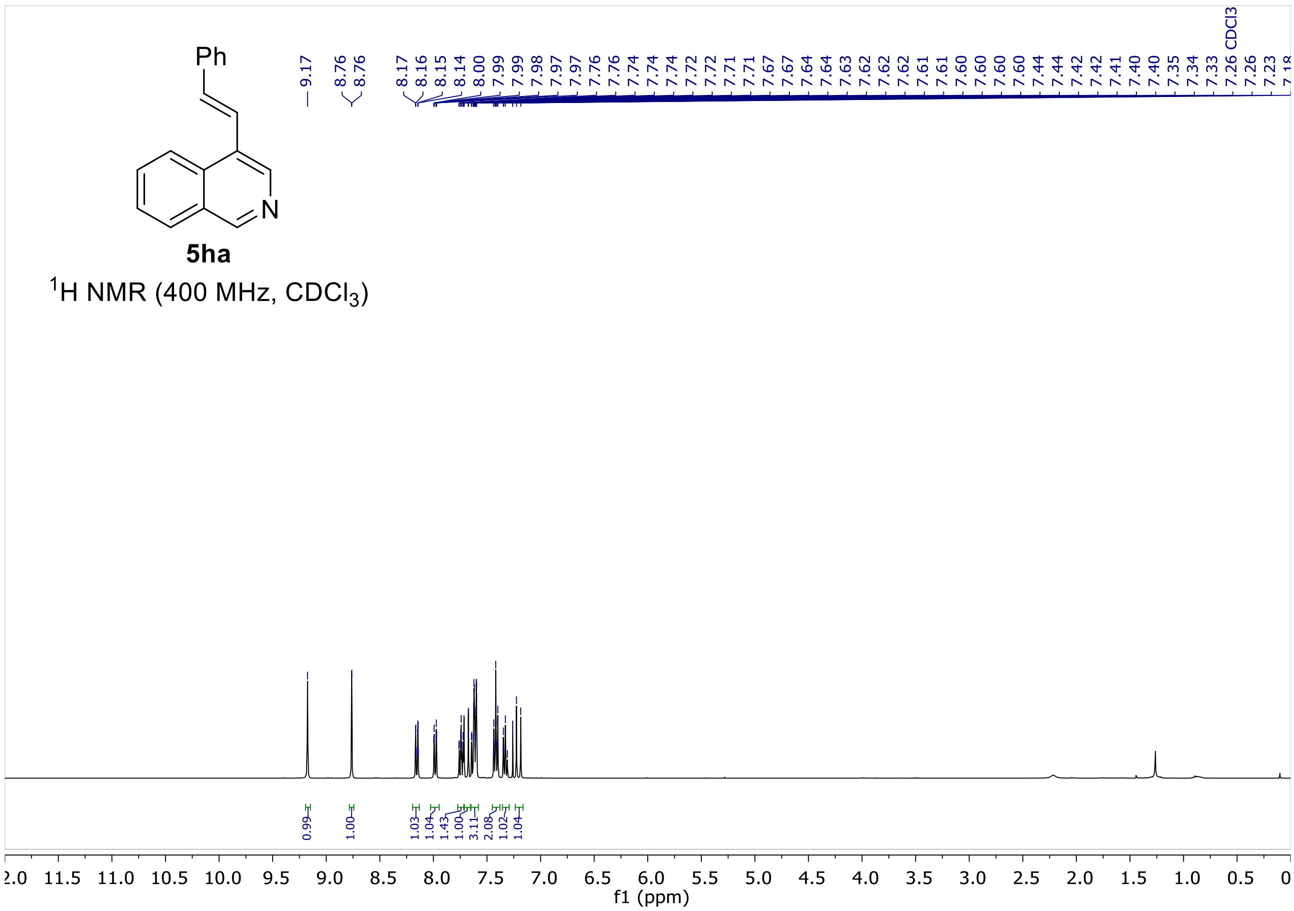
Ph
N
5ha
1H NMR (400 MHz, CDCl3)
9.17
8.76
8.76
8.17
8.16
8.15
8.14
8.00
7.99
7.99
7.98
7.97
7.97
7.76
7.76
7.74
7.74
7.74
7.72
7.72
7.71
7.71
7.67
7.67
7.64
7.64
7.63
7.62
7.62
7.62
7.61
7.61
7.60
7.60
7.60
7.60
7.44
7.44
7.42
7.41
7.40
7.40
7.35
7.34
7.33
7.26 CDCl3
7.26
7.23
0.99
1.00
1.03
1.04
1.43
1.00
3.11
2.08
1.02
1.04
f1 (ppm)

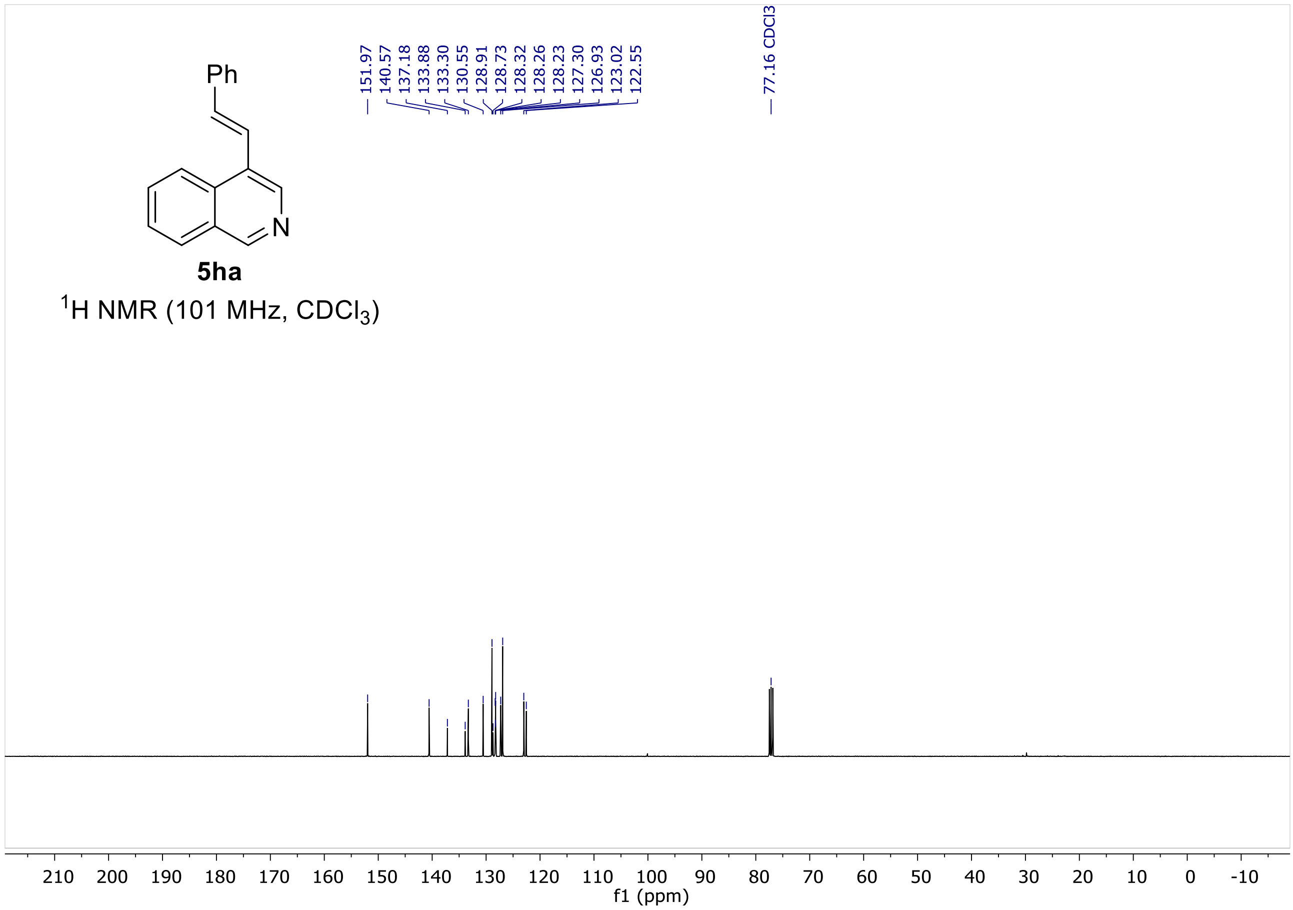
Ph
N
5ha
1H NMR (101 MHz, CDCl3)
151.97
140.57
137.18
133.88
133.30
130.55
128.91
128.73
128.32
128.26
128.23
127.30
126.93
123.02
122.55
77.16 CDCl3
f1 (ppm)

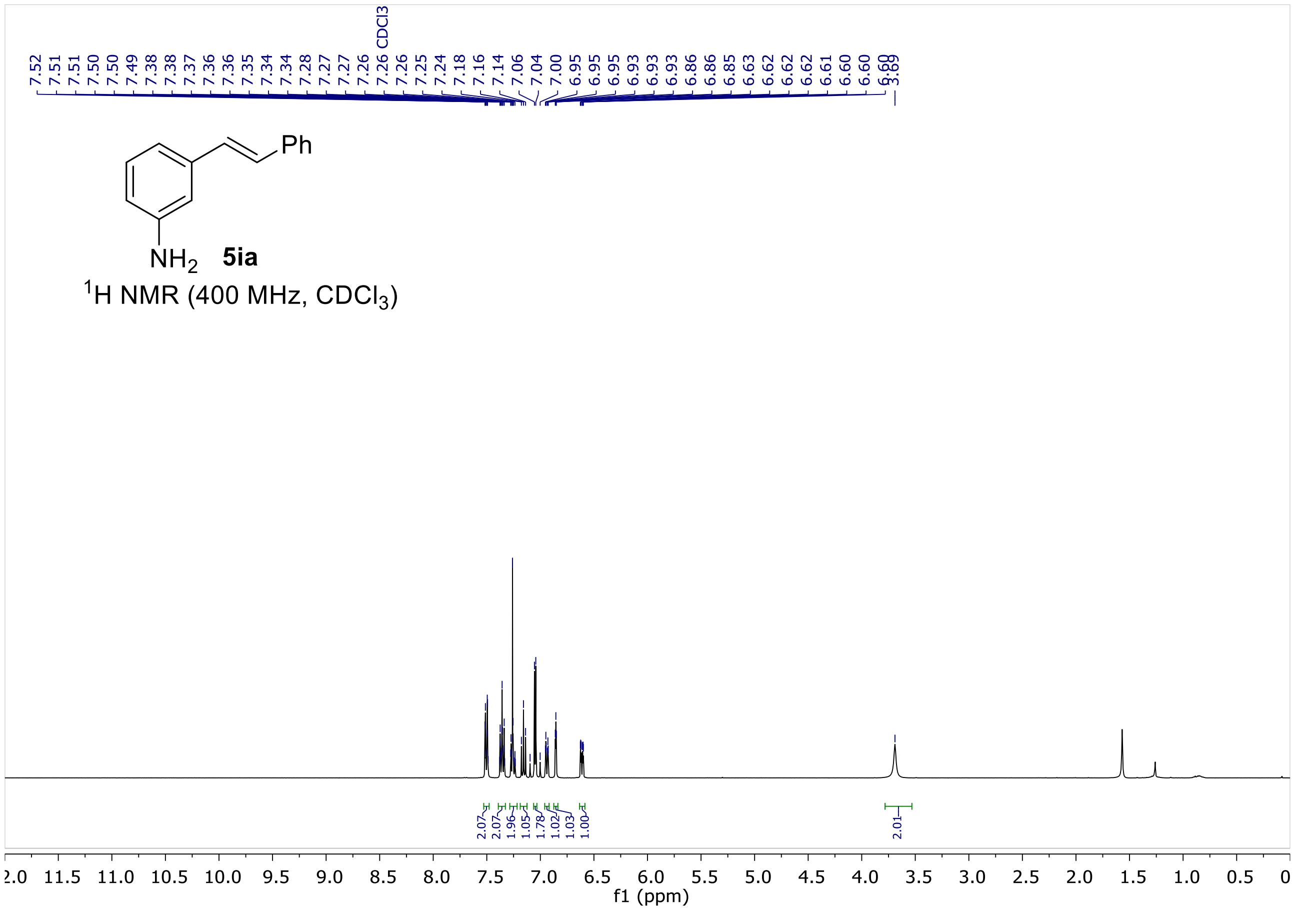

Ph
NH2
5ia
1H NMR (400 MHz, CDCl3)
f1 (ppm)


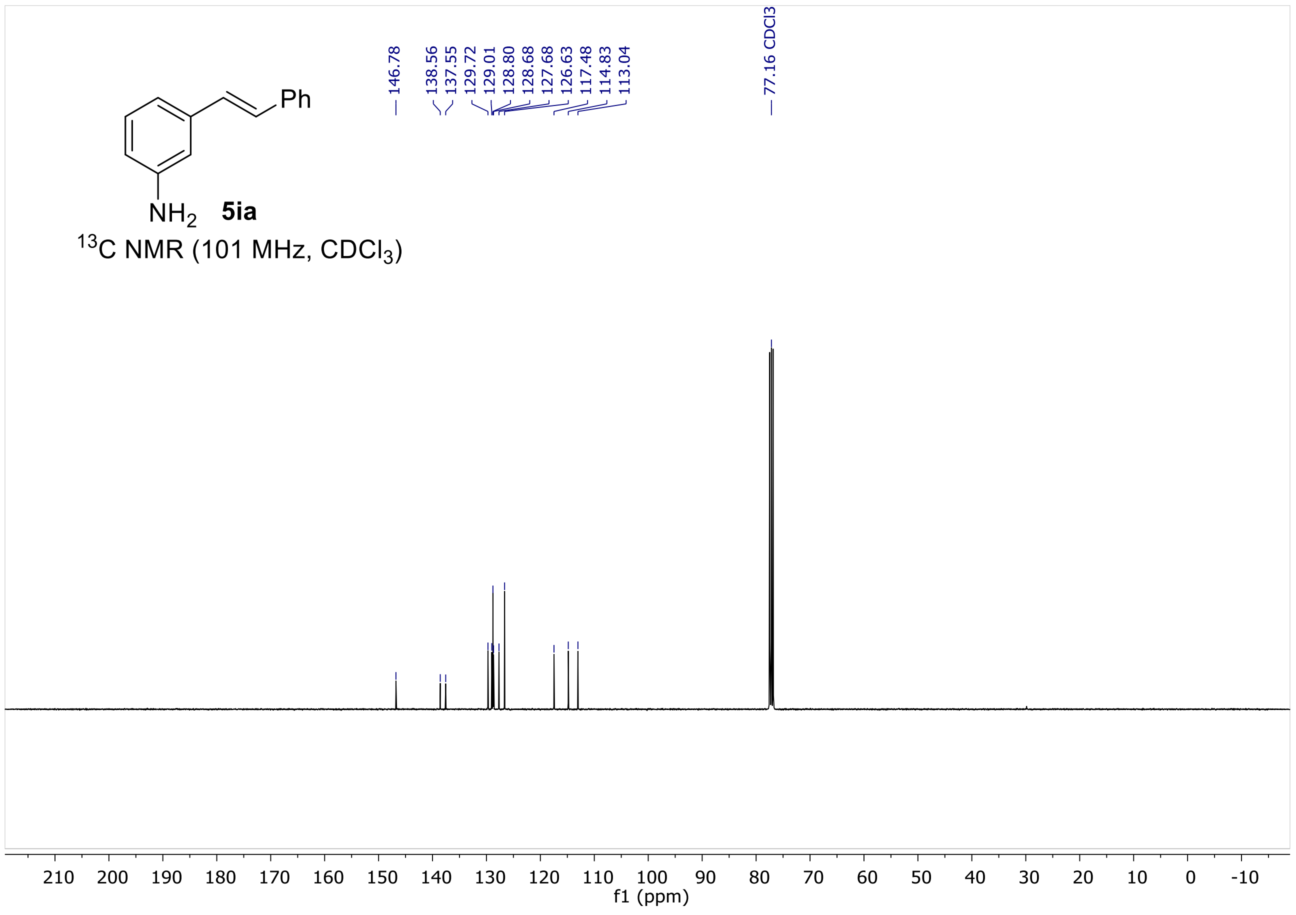

Ph
NH2
5ia
13C NMR (101 MHz, CDCl3)
f1 (ppm)

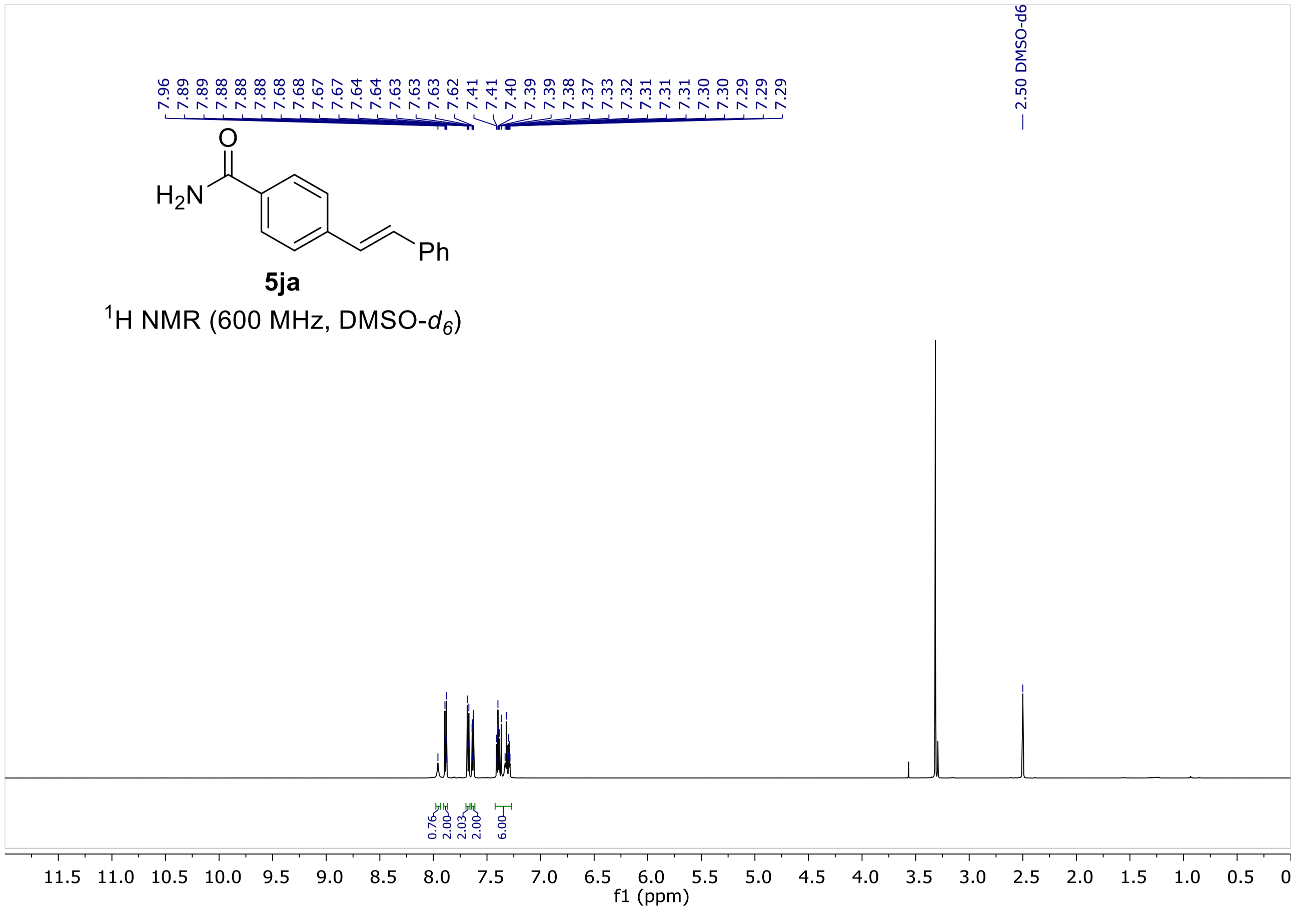
7.96 7.89 7.89 7.88 7.88 7.68 7.68 7.67 7.67 7.64 7.64 7.63 7.63 7.62 7.41 7.41 7.40 7.39 7.38 7.37 7.33 7.32 7.31 7.31 7.30 7.29 7.29 7.29
2.50 DMSO-d6
O
H2N
Ph
5ja
1H NMR (600 MHz, DMSO-d6)
0.76 2.00 2.03 2.00 6.00
11.5 11.0 10.5 10.0 9.5 9.0 8.5 8.0 7.5 7.0 6.5 6.0 5.5 5.0 4.5 4.0 3.5 3.0 2.5 2.0 1.5 1.0 0.5 0
f1 (ppm)

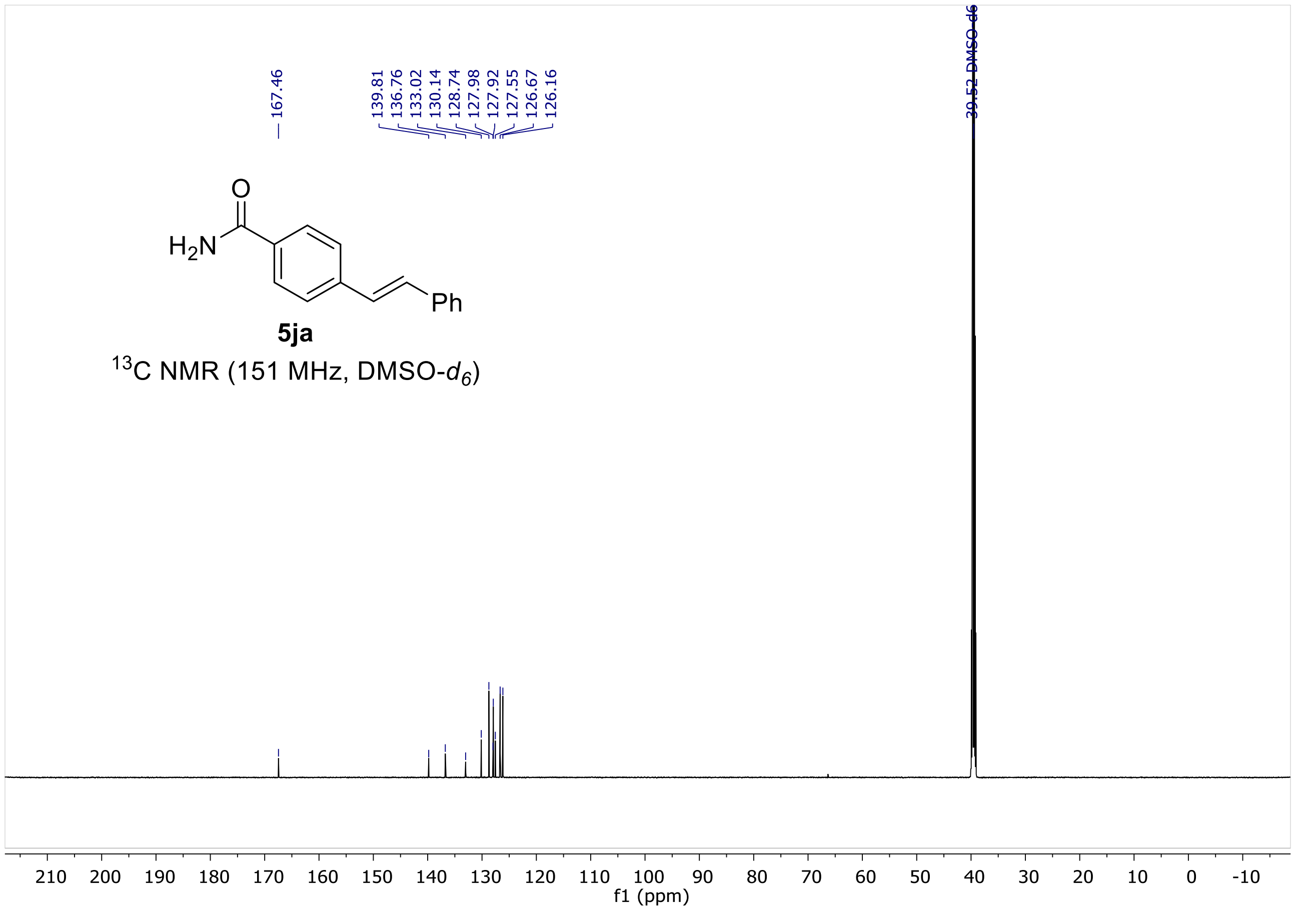
167.46
139.81 136.76 133.02 130.14 128.74 127.98 127.92 127.55 126.67 126.16
39.52 DMSO-d6
O
H2N
Ph
5ja
13C NMR (151 MHz, DMSO-d6)
210 200 190 180 170 160 150 140 130 120 110 100 90 80 70 60 50 40 30 20 10 0 -10
f1 (ppm)

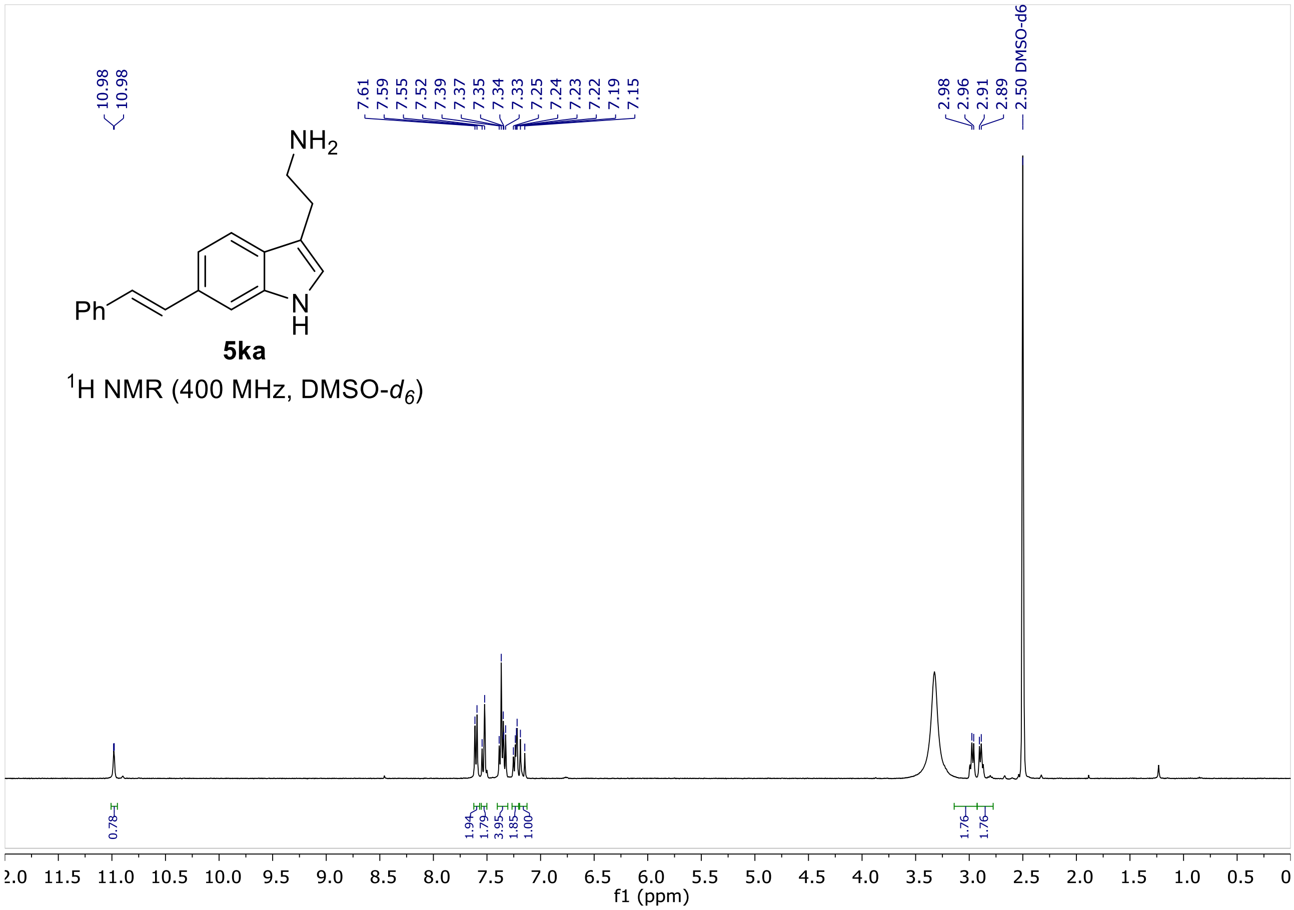

10.98
10.98
7.61
7.59
7.55
7.52
7.39
7.37
7.35
7.34
7.33
7.25
7.24
7.23
7.22
7.19
7.15
2.98
2.96
2.91
2.89
2.50 DMSO-d6
NH2
Ph
N
H
5ka
1H NMR (400 MHz, DMSO-d6)
0.78
1.94
1.79
3.95
1.85
1.00
1.76
1.76
f1 (ppm)


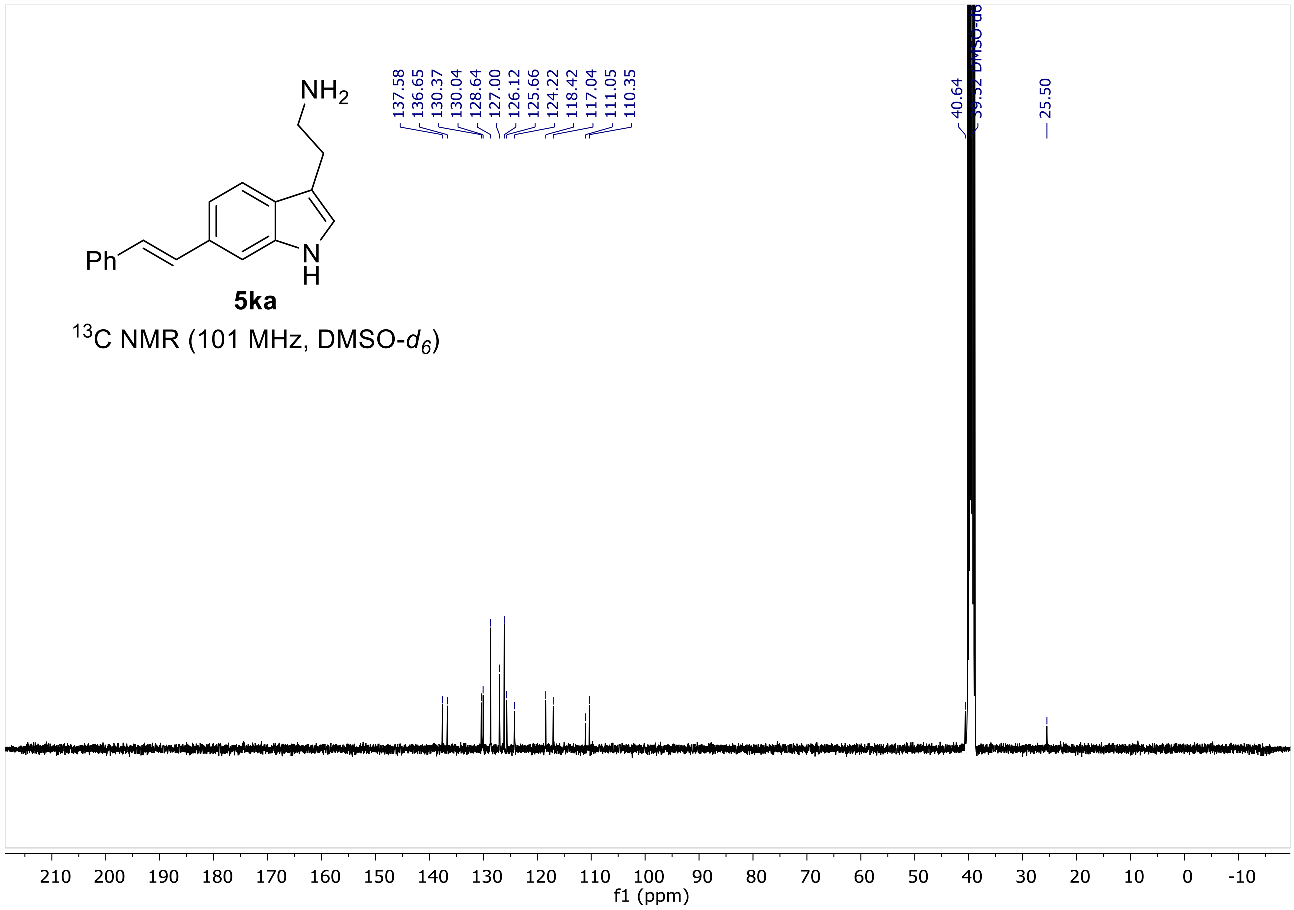

137.58
136.65
130.37
130.04
128.64
127.00
126.12
125.66
124.22
118.42
117.04
111.05
110.35
40.64
25.50
NH2
Ph
N
H
5ka
13C NMR (101 MHz, DMSO-d6)
f1 (ppm)

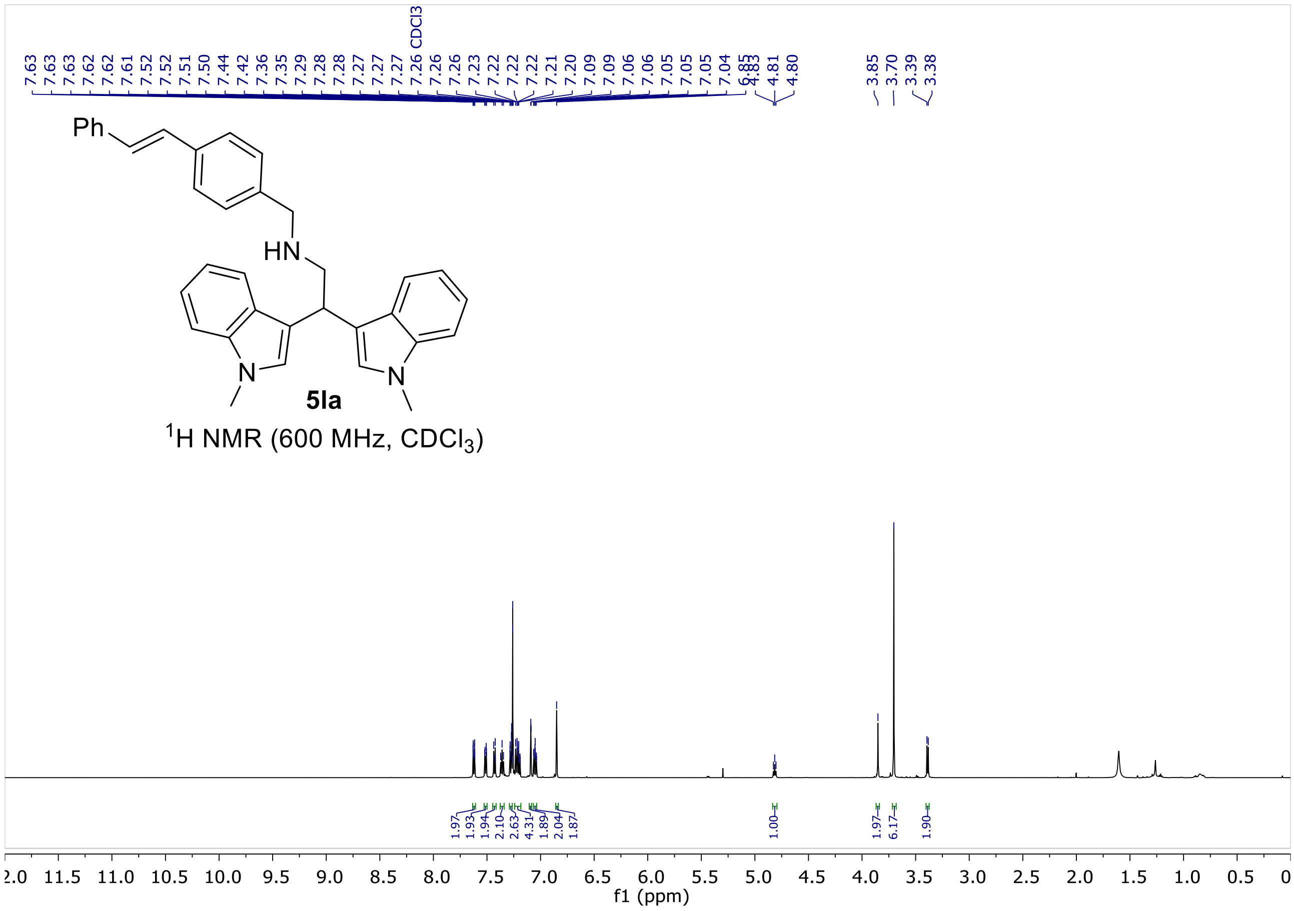

7.63
7.63
7.63
7.62
7.62
7.61
7.52
7.52
7.51
7.50
7.44
7.42
7.36
7.35
7.29
7.28
7.28
7.27
7.27
7.27
7.26 CDCl3
7.26
7.26
7.23
7.22
7.22
7.22
7.21
7.20
7.09
7.09
7.06
7.06
7.05
7.05
7.05
7.04
6.85
4.83
4.81
4.80
3.85
3.70
3.39
3.38
Ph
HN
N
N
5la
$^1$H NMR (600 MHz, CDCl$_3$)
1.97
1.93
1.94
2.10
2.63
4.31
1.89
2.04
1.87
1.00
1.97
6.17
1.90
f1 (ppm)


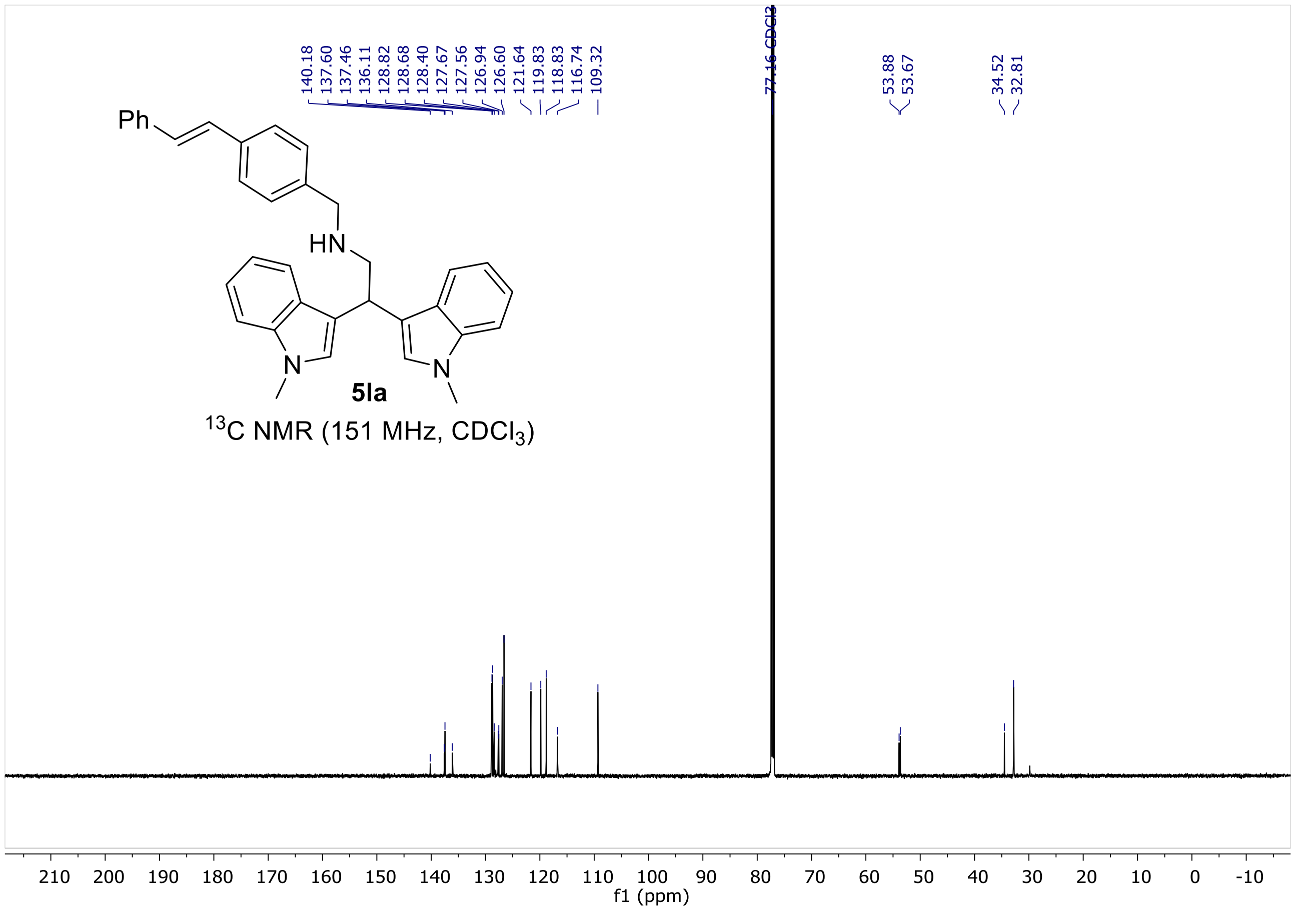

140.18
137.60
137.46
136.11
128.82
128.68
128.40
127.67
127.56
126.94
126.60
121.64
119.83
118.83
116.74
109.32
77.16 CDCl3
53.88
53.67
34.52
32.81
Ph
HN
N
N
5la
$^{13}$C NMR (151 MHz, CDCl$_3$)
f1 (ppm)

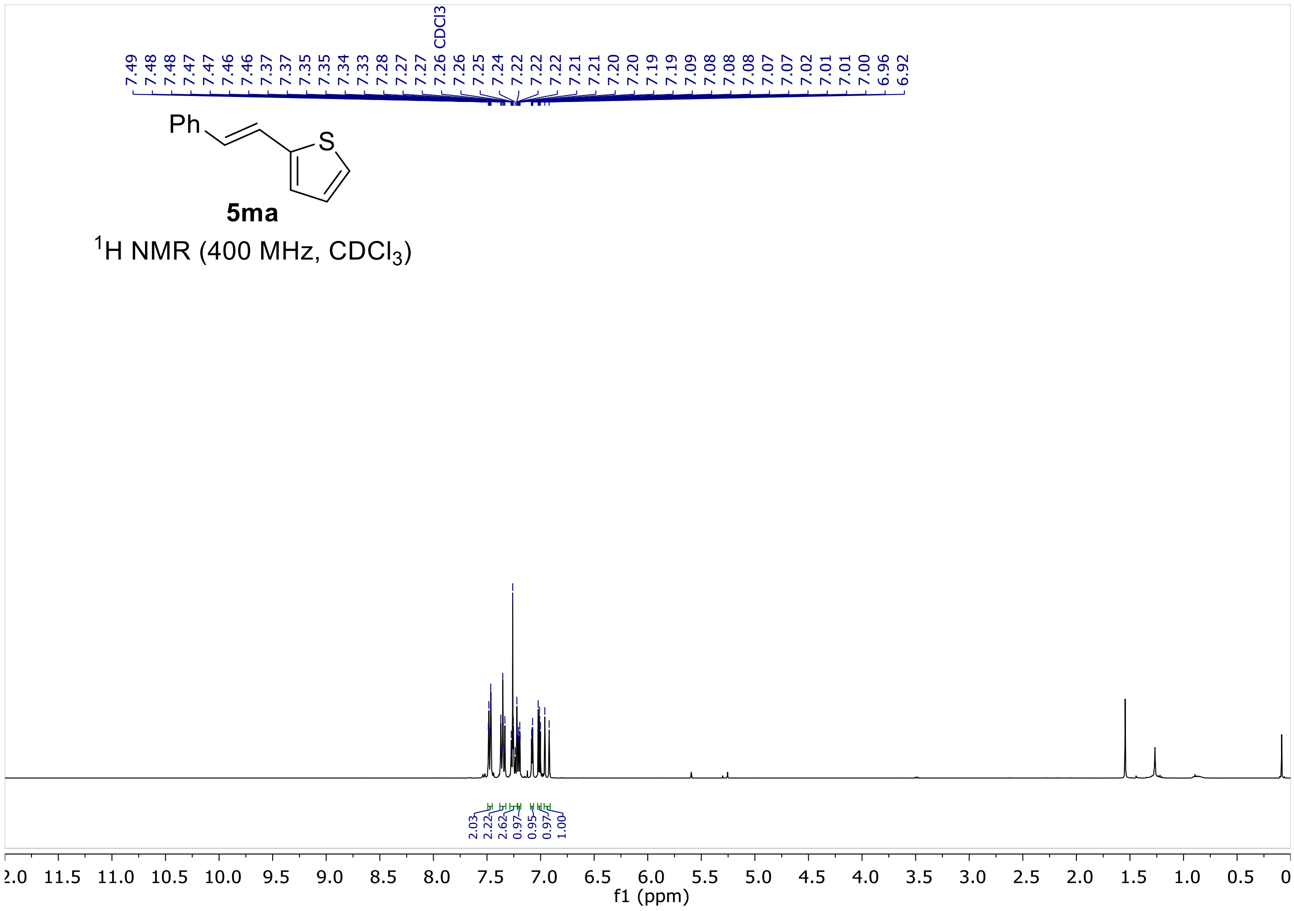

7.49
7.48
7.48
7.47
7.47
7.46
7.46
7.37
7.37
7.35
7.35
7.34
7.33
7.28
7.27
7.27
7.26 CDCl3
7.26
7.25
7.24
7.22
7.22
7.22
7.21
7.21
7.20
7.20
7.19
7.19
7.09
7.08
7.08
7.08
7.07
7.07
7.02
7.01
7.01
7.00
6.96
6.92
Ph
S
5ma
¹H NMR (400 MHz, CDCl3)
2.03
2.22
2.62
0.97
0.95
0.97
1.00
f1 (ppm)


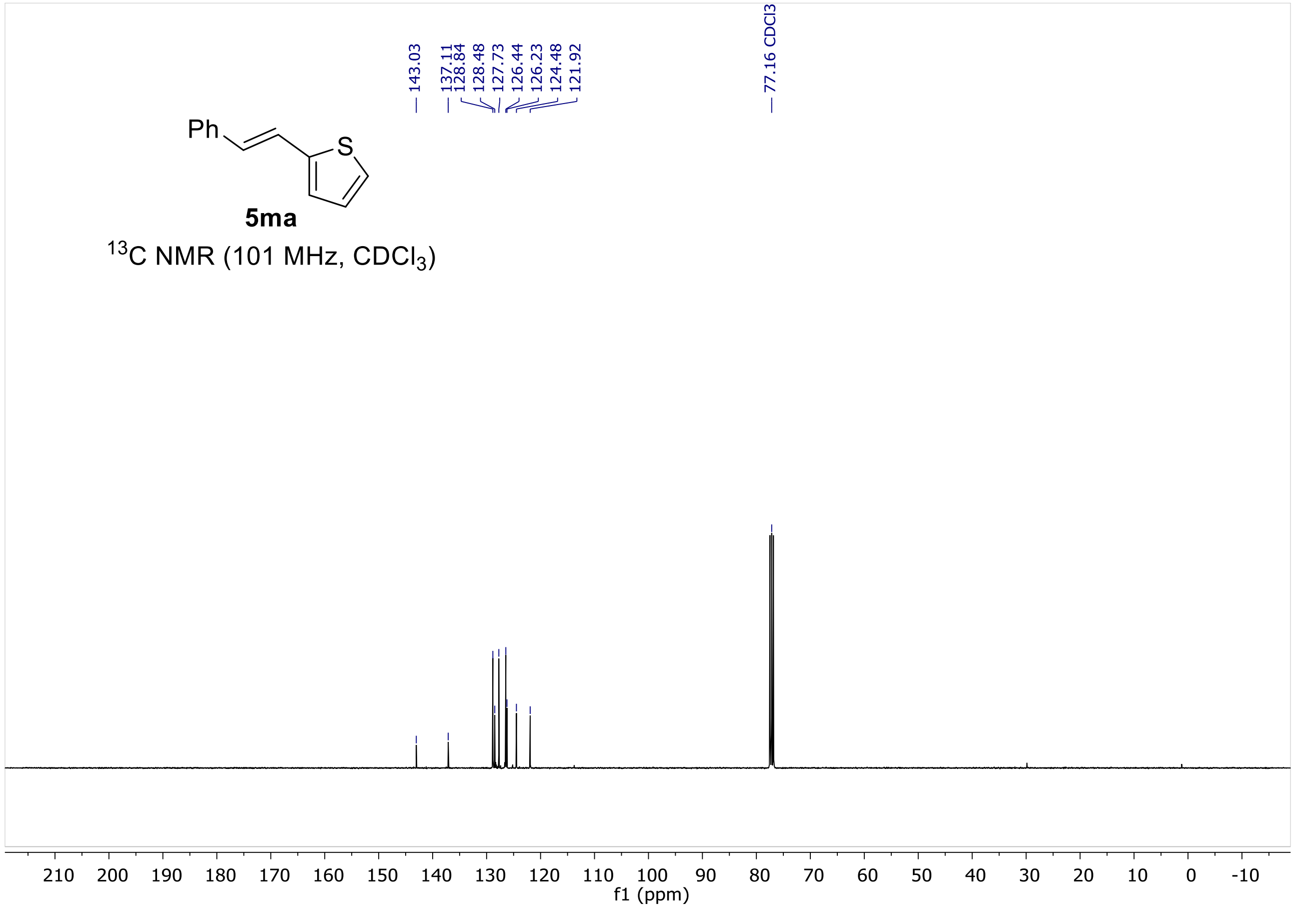

143.03
137.11
128.84
128.48
127.73
126.44
126.23
124.48
121.92
77.16 CDCl3
Ph
S
5ma
¹³C NMR (101 MHz, CDCl3)
f1 (ppm)

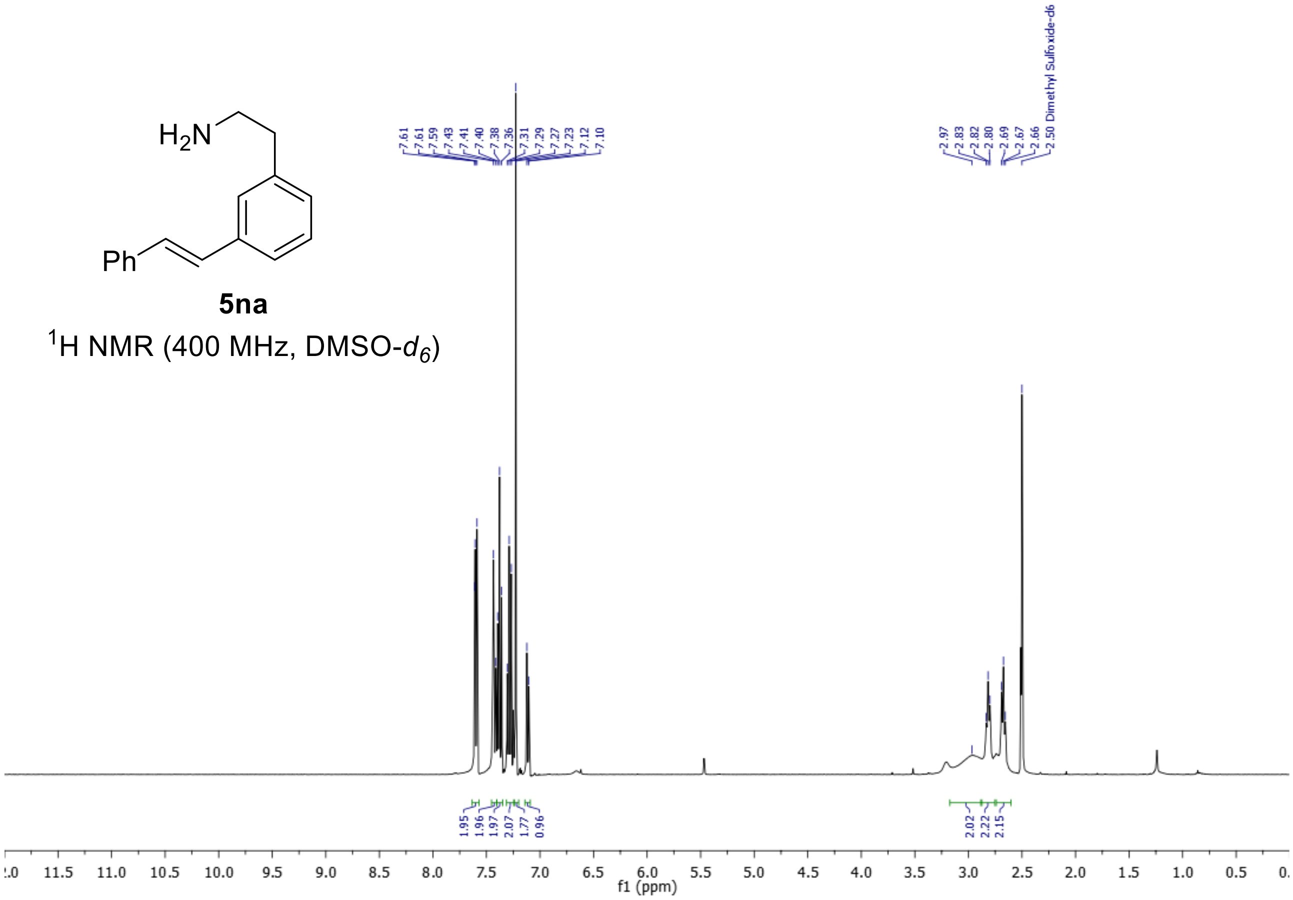


**5na**

$^{1}$H NMR (400 MHz, DMSO-$d_6$)

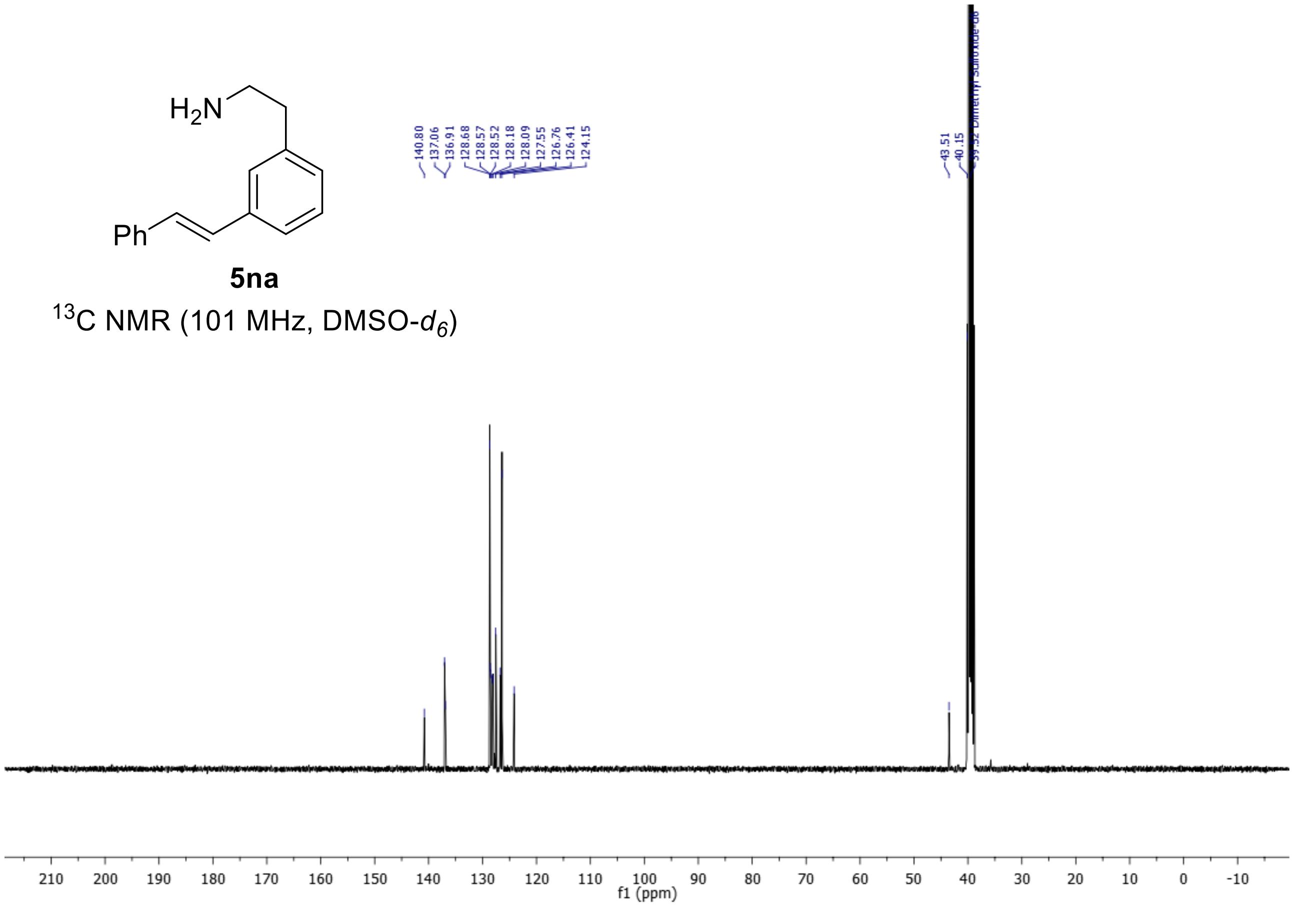


**5na**

$^{13}$C NMR (101 MHz, DMSO-$d_6$)

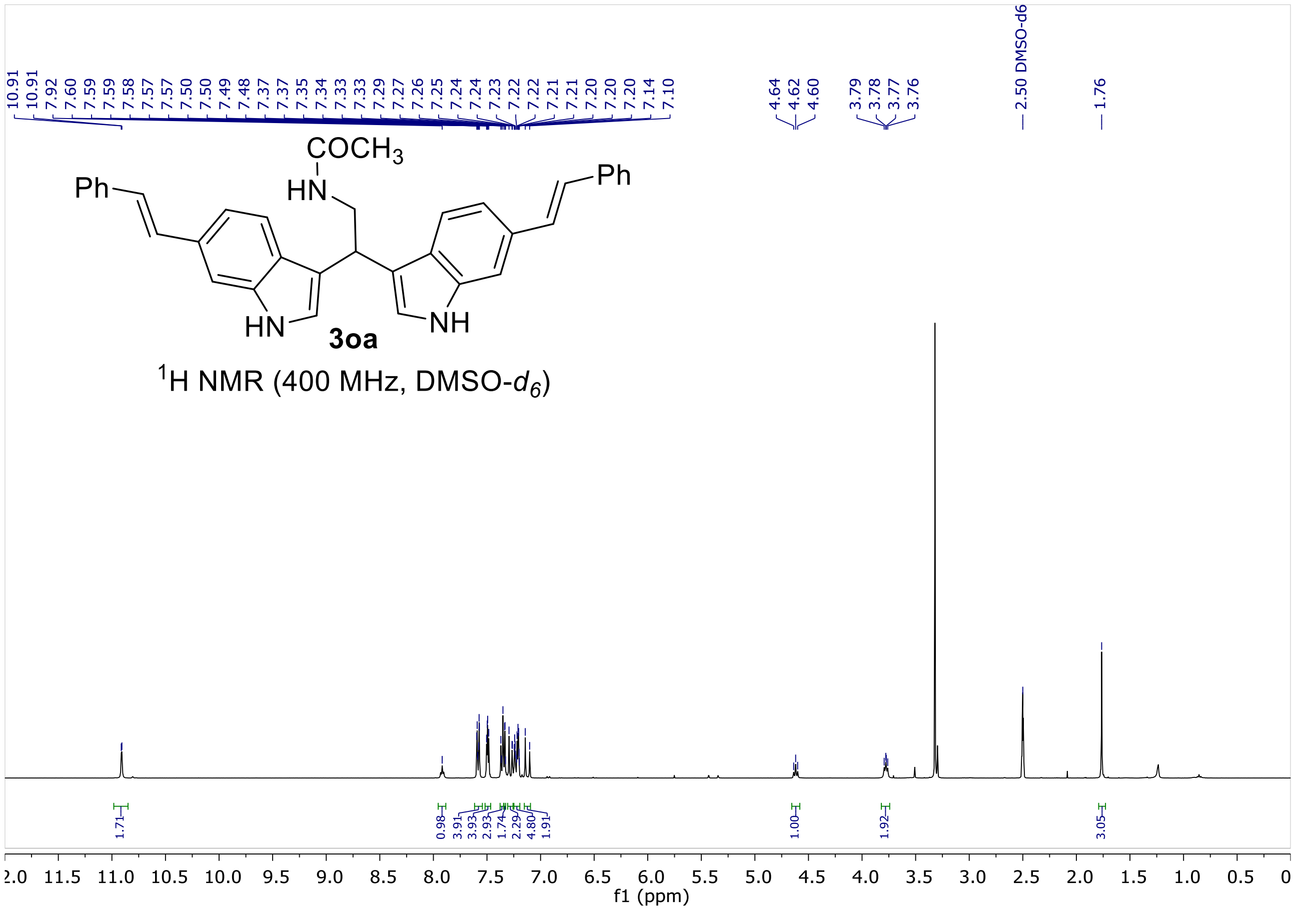
10.91 10.91 7.92 7.60 7.59 7.58 7.57 7.57 7.50 7.50 7.49 7.48 7.37 7.37 7.35 7.34 7.33 7.33 7.33 7.29 7.27 7.26 7.25 7.24 7.24 7.23 7.22 7.22 7.21 7.21 7.20 7.20 7.20 7.14 7.10
4.64 4.62 4.60
3.79 3.78 3.77 3.76
2.50 DMSO-d6
1.76
COCH3
HN
Ph
Ph
HN
NH
3oa
1H NMR (400 MHz, DMSO-d6)
1.71 0.98 3.91 3.93 2.93 1.74 2.29 4.80 1.91 1.00 1.92 3.05
f1 (ppm)

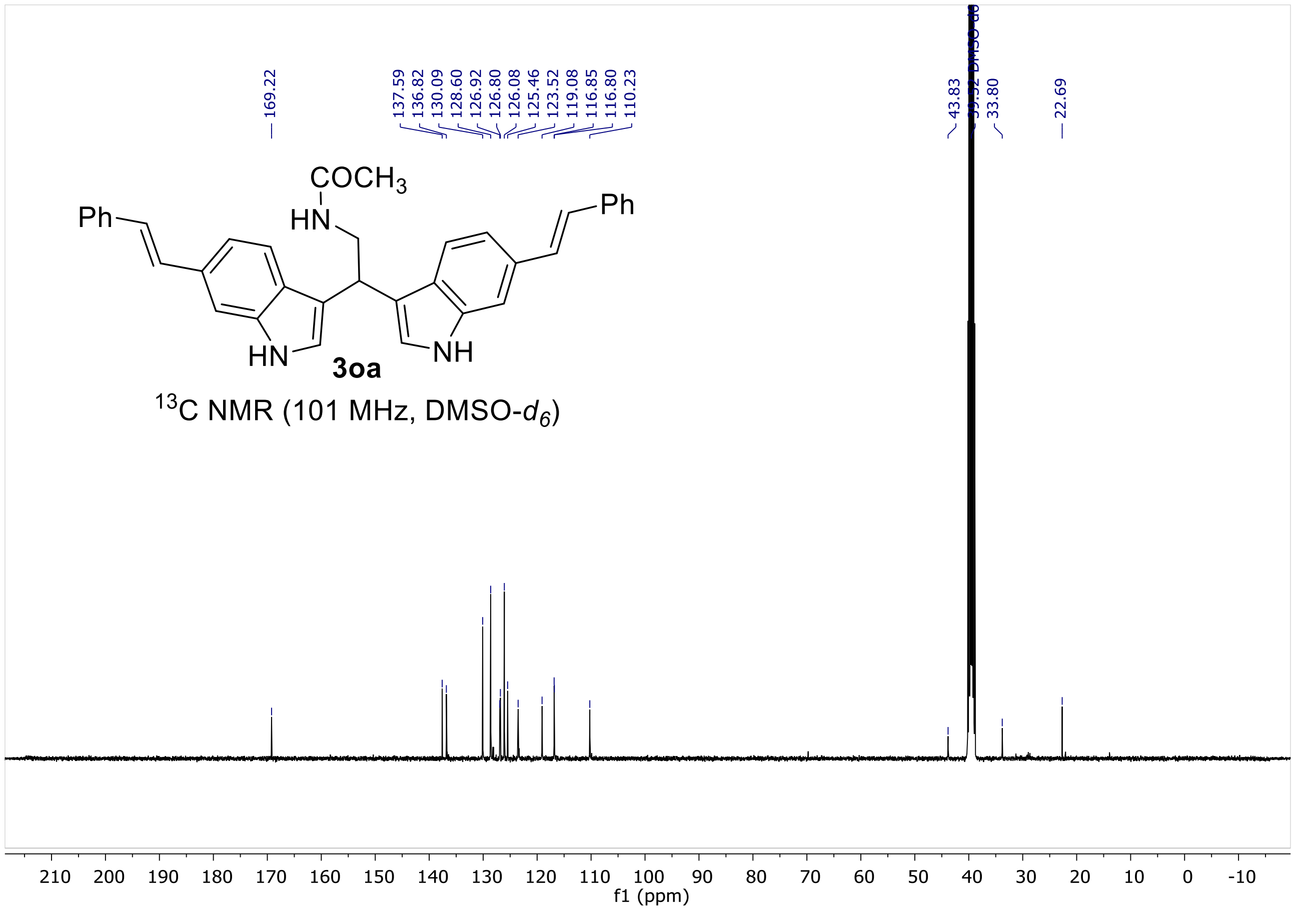
169.22
137.59 136.82 130.09 128.60 126.92 126.80 126.08 125.46 123.52 119.08 116.85 116.80 110.23
43.83
39.52 DMSO-d6
33.80
22.69
COCH3
HN
Ph
Ph
HN
NH
3oa
13C NMR (101 MHz, DMSO-d6)
f1 (ppm)

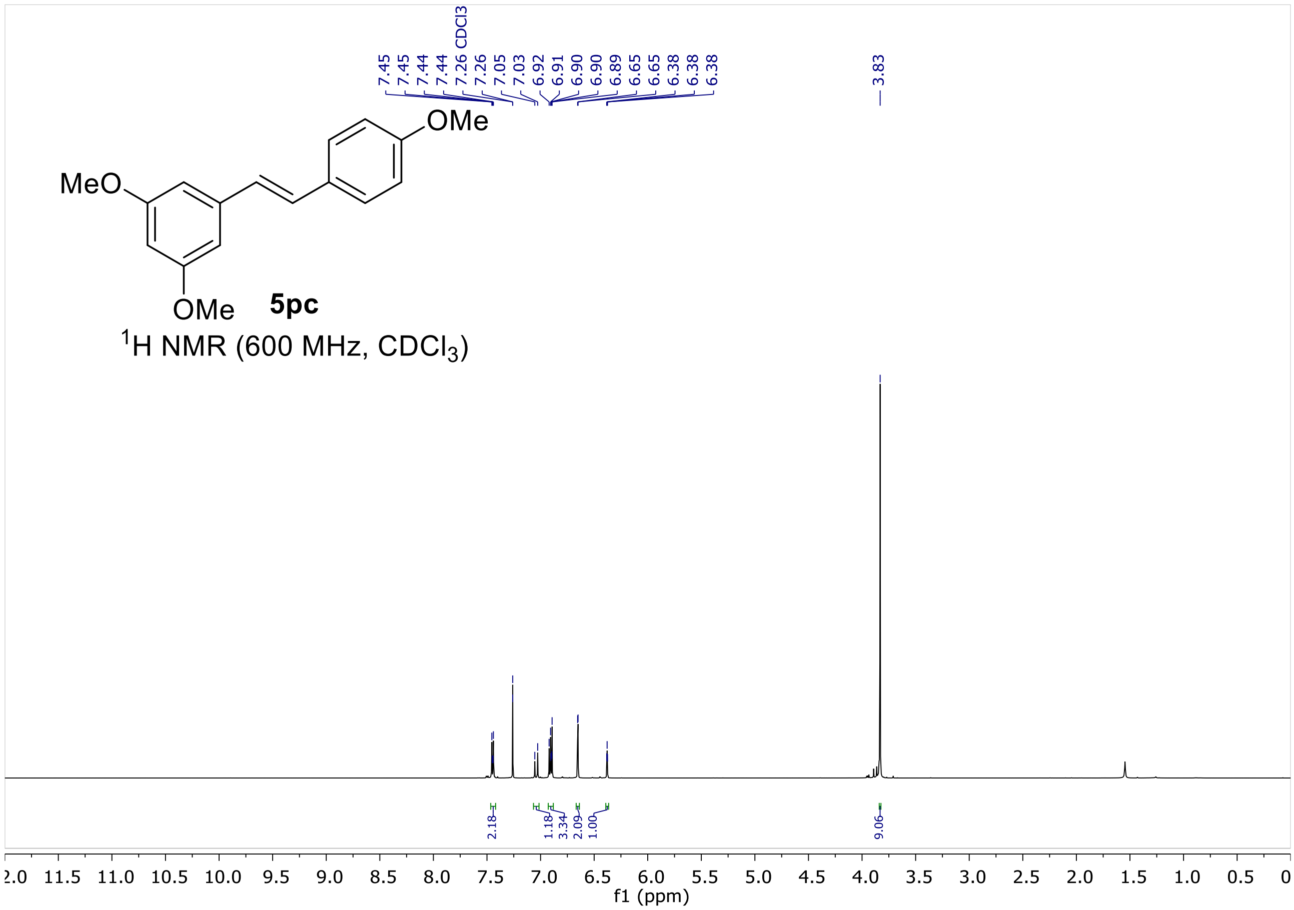
7.45
7.45
7.44
7.44
7.26 CDCl3
7.26
7.05
7.03
6.92
6.91
6.90
6.90
6.89
6.65
6.65
6.38
6.38
6.38
3.83
OMe
MeO
OMe
5pc
$^{1}$H NMR (600 MHz, CDCl$_3$)
2.18
1.18
3.34
2.09
1.00
9.06
f1 (ppm)

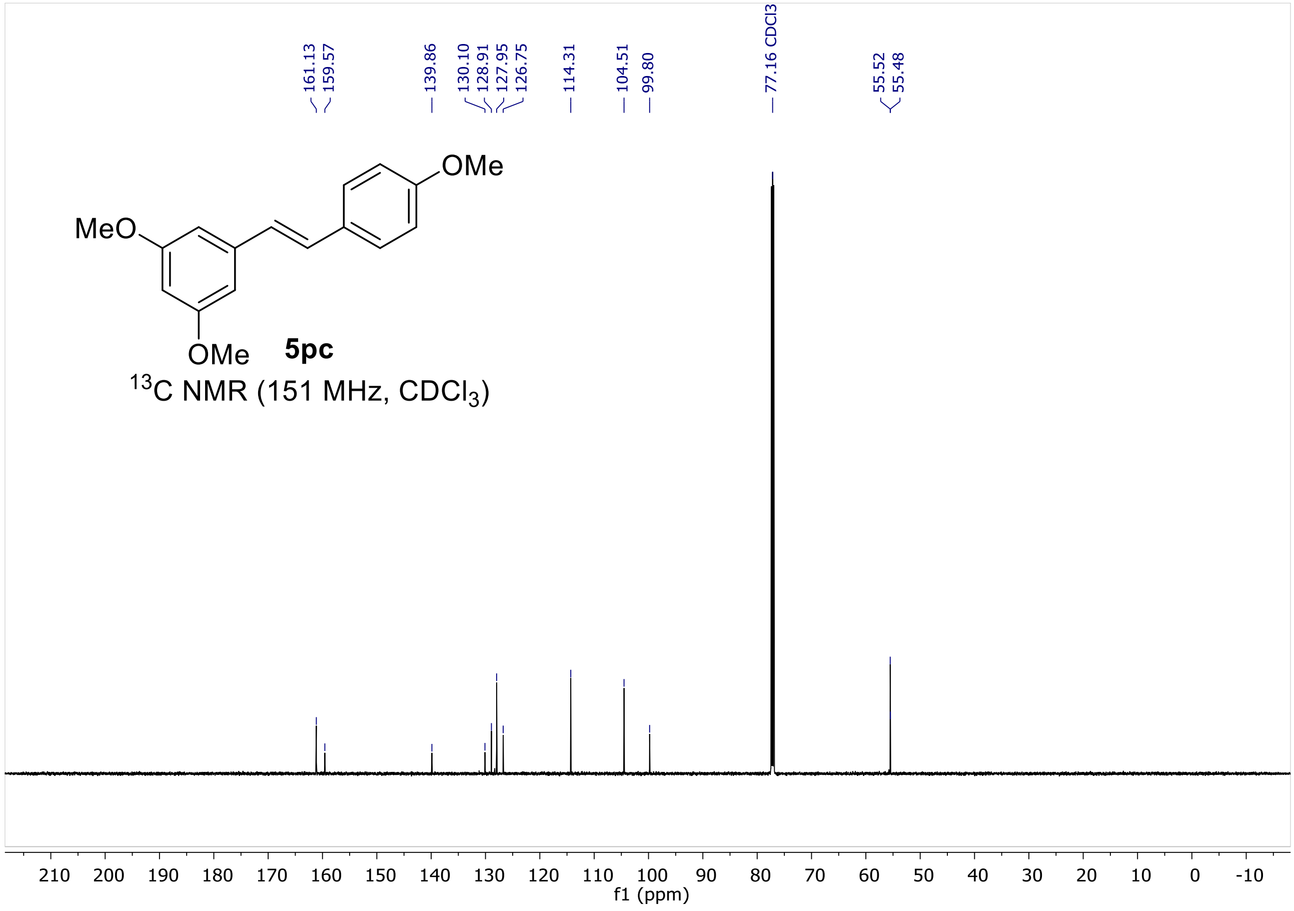
161.13
159.57
139.86
130.10
128.91
127.95
126.75
114.31
104.51
99.80
77.16 CDCl3
55.52
55.48
OMe
MeO
OMe
5pc
$^{13}$C NMR (151 MHz, CDCl$_3$)
f1 (ppm)

## 10. References


(1) Zhanga, P.; Wolf, C. Sensing of the concentration and enantiomeric excess of chiral compounds with tropos ligand derived metal complexes. *Chem. Commun.* **2013**, *49*, 7010-7012. https://doi.org/10.1039/C3CC43653E
(2) Sun, Q.; Escobar, L.; Ballester, P. A Dinuclear Metallobridged Super Aryl-Extended Calix[4]pyrrole Cavitand. *Angew. Chem. Int. Ed*. **2022**, *61*, e202202140. https://doi.org/10.1002/anie.202202140
(3) Linton, E. C.; Kozlowski, M. C. Catalytic Enantioselective Meerwein−Eschenmoser Claisen Rearrangement: Asymmetric Synthesis of Allyl Oxindoles. *J. Am. Chem. Soc.* **2008**, *130*, 16162–16163. https://doi.org/10.1021/ja807026z
(4) Mantenuto, S.; Lucarini, S.; De Santi, M.; Piersanti, G.; Brandi, G.; Favi, G.; Mantellini, F. One-Pot Synthesis of Biheterocycles Based on Indole and Azole Scaffolds Using Tryptamines and 1,2-Diaza-1,3-dienes as Building Blocks. *Eur. J. Org. Chem*. **2016**, 3193–3199. https://doi.org/10.1002/ejoc.201600210
(5) Mari, M.; Tassoni, A.; Lucarini, S.; Fanelli, M.; Piersanti, G.; Spadoni, G. Brønsted Acid Catalyzed Bisindolization of α-Amido Acetals: Synthesis and Anticancer Activity of Bis(indolyl)ethanamino Derivatives. Eur. *J. Org. Chem*. **2014**, 3822-3830. https://doi.org/10.1002/ejoc.201402055
(6) Buono, A.; Diotallevi, A.; Maestrini, S.; Verboni, M.; Kiuru, P.; Galluzzi, L.; Duranti, A.; Olivieri, D.; Lucarini, S. Efficient and Rapid Arylation of $NH_2$-Unprotected Bromobisindole Ethanamines via Suzuki-Miyaura Coupling: Generating New Leads against Leishmania. *Chem. Eur. J.* **2025**, *31*, e202500637. https://doi.org/10.1002/chem.202500637
(7) Schumachert, R. W.; Davidson, B. S. Synthesis of didemnolines A-D, N9-substituted β-carboline alkaloids from the marine ascidian *Didemnum* sp. *Tetrahedron* **1999**, *34*, 935.
(8) Centanni, A.; Diotallevi, A.; Buffi, G.; Olivieri, D.; Santarém, N.; Lehtinen, A.; Yli-Kauhaluoma, J.; Cordeiro-da-Silva, A.; Kiuru, P.; Lucarini, S.; Galluzzi, L. Exploring hydrophilic 2,2-di(indol-3-yl)ethanamine derivatives against Leishmania infantum. *PLoS One* **2024**, *19*, e0301901. https://doi.org/10.1371/journal.pone.0301901
(9) Warren, H. T.; Saeger, H. N.; Tombari, R. J.; Chytil, M.; Rasmussen, K.; Olson, D. E. Psychoplastogenic DYRK1A Inhibitors with Therapeutic Effects Relevant to Alzheimer's Disease. *J. Med. Chem.* **2024**, *67*, 6922–6937. https://doi.org/10.1021/acs.jmedchem.3c01696
(10) Ren, X.; Lu, P.; Zheng, C.; Wang, Y.; Lu, Z. Cobalt-Catalyzed Stereodivergent Semihydrogenation of Alkynes: Synthesis of E- and Z-Alkenes. *Angew. Chem. Int. Ed*. **2025**, *64*, e202511269. https://doi.org/10.1002/anie.202511269
(11) Jia, X.; Frye, L. I.; Zhu, W.; Gu, S.; Gunnoe, T. B. Synthesis of Stilbenes by Rhodium-Catalyzed Aerobic Alkenylation of Arenes via C–H Activation. *J. Am. Chem. Soc.* **2020**, *142*, 10534–10543. https://doi.org/10.1021/jacs.0c03935
(12) Shi, W.; Yu, J.; Jiang, Z.; Shao, Q.; Su, W. Encaging palladium(0) in layered double hydroxide: A sustainable catalyst for solvent-free and ligand-free Heck reaction in a ball mill. *Beilstein J. Org. Chem.* **2017,** *13,* 1661–1668. https://doi.org/10.3762/bjoc.13.160
(13) Puthiaraja, P.; Pitchumani, K. Palladium nanoparticles supported on triazine functionalised mesoporous covalent organic polymers as efficient catalysts for Mizoroki–

Heck cross coupling reaction. *Green Chem.* **2014**, *16*, 4223-4233. https://doi.org/10.1039/C4GC00412D
(14) Subaramanian, M.; Padhy, S. S.; Gouda, C.; Das, T.; Vanka, K.; Balaraman, E. Nickel-catalyzed tandem conversion of paraformaldehyde : methanol to hydrogen and formate/chemo- and stereoselective hydrogenation of alkynes under neutral conditions. *Catal. Sci. Technol.*, **2024**, *14*, 2779-2793. https://doi.org/10.1039/D3CY01699D
(15) Guo, S.-M.; Xu, P.; Studer, A. *Meta*-Selective Copper-Catalyzed C−H Arylation of Pyridines and Isoquinolines through Dearomatized Intermediates. *Angew. Chem. Int. Ed*. **2024**, *63*, e202405385. https://doi.org/10.1002/anie.202405385
(16) Chen, Z.; Luo, M.; Wen, Y.; Luo, G.; Liu, L. Transition-Metal-Free Semihydrogenation of Diarylalkynes: Highly Stereoselective Synthesis of trans-Alkenes Using $Na_2S·9H_2O$. *Org. Lett.* **2014**, *16*, 3020–3023. https://doi.org/10.1021/ol501137x
(17) Tabaru, K.; Nishimura, H.; Suzuki, T.; Obora, Y. Palladium nanoparticles for aerobic oxidative Heck arylation of alkenes. *Chem. Lett.* **2025**, *54*, upaf068. https://doi.org/10.1093/chemle/upaf068
(18) Jiang, W.; Leng, X.; Yang, Y.; Lu, L.; Shen, Q. CageCarbene-Ir Complex Enables Selective Alkylation and Alkenylation of Benzyl Alcohols with Alkyl Sulfones. *Chin. J. Chem.* **2025**, *43*, 3582—3588. https://doi.org/10.1002/cjoc.70297
